# Seasonal habitat use and diel vertical migration in female spurdog in Nordic waters

**DOI:** 10.1186/s40462-024-00498-2

**Published:** 2024-09-06

**Authors:** C. Antonia Klöcker, Ole Thomas Albert, Keno Ferter, Otte Bjelland, Robert J. Lennox, Jon Albretsen, Lotte Pohl, Lotte Svengård Dahlmo, Nuno Queiroz, Claudia Junge

**Affiliations:** 1 Havforskningsinstituttet (Institute of Marine Research, IMR), P.O. Box 1870, 5817 Nordnes, Bergen, Norway; 2 Ocean Tracking Network, Dalhousie University, 1355 Oxford St, Halifax, NS Canada; 3 VLIZ, Flanders Marine Institute, Marine Observation Centre, Jacobsenstraat 1, 8400 Ostend, Belgium; 4 Laboratory for Freshwater Ecology and Inland Fisheries, NORCE Norwegian Research Centre, Nygardsgaten 112, 5008 Bergen, Norway; 5 CIBIO, Centro de Investigação em Biodiversidade e Recursos Genéticos, InBIO Laboratório Associado, Campus de Vairão, Universidade do Porto, 4485-661 Vairão, Portugal; 6 BIOPOLIS Program in Genomics, Biodiversity and Land Planning, CIBIO, Campus de Vairão, 4485-661 Vairão, Portugal

**Keywords:** Biologging, Archival tag, PSAT, Sharks, DVM, Wavelet analysis, Fast starts, Niche, Fishery, North-East Atlantic

## Abstract

**Background:**

Studying habitat use and vertical movement patterns of individual fish over continuous time and space is innately challenging and has therefore largely remained elusive for a wide range of species. Amongst sharks, this applies particularly to smaller-bodied and less wide-ranging species such as the spurdog (*Squalus acanthias* Linnaeus, 1758), which, despite its importance for fisheries, has received limited attention in biologging and biotelemetry studies, particularly in the North-East Atlantic.

**Methods:**

To investigate seasonal variations in fine-scale niche use and vertical movement patterns in female spurdog, we used archival data from 19 pregnant individuals that were satellite-tagged for up to 365 days in Norwegian fjords. We estimated the realised niche space with kernel densities and performed continuous wavelet analyses to identify dominant periods in vertical movement. Triaxial acceleration data were used to identify burst events and infer activity patterns.

**Results:**

Pregnant females frequently utilised shallow depths down to 300 m at temperatures between 8 and 14 °C. Oscillatory vertical moments revealed persistent diel vertical migration (DVM) patterns, with descents at dawn and ascents at dusk. This strict normal DVM behaviour dominated in winter and spring and was associated with higher levels of activity bursts, while in summer and autumn sharks predominantly selected warm waters above the thermocline with only sporadic dive and bursts events.

**Conclusions:**

The prevalence of normal DVM behaviour in winter months linked with elevated likely foraging-related activity bursts suggests this movement behaviour to be foraging-driven. With lower number of fast starts exhibited in warm waters during the summer and autumn months, habitat use in this season might be rather driven by behavioural thermoregulation, yet other factors may also play a role. Individual and cohort-related variations indicate a complex interplay of movement behaviour and habitat use with the abiotic and biotic environment. Together with ongoing work investigating fine-scale horizontal movement as well as sex- and age-specific differences, this study provides vital information to direct the spatio-temporal distribution of a newly reopened fishery and contributes to an elevated understanding of the movement ecology of spurdog in the North-East Atlantic and beyond.

**Graphical Abstract:**

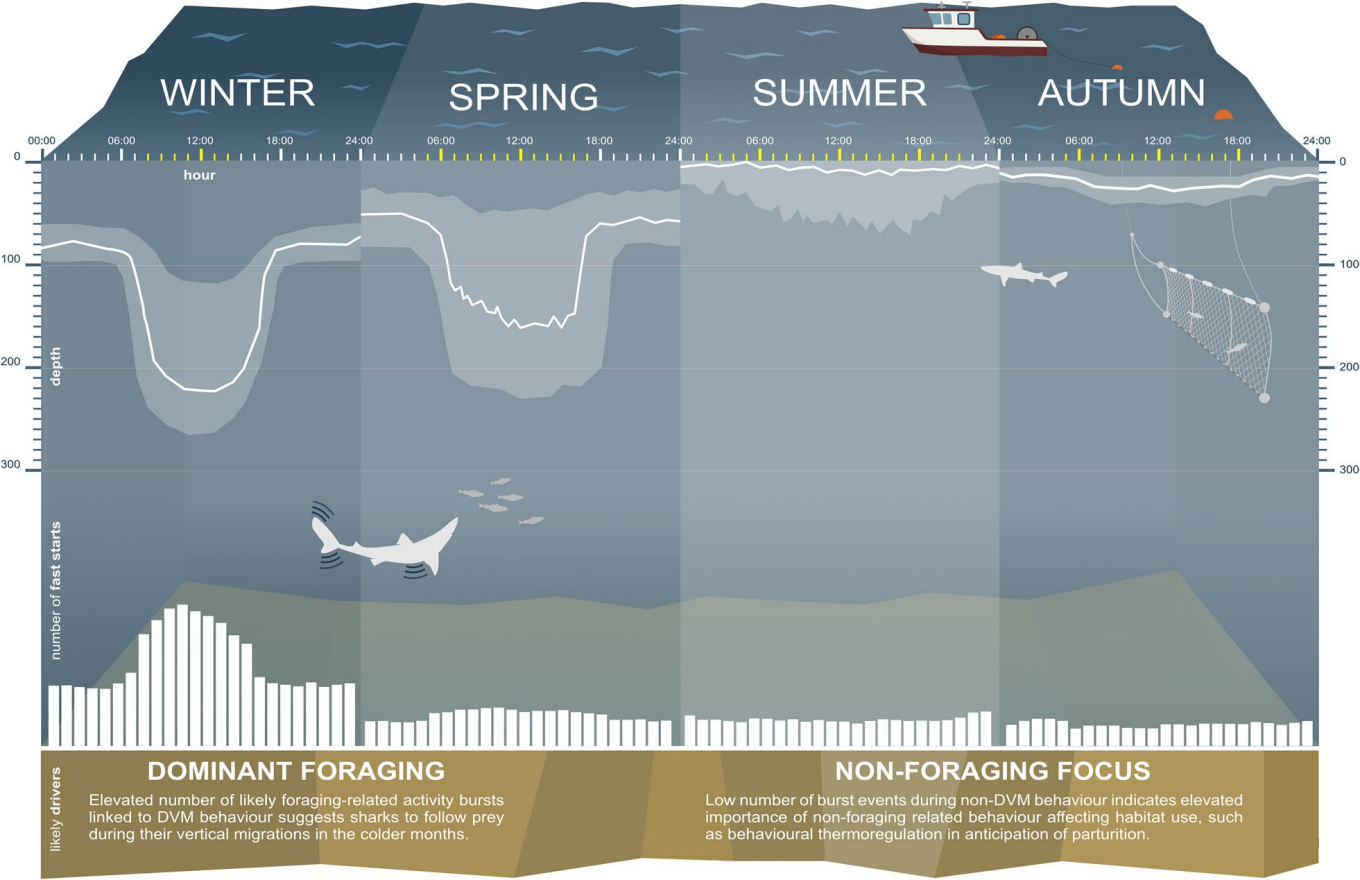

**Supplementary Information:**

The online version contains supplementary material available at 10.1186/s40462-024-00498-2.

## Background

Sharks display a variety of movements, which are not restricted to the two-dimensional plane but extend into the third dimension, depth. Such three-dimensional movements connect at times very disjunct ecosystems by transferring organic matter [1] and not only determine the fate of individuals but also shape the structure and dynamics of populations, communities, and ecosystems [2]. Recent studies have re-emphasised the importance of this third dimension for many sharks, rays and skates (elasmobranchs) as depth use and vertical mobility shape the ecological role of an animal and affect its survival, fitness as well as exposure and resilience to anthropogenic threats such as fishing and climate change [3–8].

Vertical movement is thought to be driven by the need to optimise foraging and energy expenditure, while remaining within physiological limits imposed by abiotic factors, such as ambient water temperature and dissolved oxygen levels, to ultimately ensure growth, survival, and reproduction [3, 9, 10]. Thus, common hypotheses to explain observed depth use and vertical mobility include efficient foraging, locomotion or thermoregulation [11–15].

A common vertical movement pattern across marine predators such as elasmobranchs is diel vertical migration (DVM), which is often associated with foraging as sharks follow the daily migration of zooplankton, mesopelagic fish, and associated predators. The classical or normal DVM (nDVM) pattern is characterized by a dusk ascent towards the surface and a dawn descent to the mesopelagic, triggered by the evasion of visual predators in well-lit surface waters [16–18]. However, variations in DVM behaviour are often observed such as reversed DVM (rDVM), where species like some sharks are found in shallower waters during the day and deeper waters during the night, which can be linked to spatio-temporal variations in prey distribution [3, 19]. Depending on the physiological tolerance of a species, diel vertical patters can range on a continuum from highly oscillatory swimming with a diel pattern to strict DVM [9]. With oscillatory swimming we refer to repeated dives to a given depth either during day or night as observed in blue sharks *(Prionace glauca)* or yellowfin tuna (*Thunnus albacares)* [11, 20], while with strict DVM behaviour we imply a consistent use of a preferred depth during day and night as described for example for big-eye tuna (*Thunnus obesus*) [20].

Biologging and biotelemetry have become key tools to study such individual movements in space and time [21–23]. Technological advancements in the last decades have facilitated the development of electronic tags which autonomously transmit data without the need to resight or recapture the animal. In the case of pop-up archival transmitting tags (PAT tags or PSATs), depth, temperature, light-level, and depending on the manufacturer and model also triaxial acceleration data are continuously archived. After a programmed release, summaries of this data are relayed via the Argos system (http://www.argossystem.org/). If tags are physically recovered, researchers obtain access to the full data archive comprising the data streams highlighted above at a resolution of minutes to seconds, depending on tag model and deployment time. Such animal-borne data can be used to reconstruct occupied environmental niche spaces of individuals or populations by building multivariate environmental envelopes, e.g. depth-temperature spaces, using kernel density or principal components [24, 25]. Further, continuous time series that are provided by biologging devices allow analysis of periodicity in movement using signal processing methods such as continuous wavelet analysis. In contrast to a Fast Fourier transformation (e.g., [26–29]), wavelet transformations retain temporal data with the wave frequencies, such that episodes of cyclical behaviour can be identified from archived depth or acceleration data (e.g., [30–32]). Besides cyclical behaviours, instances of discrete behaviours may be identified from accelerometers. Triaxial accelerometers can sample at very high frequencies in three spatial dimensions to identify different behavioural states. Foraging or predator–prey escape responses, for example, are often characterised by sudden bursts in acceleration, so-called fast starts [33, 34]. Fast starts have been studied in a range of species from bottom-dwelling fish such as the great sculpin (*Myoxocephalus polyacanthocephalus*) to pelagic high-performance swimmers such as yellowfin tuna [35–40].

While in the past decades research efforts in the field of satellite telemetry have focused on large-bodied (> 3 m up to > 10 m) and wide-ranging species such as whale sharks (*Rhincodon typus*), tiger sharks (*Galeocerdo cuvier*), white sharks (*Carcharodon carcharias*), or blue sharks [41, 42], advancements in the miniaturisation of tags have made it possible to track smaller, and also slimmer, commercially important species such as spurdog (*Squalus acanthias* Linnaeus, 1758) with a common length of about 1 m [43–47]. Spurdog, also known as piked dogfish and spiny dogfish, is circumglobally distributed and predominantly occurs in temperate waters of the Atlantic and Pacific oceans between 20 and 300 m depth, but found down to 900 m [48–51]. In the North-East Atlantic (NEA), its northern distribution limit extents to Norway and Iceland [52]. In Norway, the combination of (i) a complex coastal landscape consisting of deep coastal fjords commonly extending to 650 m depth (e.g. Osterfjorden) and a maximum depth of 1,300 m in Sognefjorden, (ii) the offshore Norwegian Trench, and (iii) the relatively shallow North Sea make the area dynamic and conductive to the formation of local populations with possibly distinct environmental niches and movement dynamics.

Having mainly been subject of conventional tagging efforts as well as catch-based data in the NEA and North-West Atlantic (NWA) [48, 53–61], fine-scale vertical movement patterns in spurdog remain to be resolved. Although fishery-dependent data are available, they are limited to areas and seasons spurdog is fished in and depend on reporting. Beyond broad ranges of depth and temperature use, often inferred from bottom trawl surveys [50, 62–64], little is known about the environmental niche of individuals and related temporal patterns [65]. Existing studies from the U.S. east-coast as well as Scotland using electronic telemetry [44, 47, 66] have provided first indication that spurdog display DVM behaviour, yet have suggested location- and cohort-specific habitat use and movements across the species’ latitudinal range [44, 53, 67, 68].

Late maturity, slow gestation [69–74], sexual dimorphism, and gregarious behaviour [50, 51, 67, 75–77] make spurdog particularly vulnerable to overfishing and bycatch [43, 78, 79]. Due to regulations preventing targeted fishing, spurdog was recently moved from *Endangered* to *Vulnerable* on the Norwegian Red List [80]. With an improving status in the NEA and ICES recommending the first catch advice in the NEA since 2009 [46] there is a great need to better understand the movement ecology of this shark to attenuate the increasing occurrence of conflicts with fishers and fish farmers.

After previous difficulties related to tag attachment, a recent telemetry study successfully deployed 19 PSATs for up to 365 days on adult females in the fjord systems in western Norway between 2019 and 2023 (Junge et al., in review). Junge et al. (in review) identified coastal fjord systems as key habitats for pregnant female spurdog and highlighted significant differences in inferred depth-temperature niches obtained from individual-based tagging data and catch-based data from bottom trawls and longlines.

Building upon this archival dataset with continuous 0.2 Hz time series of depth, temperature, light level, and triaxial acceleration for 4,612 cumulative days across 19 individuals, this study wants to shed light on patterns of individual depth and temperature use, as well as vertical movement, which to this date have remained poorly understood for spurdog, particularly in the NEA. The objectives of this study were therefore to: (i) examine the realised depth- temperature niche occupied by sharks close to their northern distributional limit across time, (ii) inspect periodicity in vertical movement behaviour across individuals and time, (iii) explore possible drivers of vertical movement behaviour and (iv) highlight implications of vertical occupancy and activity patterns for coastal fisheries management. In doing so, we provide insights into the vertical space use and activity patterns of this economically important and yet enigmatic species.

## Methods

### Tagging data

Subsequent analysis is based on data from female spurdogs, tracked for 86–366 days in four consecutive years (2019–2022) between late October to early December with pop-up archival transmitting tags (PSATs, *MiniPAT-348*, Wildlife Computers, Redmond, WA, United States, n = 21, Table S1). Spurdogs were tagged in different locations along the western Norwegian coast between 60.02°N and 60.52°N to capture movement behaviour across the wider fjord system. In 2019 and 2020, tagging took place south of the city of Bergen, in the Hardangerfjord. In 2021 and 2022, sharks were tagged north of Bergen, in Herdlefjorden which is part of the Osterfjord area (see Figure S1). This area hosts an acoustic receiver array which allowed to confirm year-long residency within the fjord system for sharks double tagged with acoustic tags in 2022 (see Junge et al., in review).

The tagging method and data are described in detail by Junge et al. (*in review*). In short, female sharks between 85 and 115 cm total length were tagged after confirming their pregnancy status via a portable ultrasound (Mindray DP 50 vet). PSATs were tied to a harness made of 1 mm thick braided nylon cord which was attached to two plates, each on either side of the shark. This allowed the tag to trail freely behind the dorsal fin. In the first two years (2019, 2020), the plates were placed slightly posterior to the first dorsal fin, which was optimised by moving them forward in subsequent years to minimise premature tag loss after extending the scheduled deployment period from 180 to 360 days in 2020. In case of suspected mortality (i.e., constant pressure at depth; variance ≤ 2.5 m) or early tag detachment, the tags were programmed to detach and report after two days. Nineteen out of 21 PSATs popped up within the connected fjord system around Bergen within 40km distance from the tagging location (see Figure S1) following premature (n = 6) and scheduled (n = 10) detachment or recapture by fishers (n = 3) (Table S1). Recaptures occurred at nighttime in commercial bottom gillnets between 10 and 100 m depth (Figure S2). A dissection of two recaptured sharks identified an absence of embryos in shark 11 and 17 at the point of recapture in late September and October almost one year after tagging. Until February 2024, two more sharks were reported as recaptures after PSAT detachment, but the fishers did not report the animal IDs. All 19 tags were physically recovered which meant that the full 0.2Hz archive including continuous temperature, depth, light level, and triaxial accelerometer data was available for analysis.

### Data processing

Data processing and analysis was performed in R (Version 4.3.2). Archival PSAT data were visually inspected and cleaned to remove potential tagging and capture effects. While an inspection of the depth time series showed no indication of tagging effects on the diving behaviour, we conservatively removed the first 24 h of each track. We also removed any data indicating surface drifting or constant depth prior to tag release. Due to a sensor failure in the tag of shark 5 and shark 13, the tracks were terminated five days prior to the appearance of any extreme and implausible depth records, resulting in only 116 and 82 days respectively for subsequent analysis.

As a proxy for possibly foraging related activity, bursts in acceleration or fast starts were calculated from triaxial acceleration data similar to Wright et al. [40]. First, the magnitude of acceleration (MA) was calculated as the square root of the sum of squares of the raw acceleration values of each axis ($MA=\sqrt{{X}^{2}+{Y}^{2}+{Z}^{2}}$). Then, fast starts were identified using the 95% percentile of MA values for each individual (i.e., upper 5% of MA). In the absence of empirical MA thresholds at which fast starts linked to feeding events occur in spurdogs, i.e. via video material, the 95% threshold was chosen based on the species known feeding ecology in comparison to thresholds used for other species in the literature [33, 35, 38, 40]. A sensitivity analysis with thresholds of 97% and 99% resulted in no markable differences in the overall patterns (Figure S3). While the low sampling rate translates to a down-sampling of fast starts, which may occur on a sub-second level, the relative signal can be assumed to remain consistent and thus still be used to identify periods where such acceleration bursts occur [35, 38, 40].

For most subsequent analyses, raw archival data were aggregated to the minute and hourly level. Due to non-normal distributions for depth, temperature, and light level, these variables were aggregated using the median, while MA and vertical speed were summarised using the mean. The number of fast starts detected in the acceleration time series were summed per hour. Given evidence for a coastal association of these individuals in the fjord system during the tagging period (Junge et al., in review), data were linked with information for sunset and sunrise as well as nautical dawn and dusk using *RchivalTag* [81] for the coordinate 5.2°E, 60.3°N, which lies in between both tagging locations. Based on these times two (day, night – marked by sunset and sunrise only), and four (dawn, day, dusk and night – marked by nautical dawn, sunrise, nautical dusk, and sunset) diel periods were classified. Visualisations of the data were performed with *ggplot2* [82], and differences between and within groups were visualised with *ggstatsplot* [83, 84].

### Depth-temperature niche

To compute the realised depth-temperature niche of the sharks, kernel density estimation was applied to hourly data using the *MASS* package [85]. The bandwidth.nrd {MASS} function was applied to depth and temperature to calculate a suited smoothing bandwidth for x and y. Similar to standard procedures in horizontal space to estimate home ranges [86], the 95% and 50% isopleths were calculated. To account for the heterogenous data density across different times of the year due to variation in the times at liberty (see Table S1), densities were weighted by the reciprocal sum of entries per Julian day. Realised niches were set into context of the available depth-temperature space using data from Conductivity, Temperature, Depth (CTD) recorder profiles from a hydrographical station in Hardangerfjord (H2 station—60.39°N, 6.34°E) collected nearly every month throughout the deployment period by a RBR Concerto 3—CTD instrument.

### Periodicity in vertical movement

To investigate the periodicity in the depth signal of each tag, a continuous wavelet analysis was performed on hourly depth time series using the *WaveletComp* package [87]. In a wavelet analysis, functions, which are referred to as wavelets, are used to localize specific frequencies as a function of time [88, 89]. Here we used the Morlet (x_0_ = 6) wavelet function, as it is well suited to isolate frequencies with a signal while maintaining a good compromise between both time and frequency resolution [31, 88, 89] and includes a bias correction to prevent high-frequency phenomena from being underestimated [90, 91]. Significance of the wavelet spectrum was assessed by generating 1,000 simulated time series for each individual with a lag-1 autoregressive (AR(1)) model using p = 0.5 and the mean of the data to test the null hypothesis of ‘no periodicity’ while preserving the short-term autocorrelation structure of the original time series [88]. Statistical significance was assessed by comparing the local and global, scale-averaged wavelet power spectra to this distribution. Values exceeding the bootstrapped 95% confidence levels were considered statistically significant and were used to identify non-random vertical migratory behaviour within the time series. For each shark, the wavelet spectrum was calculated for the entire deployment period and prevalent periods in the depth signal were displayed over time in form of a scalogram.

To determine if an individual undertook significant diel vertical migrations (DVM), the global wavelet power spectrum was examined for a significant peak at the 1-day (i.e., 24 h) period. To inspect inter-individual differences in the occurrence of DVM behaviour across time, a cluster analysis was performed on the p-values associated with the 24h-period obtained from the wavelet analysis for each shark. Hourly p-values were averaged per day and a rolling mean with a window of 11 days was applied to smooth over the time series to inspect similarity of larger temporal patterns in the resulting signal. Euclidian distance was used to obtain distance matrices and dendrograms using the *factoextra* package [92]. As entire time series were used, missing values for sharks with shorter than 364d deployments are excluded from all computations involving the rows within which they occur, and sums are scaled up proportionally to the number of columns used.

Differences between groups were tested using appropriate statistical tests. For example, a student’s t-test was used to assess if vertical speeds at different times of day were significantly different from zero. Mann–Whitney U tests were used to compare the number of fast starts during DVM and non-DVM behaviour and Wilcoxon singed rank tests were used for paired and non-parametric data e.g. when comparing median depths during day and night.

### Vertical occupancy and activity hotspots

To identify vertical occupancy and activity hotspots, we binned the 0.2 Hz data into cells of 10 m depth and 1 h for each month and calculated the scaled number of fast starts per depth-time bin. To ensure comparability between months, fast starts were scaled by the number of datapoints of each month. To rule out any possible tagging effects on fast starts beyond 24h after tagging, the analysis was also conducted with the first 120h (5 days) of data post tagging removed.

## Results

The cleaned archival dataset a comprised continuous time series of depth, temperature, light level, and triaxial acceleration at 0.2Hz resolution for 19 female spurdogs over a total of 4,612 days. With an average time at liberty of 243 days (range 82–364d) this dataset is temporally extensive and for at least seven individuals (≥ 319d) allows the analysis of movement behaviour across all seasons (Table S1).

### Depth-temperature niche

**Fig. 1:**
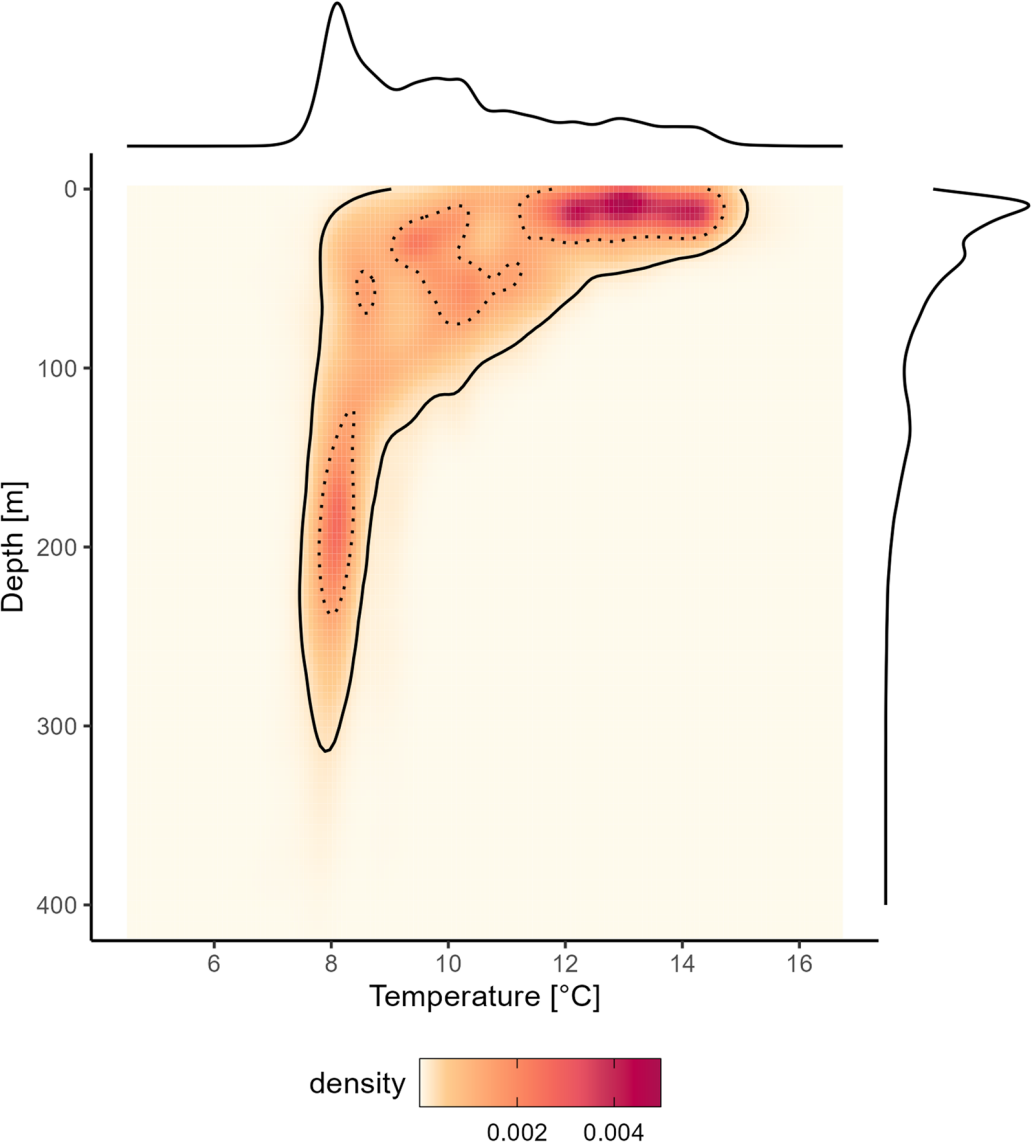
Depth-temperature niche for 19 female spurdogs. Red colours denote the density of hourly data points (n = 110, 360) within a given grid cell weighted by the reciprocal of data points per Julian day. Black dotted and solid lines indicate the niche space that encompasses 50% and 95% of the points, respectively. Marginal densities are shown for both covariates on the upper and righthand side. For visualisation purposes, the y-axis was limited to 400 m depth

Across all 19 individuals and the entire deployment period, sharks used a median depth of 56.5 m (interquartile range (IQR) – 21.5–128.0 m, range 0.0–644.0 m) with a median temperature of 9.6 °C (IQR 8.3–11.1 °C, range 4.5–18.2 °C). Accounting for differences in the coverage in respect to Julian days, most time was spent in waters between 25 and 50 m (21.9% ± 0.5 standard error), with 68.9% of time spent in the upper 75 m (here referred to as “shallow depths”). The sharks spent 90.9% of the time in the epipelagic zone (≤ 200 m; sunlight zone), such that the mesopelagic zone (> 200–1,000 m; twilight zone) was visited only 9% of the time during an average day. Regarding temperature, the most frequented temperature across the deployment was 8–10 °C (37.1 ± 1.0%), followed by 12–14 °C (24.9 ± 1.1%) and 10–12 °C (21.1 ± 0.8%) (Fig. 1, S4).

This encompassed a lot of seasonal variation, with generally deeper and colder waters occupied in boreal winter and spring, and the utilization of warmer, shallower waters in summer and autumn. Daily median depths increased continuously from September (16.3 ± 0.8 m) to January (94.8 ± 1.5 m) and remained between 80 and 86 m until April. Subsequently mean daily depths decreased to 66.4 ± 3.2 m in May and 22.5 ± 3.6 m in June and remained at shallow depths at around 20–40 m until November. On average, daily depth ranges and IQR were particularly extensive in the winter and spring (Dec–May) with ranges around 300 m, and IQRs between 79.9 and 114.3 m whereas in August and September mean daily depth ranges were around 170 m and IQRs include only 16 m (Sep–Oct). Noticeably, mean daily minimum depths do not extend to surface waters (≤ 10 m) from December until March (16.4 ± 1.0 m − 29.8 ± 1.4 m). From July until October, mean daily maximal depth did not extend down to the mesopelagic zone (> 200 m) on average reaching only to 184 m depth. From September to April, selected waters decreased in their mean daily temperatures from 13.9 ± 0.1 °C to 8.4 ± 0.0 °C. From May on, the temperatures in the occupied waters increase until September. The IQR of temperatures, which incorporates variation across all 1-min temperature intervals recorded per date across individuals, was greatest in June, July, August, and November (2.1–2.9 °C). This trend was also apparent in the overall temperature range, which in July for example extended from 8.1 ± 0.1 to 15.1 ± 0.1 °C. The corresponding range in April was only 7.5 ± 0.0 to 9.5 ± 0.0 °C (Figure S5).

Modulated by the season, female spurdogs exhibited a bimodal habitat use, with high occupancy of shallow depths in the first 75 m and elevated temperatures between 10 and 16 °C, as well as deeper waters between 150 and 200 m at rather constant 8 °C (Fig. 1). In winter, cool surface waters (4–7 °C) which are separated from warmer intermediate waters by a strong thermocline at 10–20 m as indicated by H2 CTD profiles, were not occupied (Figure S6). Between December and April, surface waters were visited only 0.0 ± 0.0% to 1.6 ± 0.2% of the time, while waters below 200 m were used between 15.5 ± 1.4% to 20.3 ± 1.3% between January and April. In late summer and early autumn (Aug-Oct), the realised niche was rather unimodal, focussing on warm shallow waters (< 20 m, 12–15 °C) which coincide with the warm fjord-based surface layers above the thermocline. From June to September 30.5 ± 3.9–52.6 ± 3.0% of the time are spent in the first 10 m while in these and the two subsequent months only 1.4 ± 0.4 to 6.2 ± 1.3% was spent in the mesopelagic zone (Figures S7, S8).

### Periodicity in vertical movement

Based on the scale-averaged results from the continuous wavelet analysis, which collapsed the time-domain, a significant period around 24h was detected across all individuals (n = 19). In all but two sharks, a significant 12h period was present. In one individual, a significant period around 14 ± 1d was present; in six individuals this was the case for a 28 ± 2d period. Sixteen sharks showed significant periods greater than 84d (Fig. 3B).

**Fig. 2:**
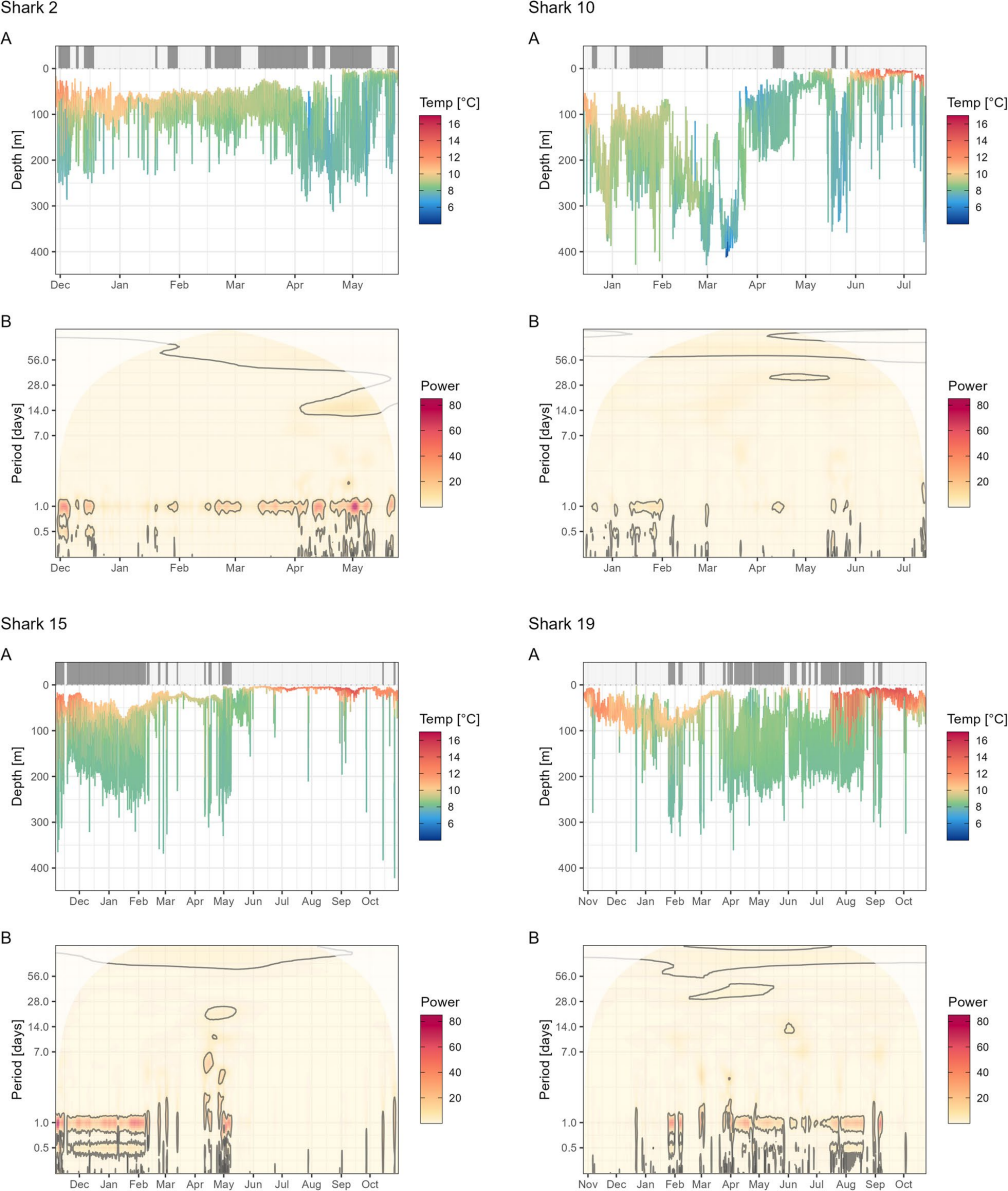
Exemplary hourly median depth time series (**A**) and corresponding wavelet scalogram (**B**). In **B** significant wavelet powers (p ≤ 0.05) are highlighted with grey contours. In **A** the upper bar indicates the presence (dark grey) or absence (light grey) of diel vertical migration (DVM) behaviour, based on significance (p ≤ 0.05) of wavelet powers at 24h

**Fig. 3:**
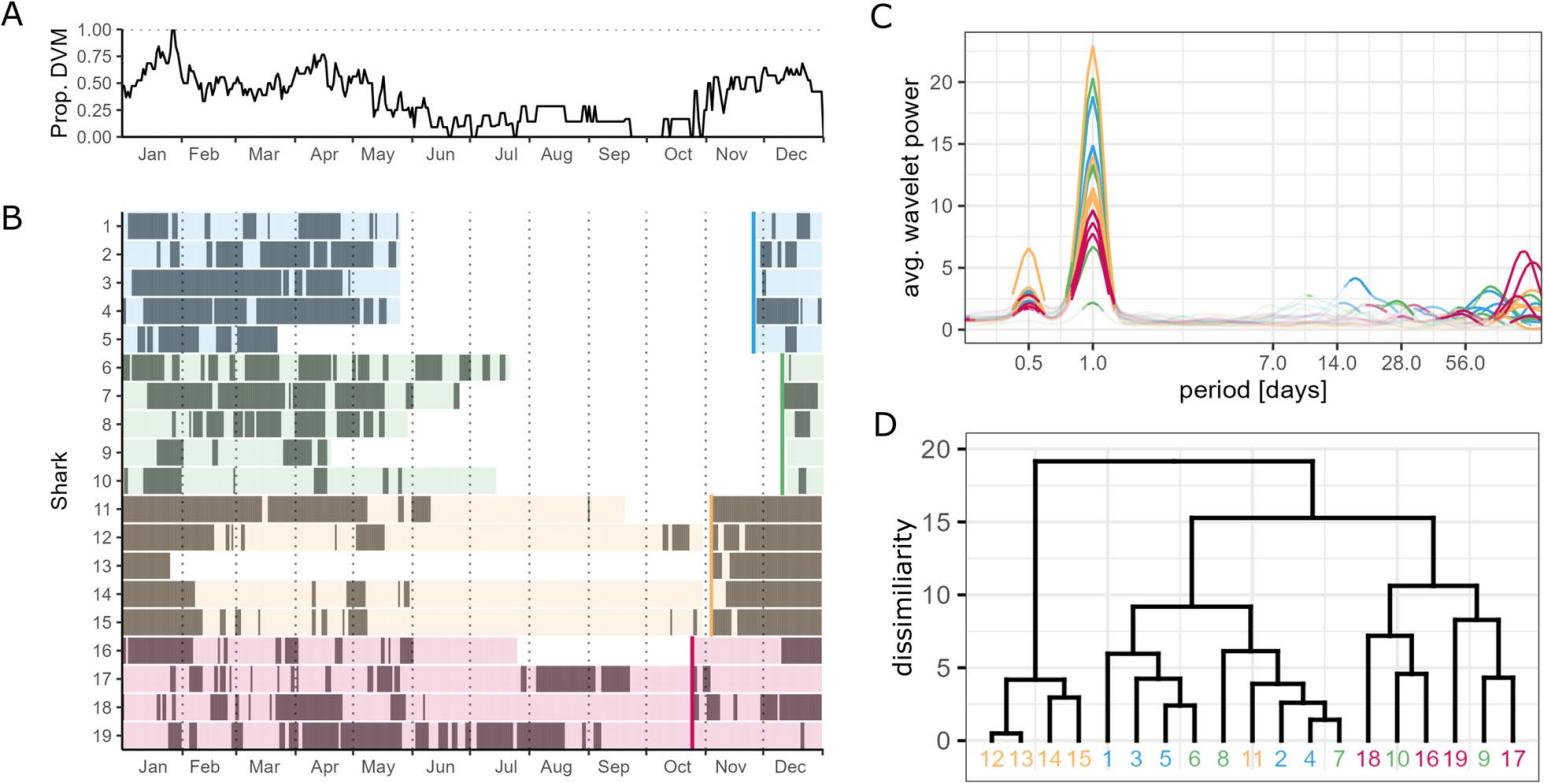
Individual differences in diel vertical migration (DVM) behaviour across a year. Colours indicate tagging years (2019-blue, 2020-green, 2021-yellow, 2022-red). **A** Overall proportion of DVM behaviour displayed on a given Julian day based on B. **B** Occurrence of significant DVM behaviour for each shark by Julian day. Dark bands denote days identified as showing predominantly significant DVM behaviour (based on significant 24h period in wavelet power), while light bands refer to days at which this is not the case. Coloured vertical lines present the Julian day of tagging. Data gaps due to different deployment durations are shown in white. **C** Global, scale-averaged periodogram indicating the overall wavelet power for each shark averaged for the entire deployment period. Only significant (p ≤ 0.05) powers are shown without transparency. **D** Dendrogram indicating the dissimilarity between individuals based on Euclidean distance of p-values for the 24h-period

Continuous wavelet analyses performed per individual allowed for the inspection of the persistence of these periods over time (Fig. 2, S9B). Significant diel patterns, classified as DVM behaviour were detected across seasons. However, DVM behaviour was more persistent in winter and spring (Fig. 3A, B). Most individuals for which deployments covered the summer period (i.e. tagging cohort 2021 and 2022) did not display DVM behaviour between June to October, exceptions being sharks 6, 17, and 19, which displayed more than half the time significant DVM behaviour in June, August, and October respectively. In December, January and April, 60–70% of individuals displayed DVM behaviour more than half of the time. The cluster analysis based on significance levels of the 24h period, highlights the similarity of all but shark 11 in the tagging cohort 2021 and all individuals from 2022. Amongst those, sharks 17 and 19 are different from the others, displaying DVM also in summer. However, one should note that patterns are matched across different deployment durations and years. While overall dendrogram splits broadly match tagging cohort association, DVM patterns vary amongst individuals of the same tagging year resulting in some ‘mixed branches’ (Fig. 3D).

**Fig. 4:**
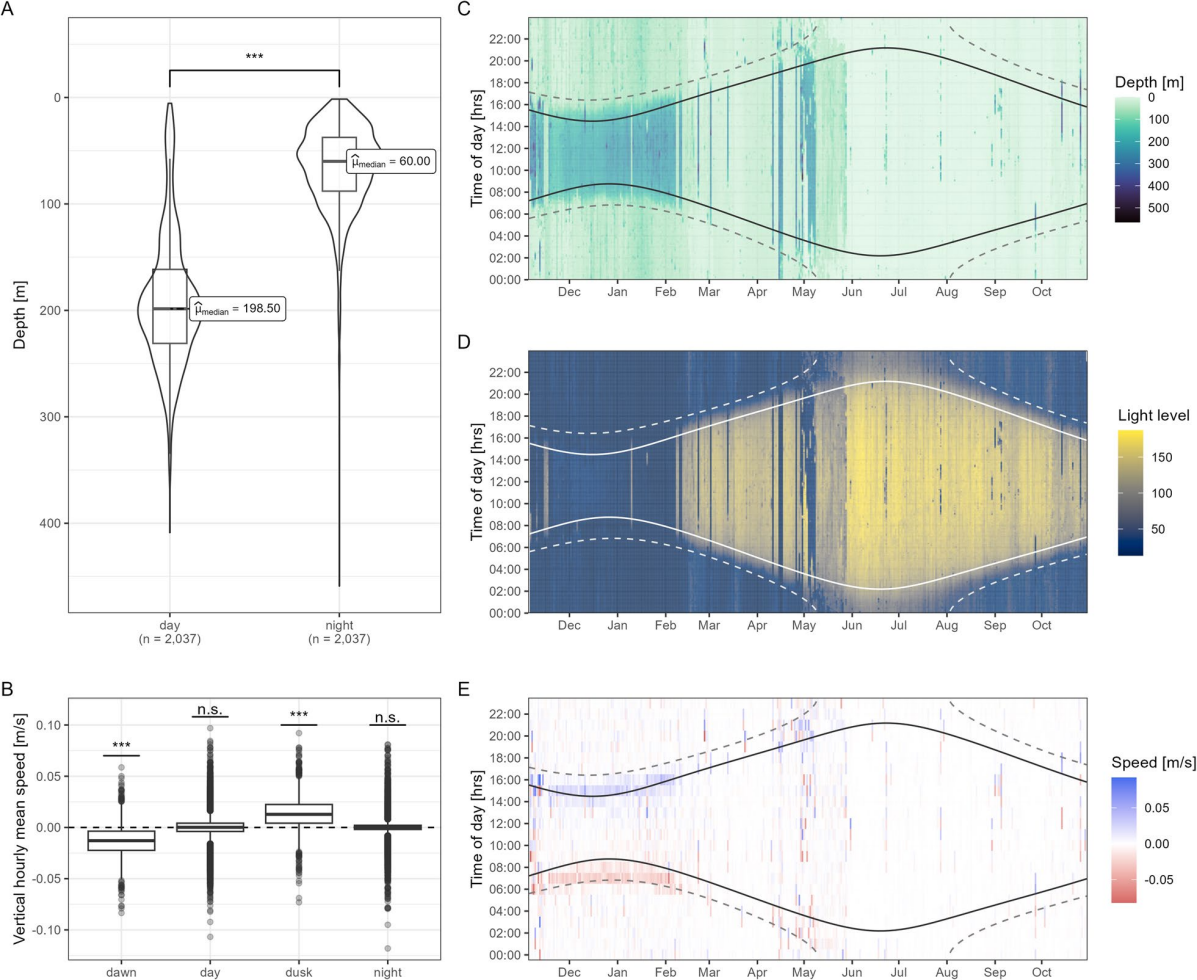
Evidence for normal diel vertical migration (DVM) behaviour during as significant classified DVM. Normal DVM is indicated by (**A**) deeper depth used during the day compared to night and (**B**) negative vertical speeds during dawn and positive speeds during dusk. **C**–**E** highlight depth, light level and vertical speed across the deployment for the time of day exemplary for shark 15. **A** Plot is based on 1-min interval median depth data and boxes indicating the median and the lower and upper quartiles, whiskers are 1.5 times the interquartile range. Violine plots indicate the data range. **B** Plot is based on hourly vertical mean speeds for different daily periods, with boxes showing the mean and outliers shown. Dawn and dusk mark the time between nautical dawn and sunrise as well as dusk and sunset respectively for all sharks. Asterisks denote significance level of Wilcoxon signed rank (**A**) and one-sided t-test (**B**) (***—p ≤ 0.001). One minute interval median depth and light level (**C**,**D**) and hourly mean vertical speeds (**E**) of shark 15 in the context of times of sunrise and sunset (solid lines) and nautical dusk and dawn (sun at 12° below horizon; dashed lines) associated with the tagging location. Light levels of 150, 110, and 70 correlate to 10^–5^, 10^–7^, and 10^−9^ W cm^−2^, respectively (see Supplement Detail S1). In (**E**) blue colours indicate ascends and red colours descents

**Fig. 5:**
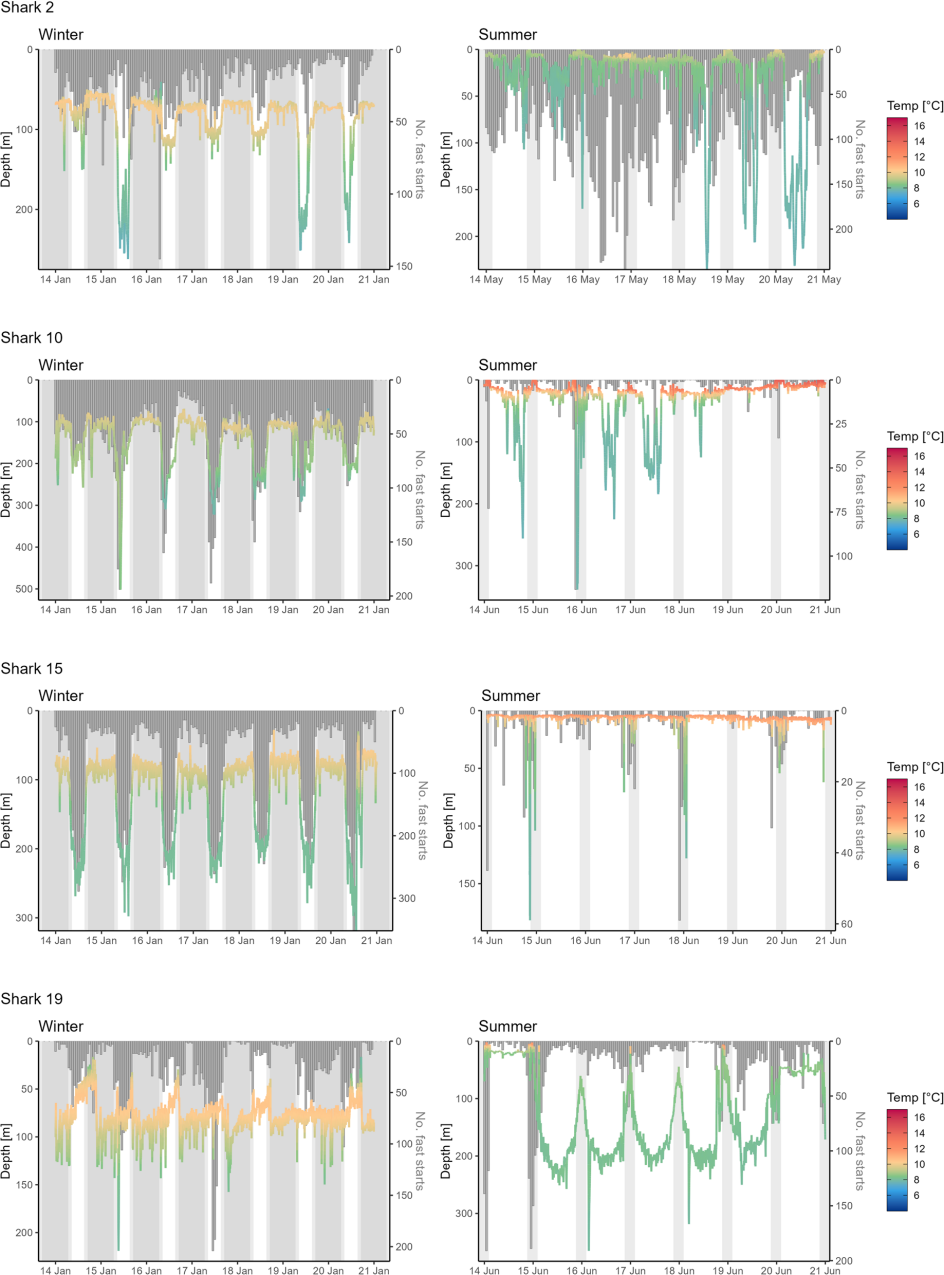
Seasonal differences in depth time series and number of fast starts. Exemplary data shown for sharks 2, 10, 15, and 19 for a week in January (winter, left) and June (early summer, right) based on 1-min interval median depths (left y-axis) and temperatures (as colour) as well as hourly counts of fast starts (dark grey bars, right y-axis). Grey polygons mark night defined by sunset and sunrise and nautical dusk and dawn (sun 12° below horizon, winter only) around the tagging location. Note free y-axes and the x-axis in B showing data for May and not June due to lack of data for later month

Inspection of hourly and minutely time series revealed the DVM pattern overall as strict normal DVM (nDVM) behaviour, with sharks ascending during dusk, remaining at shallower depth during night and descending to deeper depth during dawn, where they remain for the daylight period (Fig. 4, Figure S9, S10). While displaying significant DVM behaviour, median depths at day are significantly deeper (198.5 m, IQR 162–231 m) than at night (60.0 m, IQR 37.5–88 m) (Fig. 4A, *Wilcoxon signed-rank test: V* = *2.06e + 06, n*_*pairs*_ = *2,037, p* < *2.2e*−*16*). This is also supported by mean hourly vertical speeds, which during dusk and dawn were found to be significantly different from zero (*One-sided t-test: t* = − *54.407, df* = *3140, p-value* < *2.2e*−*16; t* = *52.237, df* = *3030, p-value* < *2.2e*−*16*) as opposed to day or night (*One-sided t-test: t* = *0.291, df* = *21,855, p-value* = *0.771; t* = − *0.315, df* = *18,718, p-value* = *0.753*). Vertical speeds were positive at dusk and negative at dawn, marking ascents and descents, respectively (Fig. 4B). Overall, the mean absolute vertical speed was 0.24 m s^−1^. Vertical speeds ranged from − 1.26 to 1.25 m s^−1^. While nDVM behaviour prevails in all individuals across the deployment period (Figure S11), there is indication for periodic reverse DVM (rDVM) behaviour, for example in sharks 18 and 19 in January with shallower depth encountered during the day, particularly during early and late daylight hours as shown in Fig. 5.

### Fast start patterns

The number of hourly fast starts was highest between 200 and 500 m (Figure S12). Fast starts occurred more frequently during DVM behaviour (*one-sided Mann–Whitney test, W* = *1,767,840,970, p-value* < *2.2e* − *16*) (Figure S13). Significantly more fast starts were detected during day than during night, particularly while sharks displayed DVM behaviour (*one-sided Wilcoxon signed rank test, DVM: V* = *1,402,560, p-value* < *2.2e*−*16; non-DVM: V* = *1,588,559, p-value* = *2.689e*−*08,* Figure S14). This is exemplified by sharks 2, 10, and 15 in Fig. 5, which displayed more fast starts in winter during nDVM behaviour, particularly during the day, whereas fewer fast starts were observed in summer. However, there were individual differences in these activity patterns. Based on one-sided Wilcoxon signed rank tests for each individual, four sharks showed no significant difference (shark 10, 13, 14, 17) and four sharks exhibited an opposing trend with an elevated number of fast starts during the night (shark 3, 11, 15, 19) (Figure S15). Within individuals, this could also vary with season, as shown for sharks 15 and 19 (Fig. 5). Comparing hourly fast starts across months (based on sharks for which data for more than 320 days were available i.e. 11, 12, 14, 15, 17, 18, 19) identified November to February, and May as the months with more fast starts than the year-round median (8 fast starts per hour). From August to October, spurdogs showed the lowest fast start activity across all months (median_AUG_ = 1 (IQR 0–15), median_SEP_ = 1 (IQR 0–9), median_OCT_ = 4 (IQR 0–35) fast start per hour) (Figure S16). Correlation of fast starts to environmental conditions revealed a positive correlation with depth (r_pearson_ = 0.836) and a negative correlation with ambient water temperature (r_pearson_ = − 0.686) and light level (r_pearson_ = − 0.794). Lower light levels around 60–70, corresponding to 10^−9^ W cm^−2^ comparable to starlight conditions at the surface, seemed to be associated with the highest median number of fast starts within an hour (Figure S17).

### Vertical occupancy and activity hotspots

**Fig. 6:**
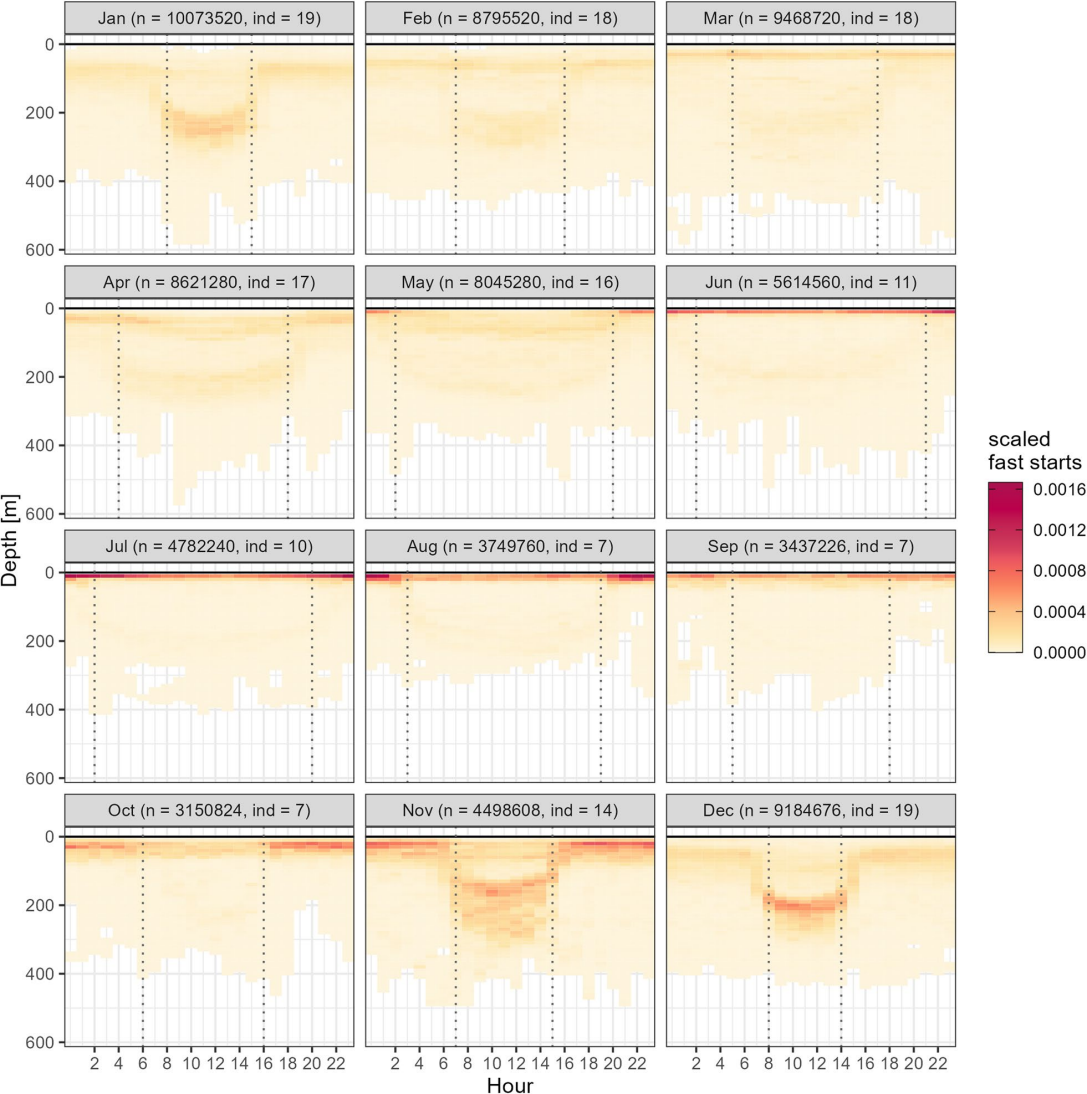
Vertical occupancy and activity hotspots across months calculated as the scaled number of fast starts for 10 m depth bin and each hour of day. The number of data points per month by which data was scaled is noted in brackets together with the number of individuals which contributed to each month. Dotted vertical lines mark the median hour of sunrise and sunset for a given month

Between June and September, female spurdogs showed the highest number of scaled fast starts (at depths from zero to 20 m, particularly during the night, due to the high occupancy in these shallow depths. In October and November, this shallow occupancy and activity hotspot dropped to 20–50 m at night. During the day, this hotspot was prominent between 150 to 250 m (i.e. Nov–Jan), both due to more time spent at this depth and elevated numbers of fast starts in this period (Figure S12). Nighttime hotspots in shallow waters successively ceased over the course of these months. Between February and May, vertical occupancy and activity was less confined, gradually shallowing during the night (Fig. 6). Patterns of elevated fast start activity at depth during October and November remained consistent also when the first five days after tagging were removed from the data (Figure S18).

## Discussion

To our knowledge, this is the first comprehensive analysis of vertical movement of spurdog published to this date. We analysed continuous 0.2 Hz time series data for temperature, depth, light level, and triaxial acceleration from 19 pregnant females over 4,612 days. These data showed a seasonal effect on depth and temperature, in which spurdogs occupied deeper and colder waters during winter and spring, and warmer and shallower waters during summer and autumn. Continuous wavelet analysis revealed a significant 24 h-period in depth use across all individuals. DVM behaviour was most prominent in winter months. Amongst sharks with close to 1-year deployments, most spurdogs did not exhibit DVM behaviour between June and October. A higher occurrence of fast starts was found in association with DVM behaviour, particularly at depths between 150 and 500 m during the day. We identified distinct vertical movement patterns and trends across individuals, season, and time of day to build a better picture of the movement ecology of spurdog at the northern end of their distribution and highlight management implications for this commercially important yet enigmatic species.

### Depth-temperature niche

Biologging can provide consecutive data about the environmental niche occupied by an animal in its natural habitat, allowing inferences about habitat selection on an individual-based level across time and space. The utilised depth and temperature shown in this study align well with other tracking studies from northern latitudes. The depth range of the tagged sharks (0–644 m, median = 56.5 m) is comparable to 0–481.5 m with a mean of 92.6 m as reported by [44], and also falls in the range described in earlier studies [53, 63, 78]. Experienced temperatures (4.5–18.2 °C, median = 9.4 °C) also align well with corresponding DST- or PSAT-based studies with reported ranges of 6.3–15.2 °C (most common 10–11 °C) along the Scottish Westcoast and 2.8–19.2 °C (mean = 9.2 °C) in the northern Gulf of Maine [44, 66].

We found large seasonal variation in the occupied depth-temperature niche with sharks occupying deep and cold waters in boreal winter and spring, while predominantly utilising warm, shallow waters in summer and autumn, a pattern that seems to be consistent with the tendency described for northern subunits in the NWA [44]. As suggested for other Nordic locations [66, 68], we found evidence for temperature-driven and in parts -limited habitat use. In winter, the sharks’ vertical distribution seems to be limited to waters above 6–7 °C, with colder surface waters not being utilised despite the likely nocturnal presence of prey resources [93–98]. Habitat use in summer also seems to be thermally driven, yet likely not thermally limited, with individuals selecting the warmest available water bodies above the shallow thermocline at around 10–15 m depth.

The bimodal depth use in winter and spring results from an oscillatory depth use, which hints to an active use of the water column. Considering that local fjords commonly extend to 600 m and beyond and the sharks’ variable depth use, tagged individuals likely spent a considerable amount of time off the bottom when in these fjords during this period, representative of an active use of the entire available habitat, that is benthic and pelagic. This is in line with recent evidence from tracking studies in the NWA [44, 47] and measured metabolic rates which are above what would be expected if spurdog were associated with a predominantly benthic lifestyle [99]. However, the lack of fine-scale horizontal position data does not permit to link the shark’s depth to the local bathymetry to infer the actual distance of the shark to the seafloor.

### Periodicity in vertical movement

Results from the continuous wavelet analysis revealed this oscillatory pattern in the vertical time series to be predominantly driven by a diel pattern, followed by 12h-periods, which co-occurred with the 24h-patterns. A pattern around 12h is commonly linked to astronomical tidal forcings as here the semi-diurnal lunar constituent (M2) with a period of 12.42 h is the most prominent. Rather than the tidal range itself (max. tidal range 1.0–1.5 m), internal waves in the sill fjords of the study region have the potential to significantly displace sharks and their prey in the water column [100]. The co-occurrence of 12h and 24h patterns and the absence of semi-diurnal cycles when sharks spend substantial time at the pycnocline, where the influence of the interval wave is expected to be highest, however, might indicate that observed 12h cycles may also present harmonics resulting from a non-strictly sinusoidal movement behaviour of the sharks (for simulation results and further discussion see Supplement Detail S2).

Despite anecdotal reports from fishers in the region and evidence for lunar effects on depth use in pelagic predators [101–103], our data did not support an association of depth with lunar cycles in the tracked spurdogs. Across the deployment, only three individuals showed a significant 28 ± 2d period in the wavelet transform. The frequent cloud cover in the study region might dampen a potential lunar illumination effect. The occurrence of significant periods greater than 84 days in more than 80% of individuals underlines seasonal trends to play an important role in the species’ depth use. Given the pronounced, significant diel patterns across individuals, the subsequent section will focus on diel vertical migration (DVM) and respective variations across seasons and individuals.

### Diel vertical migration and its variability

In line with earlier indications from the NWA [44, 47] and previous reports based on fisheries data [50, 104], this study demonstrates spurdogs in the NEA to exhibit diel vertical migration behaviour. As indicated by Carlson et al. [44], diurnal depth differences might be particularly pronounced in Nordic subunits. Across all individuals, a strict nDVM pattern seems to be predominant, with sharks inhabiting consistently deeper waters during daytime and shallow waters during nighttime. This is associated with elevated crepuscular activity in the form of high and significantly positive vertical speeds during dusk (ascents) and significantly negative vertical speeds during dawn (descents). Normal DVM behaviour has been observed in many elasmobranch species such as the starry smooth-hound (*Mustelus asterias*), bluntnose sixgill shark (*Hexanchus griseus*), broadnose sevengill shark (*Notorynchus cepedianus*), reef manta ray (*Mobula alfredi*), blue shark (*Prionace glauca*), porbeagle (*Lamna nasus*), white sharks (*Carcharodon carcharias*) and basking shark (*Cetorhinus maximus*) [3], and has been linked to foraging or behavioural thermo- or oxygen regulation [19, 105].

The extensive temporal range of the dataset, especially for sharks tagged in 2021 and 2022, provided insights into the seasonal variability of DVM patterns. Across individuals it seems that DVM was a dominant pattern over winter and spring (November–May). Yet opposed to our expectations, this pattern dwindled in the summer months, with most sharks utilising almost exclusively the first 25 m of the water column from June to September, with only sporadic dives to deeper depths. Extant individual differences can be linked to cohort association and similarity in the encountered environment as tagging occurred in the same location and within consecutive hours or a few days. With spurdogs being known to move in age- and sex-specific cohorts [50, 51, 58, 62, 76, 77], a synchronisation of DVM patterns might indicate that tagged females in a given year moved around as one cohort for certain time periods. Differences in pop-up locations, however, suggest that this was not the case towards the end of the deployment.

Tagging year rather than tagging location seemed to be a key factor as sharks from cohort 2021 appeared synchronised and distinct in their DVM pattern, compared to cohort 2022, which was tagged in the same location and showed more variation in DVM behaviour. Cohort 2022 seemed more similar to DVM patterns of cohort 2020, although the different temporal extent of the datasets limited the comparison to winter and spring. Given high oceanographic connectivity within the fjord system, this might support the notion that similar environmental conditions result in similar vertical movement patterns in this species. As inter-annual differences in temperatures in this fjord system, particularly at depth, are rather negligible, other factors such as oxygen levels, which change on annual levels, could play a role here. Some individual differences amongst tagging cohorts might be linked to sporadic movements out of the fjord, as we know from pop-up locations and alignment of hydrographic profiles that sharks 3, 9, and 10 must have left the system in winter (Junge et al., in review). Individual differences might also be linked to variations in pregnancy status. While we assumed all individuals to have comparable pregnancy status, it is possible that some females had abortions or earlier parturition, as indicated by sharks 11 and 17 that did not carry any embryos in late September and October. In case of shark 17, this might explain the resumption of DVM behaviour in August, but could also be linked to an abortion, or an early release of the young due to the recapture of the fish, in which case any behaviour prior to recapture should have remained unaffected.

While DVM patterns of most individuals predominantly reflected nDVM, we also found sporadic occurrences of rDVM patterns with shallower depths encountered during the day in some individuals. Such behaviour has been described for basking sharks, porbeagles or blue sharks, while foraging in well-mixed inner-shelf regions where zooplankton may be aggregated in surface waters during the day, with cascading effects on their predators due to tidal fronts [19, 106, 107]. Given the topographically complex fjord system, oceanic features are likely to be patchy on a small scale.

### Possible drivers of vertical movement behaviour

Diel patterns in depth use and movement have frequently been linked to foraging or search behaviour in marine predators, as they have been shown to modify diving behaviour in response to diel-migrating prey [3, 19, 20, 107]. Besides foraging related behaviour, alternative theories to explain diel depth changes include thermo-regulation and bioenergetic efficiency [15, 101]. Yet, other studies have related this behaviour to an affinity to constant light levels possibly to minimise predation risk or maximise foraging success [108–111]. In the following, we focus on foraging and thermoregulation as the main drivers of observed depth use patterns. In the absence of fine-scale horizontal positions, shark-borne oxygen measurements, and telemetry data from other sex and age groups we can only speculate about the role of other possible drivers such as (i) the evasion of hypoxic conditions prevalent in some of the fjord basins, (ii) the minimisation of predation risk for predator naïve offspring, and (iii) the reduction of intra-specific competition via the avoidance other sex- or age groups (see Supplement Detail S3).

### Foraging

Spurdog has been proposed to vertically migrate in pursuit of abundant prey resources that follow their diel migrating planktonic prey [50, 104]. With a standardized trophic level of 3.9, spurdog is an upper secondary-tertiary consumer, commonly found to feed on teleost fish, crustaceans, squid, and ctenophores [78, 112, 113]. In this study, the depths utilised by sharks during DVM behaviour correspond well with the depths of scattering layers of zooplankton [93, 94, 114], as well as (meso)pelagic fish documented in the local fjords in respective seasons (e.g., Mueller's pearlside (*Maurulicus muelleri*) [95, 97, 98, 115, 116], herring (*Clupea harengus*), whiting (*Merlangius merlangus*), Norway pout (*Trisopterus esmarkii*) and sprat (*Sprattus sprattus*) [117–119]. Most of these species have been found in Irish [120] and Norwegian spurdog stomachs (CJ, unpublished data), suggesting sharks follow their prey both during day and night to forage, resulting in observed nDVM patterns. This is in line with previous findings indicating spurdog to feed both during the day and night [77, 78, 104] and to exhibit similar activity levels [67, 121].

To investigate this further, we analysed trends in hourly cumulated fast starts, brief acceleration bursts deemed indicative for the presence of feeding or escaping events [33, 35, 40]. Given the limited presence of known predators for adult female spurdog, such as killer whales (*Orcinus orca*), or grey seals (*Halichoerus grypus*), in the system [104, 122], and evidence for a subordinate influence of predation on diel movements on such a large fish [15, 67], we assumed fast starts to be primarily linked to foraging events. The elevated number of fast starts during DVM behaviour supports the assumption that sharks are displaying DVM to follow their prey. We note, however, that additional data streams, i.e. video records, would add valuable information to link acceleration data and derived metrics such as fast starts to actual feeding behaviour [35, 123]. A comparison of fast starts between day and night suggests most individuals to exhibit higher foraging activity during the day, despite notable individual, and seasonal variation in activity patterns. Unlike demersal small-spotted catsharks (*Scyliorhinus canicula)* or Atlantic stingrays (*Hypanus sabinus*), which were found to forage in warmer surface waters and rest in colder waters at depth to increase nutrient uptake efficiency via reduced evacuation rates [15, 124], these data do not suggest a cessation of feeding activity during specific times of day in the tagged female spurdogs. With fast starts being positively correlated with depth and inversely correlated to temperature, there is no evidence of resting at depth or at cold temperatures but rather for an active lifestyle across both day and night.

Surprisingly, during summer, DVM behaviour ceased in most sharks for which tracks extend to summer and autumn, spending most time in the first 25 m at 12–15 °C above the thermocline with only sporadic dives commonly down to 100–400 m. Evidence from acoustic and trawl surveys as well as bottom-mounted echosounder stations in the local fjords suggests that this is unlikely linked to a behavioural change in prey as DVM of meso-pelagic fish seems to remain persistent across seasons [95, 96, 98, 115, 116, 119]. The low number of fast starts observed during non-DVM behaviour in summer suggests these individuals to have been less active and engaged in possibly foraging related activities, including at night when prey species predate on dense copepod patches closer to the surface [96]. A seasonal prey-shift to prey which is easier to catch and does not result in fast starts might be an alternative explanation but has so far not been reported for spurdog. As an opportunistic feeder, spurdog might exploit highly nutritious food sources during sporadic fast start events involving the chasing of herring and mackerel or extracting of dead fish from aquaculture farms, nets, or bait, as anecdotally reported the region. The 0.2 Hz sampling rate likely translates to a down-sampling of fast starts, so while the relative signal can be assumed to remain consistent, fast starts cannot be translated to an absolute number of high-acceleration events, such as feeding events [35, 40]. Without complementary data such as video footage and seasonally resolved stomach analyses for pregnant females in the fjord system it thus remains open how these sharks meet their likely elevated energy demands in the warmer waters during this period.

### Behavioural thermoregulation

Summer and autumn (Jun-Oct) likely mark the sharks’ second gestation year and final gestation phase. Movement behaviour from this time period suggests that pregnant females de-emphasize foraging and despite possible energetic concessions linked to elevated metabolic rates, select favourable thermal conditions for reproduction [99, 125]. According to the thermal niche-fecundity hypothesis, such thermoregulatory behaviour facilitates egg production and embryonic growth in ectotherms [75, 126, 127]. In Atlantic stingrays, a 1 °C increase in water temperature has been shown to reduce gestation times by up to two weeks [124]. Similar movements of pregnant females to shallower warmer water were also observed in leopard sharks (*Triakis semifasciata*) or round stingrays (*Urobatis halleri*) [128, 129]. In line with earlier findings from high latitudes [72, 74, 130], spurdog data from Oslofjord show embryonic growth during the first and colder half of the second year of pregnancy (Oct-May) to be much slower (0.6cm/month) compared to the second half (May-Dec, 1.2cm/month) which is associated with higher water temperatures [70]. With slight differences in timing possibly due to the high latitude, this would also support earlier hypotheses according to which habitat distribution of adult females in the spring and summer (here summer and autumn) is mainly driven by environmental factors (i.e., temperature and depth), while in the fall (here winter and spring) it is mainly influenced by ecological factors (i.e., prey abundance) [50, 51].

### Implications for coastal fisheries management

In light of recent catch advice for spurdog for the NEA [46] and increasing interactions with gears of fish farms as well as commercial or recreational fishers [45, 133,131−], telemetry data as presented in this study can provide valuable insights for the management of a directed fishery as well as incidental bycatch of spurdog. Availability to fishing gear can be considered a function of where in the fjord system and where in the water column sharks spent their time and where they are active and engaged in searching or foraging behaviour. Based on vertical occupancy and possibly foraging related activity patterns (fast starts), three phases can be distinguished with regards to the availability of late pregnant females to fishing on the Norwegian west coast: (1) June–September: 0–20 m, particularly at night, (2) November–January: 20–50 m during the night and at 150–250 m during the day, (3) February–May: diffuse ‘availability seascape’ across depth and time of day. The active and oscillatory nature of their depth use throughout the water column is likely to expose these female sharks to more variable gear types, compared to less-active species with a more demersal or benthic lifestyle. This is particularly the case for passive gear types, such as gillnets, which rely on active gear encounter. They are commonly used in the coastal fishery on the Norwegian west coast [134], https://www.barentswatch.no/fiskeriaktivitet/) and can be deployed at multiple depths depending on the target species. In fact, 89% of spurdogs are caught as bycatch in nets, predominantly gillnets used in the mixed fishery down to depths of 550 m, often during the summer months [135]. The fact that all recaptured sharks in the present study were caught in commercial bottom gillnets during the tracking period in spring and autumn at depths between 10 and 100 m during nighttime further underlines that bycatch in this gear type is an issue for these pregnant females, which are of elevated management concern due to their key role in stock recruitment.

Identified DVM patterns predominantly found in winter and spring suggest that management measures with regards to bycatch mitigation and the spatio-temporal distribution of a reopened fishery should take such diel and seasonal cycles into account. However, individual variation in depth use and activity patterns observed within this geographically, demographically, and reproductively homogeneous group indicate the need for adaptive management measures. To holistically understand vulnerability to fishing, more horizontal movement data from both males and females and at different age classes will be necessary. High resolution PSAT data here focused on mature females and may in the future be paired with spatial data from an acoustic telemetry array in the region (i.e., ‘Bergen Telemetry Network’) and dedicated surveys such as a new spurdog-specific longline survey [48], both of which incorporate data on other sex- and age-groups. This will help to better assess catchability coefficients of particular gear types for spurdog, which are critical to estimate actual shark densities [136] and quantify bycatch risks in this dynamic system.

## Conclusions

Presented results obtained from the 19 pregnant female spurdogs, satellite-tagged in Norwegian fjords, begin to fill existing knowledge gaps associated with the three-dimensional movement of pregnant spurdog in the NEA. Tagged individuals frequently utilised shallow depths down to 300 m at temperatures between 8–14 °C. Depth use seems to be shaped by diel and seasonal cycles. Normal diel vertical migration patterns are dominating during winter and spring and are likely foraging related given indication for spatio-temporal alignment with local prey patterns and elevated likely feeding-related activity. Variations between individuals and tagging years, however, point to a complex interplay of movement behaviour and habitat use with the abiotic and biotic environment. DVM behaviour is rarely displayed in summer and autumn. During this period, sharks predominantly reside in warm waters above the thermocline with only sporadic dives to 100–400 m depth. The low number of fast starts during this period suggests habitat use rather to be driven by behavioural thermoregulation, yet other factors could also play a role. These results provide critical information for informing the spatio-temporal management of spurdog in the context of a newly reopened fishery in the NEA and increasing bycatch-related conflicts with fishers and fish farmers. Nevertheless, further studies investigating the fine-scale horizontal movement, thermal- and oxygen-preferences, as well as sex- and age-group specific differences in habitat use and foraging habits are needed to build a more comprehensive picture of the mechanisms underlying habitat selection and niche segregation in spurdog in the NEA and beyond.

## Supplementary Information

Additional file 1.

## Data Availability

A detailed description of the tagging method and the dataset can be found in Junge et al. (in review). The code used for the analysis conducted in this study is available on https://github.com/cakloecker/spurdog-psat-analysis (Version 1.0).

## Abbreviations

PSATs: Pop-up archival transmitting tags or pop-up satellite tags
DVM: Diel Vertical Migration
nDVM: Normal Diel Vertical Migration
rDVM: Reverse Diel Vertical Migration
MA: Magnitude of Acceleration
NEA: North-East Atlantic
NWA: North-West Atlantic

## References

[1] Hammerschlag N, Schmitz OJ, Flecker AS, Lafferty KD, Sih A, Atwood TB, Gallagher AJ, Irschick DJ, Skubel R, Cooke SJ. Ecosystem function and services of aquatic predators in the anthropocene. Trends Ecol Evol. 2019;34:369–83. 10.1016/j.tree.2019.01.005. 30857757 10.1016/j.tree.2019.01.005

[2] Nathan R, Getz WM, Revilla E, Holyoak M, Kadmon R, Saltz D, Smouse PE. A movement ecology paradigm for unifying organismal movement research. Proc Natl Acad Sci. 2008;105:19052–9. 10.1073/pnas.0800375105. 19060196 10.1073/pnas.0800375105 PMC2614714

[3] Andrzejaczek S, Lucas TCD, Goodman MC, Hussey NE, Armstrong AJ, Carlisle A, Coffey DM, Gleiss AC, Huveneers C, Jacoby DMP, Meekan MG, Mourier J, Peel LR, Abrantes K, Afonso AS, Ajemian MJ, Anderson BN, Anderson SD, Araujo G, Armstrong AO, Bach P, Barnett A, Bennett MB, Bezerra NA, Bonfil R, Boustany AM, Bowlby HD, Branco I, Braun CD, Brooks EJ, Brown J, Burke PJ, Butcher P, Castleton M, Chapple TK, Chateau O, Clarke M, Coelho R, Cortes E, Couturier LIE, Cowley PD, Croll DA, Cuevas JM, Curtis TH, Dagorn L, Dale JJ, Daly R, Dewar H, Doherty PD, Domingo A, Dove ADM, Drew M, Dudgeon CL, Duffy CAJ, Elliott RG, Ellis JR, Erdmann MV, Farrugia TJ, Ferreira LC, Ferretti F, Filmalter JD, Finucci B, Fischer C, Fitzpatrick R, Forget F, Forsberg K, Francis MP, Franks BR, Gallagher AJ, Galvan-Magana F, García ML, Gaston TF, Gillanders BM, Gollock MJ, Green JR, Green S, Griffiths CA, Hammerschlag N, Hasan A, Hawkes LA, Hazin F, Heard M, Hearn A, Hedges KJ, Henderson SM, Holdsworth J, Holland KN, Howey LA, Hueter RE, Humphries NE, Hutchinson M, Jaine FRA, Jorgensen SJ, Kanive PE, Labaja J, Lana FO, Lassauce H, Lipscombe RS, Llewellyn F, Macena BCL, Mambrasar R, McAllister JD, McCully Phillips SR, McGregor F, McMillan MN, McNaughton LM, Mendonça SA, Meyer CG, Meyers M, Mohan JA, Montgomery JC, Mucientes G, Musyl MK, Nasby-Lucas N, Natanson LJ, O’Sullivan JB, Oliveira P, Papastamtiou YP, Patterson TA, Pierce SJ, Queiroz N, Radford CA, Richardson AJ, Richardson AJ, Righton D, Rohner CA, Royer MA, Saunders RA, Schaber M, Schallert RJ, Scholl MC, Seitz AC, Semmens JM, Setyawan E, Shea BD, Shidqi RA, Shillinger GL, Shipley ON, Shivji MS, Sianipar AB, Silva JF, Sims DW, Skomal GB, Sousa LL, Southall EJ, Spaet JLY, Stehfest KM, Stevens G, Stewart JD, Sulikowski JA, Syakurachman I, Thorrold SR, Thums M, Tickler D, Tolloti MT, Townsend KA, Travassos P, Tyminski JP, Vaudo JJ, Veras D, Wantiez L, Weber SB, Wells RJD, Weng KC, Wetherbee BM, Williamson JE, Witt MJ, Wright S, Zilliacus K, Block BA, Curnick DJ. Diving into the vertical dimension of elasmobranch movement ecology. Sci Adv. 2022;8:eabo1754. 10.1126/sciadv.abo1754. 35984887 10.1126/sciadv.abo1754 PMC9390984

[4] Arostegui MC, Muhling B, Culhane E, Dewar H, Koch SS, Braun CD. A shallow scattering layer structures the energy seascape of an open ocean predator. Sci Adv. 2023;9:eadi8200. 10.1126/sciadv.adi8200. 37792940 10.1126/sciadv.adi8200 PMC10550225

[5] Braun CD, Arostegui MC, Thorrold SR, Papastamatiou YP, Gaube P, Fontes J, Afonso P. The functional and ecological significance of deep diving by large marine predators. Annu Rev Mar Sci. 2022;14:129–59. 10.1146/annurev-marine-032521-103517. 10.1146/annurev-marine-032521-103517 34416123

[6] Braun CD, Della Penna A, Arostegui MC, Afonso P, Berumen ML, Block BA, Brown CA, Fontes J, Furtado M, Gallagher AJ, Gaube P, Golet WJ, Kneebone J, Macena BCL, Mucientes G, Orbesen ES, Queiroz N, Shea BD, Schratwieser J, Sims DW, Skomal GB, Snodgrass D, Thorrold SR. Linking vertical movements of large pelagic predators with distribution patterns of biomass in the open ocean. Proc Natl Acad Sci. 2023;120: e2306357120. 10.1073/pnas.2306357120. 38150462 10.1073/pnas.2306357120 PMC10666118

[7] Levin N, Kark S, Danovaro R. Adding the third dimension to marine conservation. Conserv Lett. 2018;

[8] Vedor M, Queiroz N, Mucientes G, Couto A, da Costa I, dos Santos A, Vandeperre F, Fontes J, Afonso P, Rosa R, Humphries NE, Sims DW. Climate-driven deoxygenation elevates fishing vulnerability for the ocean’s widest ranging shark. Elife. 2021;10: e62508. 10.7554/eLife.62508. 33461659 10.7554/eLife.62508 PMC7815312

[9] Andrzejaczek S, Gleiss AC, Pattiaratchi CB, Meekan MG. Patterns and drivers of vertical movements of the large fishes of the epipelagic. Rev Fish Biol Fish. 2019;29:335–54. 10.1007/s11160-019-09555-1. 10.1007/s11160-019-09555-1

[10] Bernal D. An Introduction to the Biology of Pelagic Fishes. In: Encyclopedia of Fish Physiology. Amsterdam: Elsevier; 2011.

[11] Carey FG, Scharold JV, Kalmijn AdJ. Movements of blue sharks (*Prionace glauca*) in depth and course. Mar Biol. 1990;106:329–42. 10.1007/BF01344309. 10.1007/BF01344309

[12] Gleiss AC, Schallert RJ, Dale JJ, Wilson SG, Block BA. Direct measurement of swimming and diving kinematics of giant Atlantic bluefin tuna (*Thunnus thynnus*). R Soc Open Sci. 2019;6:190203. 10.1098/rsos.190203. 31218059 10.1098/rsos.190203 PMC6549966

[13] Gleiss AC, Wright S, Liebsch N, Wilson RP, Norman B. Contrasting diel patterns in vertical movement and locomotor activity of whale sharks at Ningaloo Reef. Mar Biol. 2013;160:2981–92. 10.1007/s00227-013-2288-3. 10.1007/s00227-013-2288-3

[14] Peter Klimley A, Beavers SC, Curtis TH, Jorgensen SJ. Movements and swimming behavior of three species of sharks in La Jolla Canyon, California. Environ Biol Fishes. 2002;63:117–35. 10.1023/A:1014200301213. 10.1023/A:1014200301213

[15] Sims DW, Wearmouth VJ, Southall EJ, Hill JM, Moore P, Rawlinson K, Hutchinson N, Budd GC, Righton D, Metcalfe JD, Nash JP, Morritt D. Hunt warm, rest cool: bioenergetic strategy underlying diel vertical migration of a benthic shark. J Anim Ecol. 2006;75:176–90. 10.1111/j.1365-2656.2005.01033.x. 16903055 10.1111/j.1365-2656.2005.01033.x

[16] Cohen J, Forward R. Zooplankton diel vertical migration—a review of proximate control. In: Aaa A, editor. Oceanography and marine biology, annual review. Boca Raton: CRC Press; 2009. p. 77–109. 10.1201/9781420094220.ch2.

[17] Tarling G, Jarvis T, Matthews J. Calanus finmarchicus descends in response to the arrival of krill—better unfed than dead. Mar Ecol Prog Ser. 2003;252:307–10. 10.3354/meps252307. 10.3354/meps252307

[18] Thygesen UH, Patterson TA. Oceanic diel vertical migrations arising from a predator-prey game. Theor Ecol. 2019;12:17–29. 10.1007/s12080-018-0385-0. 10.1007/s12080-018-0385-0

[19] Sims DW, Southall EJ, Tarling GA, Metcalfe JD. Habitat-specific normal and reverse diel vertical migration in the plankton-feeding basking shark. J Anim Ecol. 2005;74:755–61. 10.1111/j.1365-2656.2005.00971.x

[20] Schaefer KM, Fuller DW, Block BA. Vertical movements and habitat utilization of skipjack (*Katsuwonus pelamis*), yellowfin (*Thunnus albacares*), and bigeye (*Thunnus obesus*) tunas in the equatorial eastern pacific ocean, ascertained through archival tag data. In: Nielsen JL, Arrizabalaga H, Fragoso N, Hobday A, Lutcavage M, Sibert J, editors. Tagging and tracking of marine animals with electronic devices, reviews: methods and technologies in fish biology and fisheries. Dordrecht: Springer; 2009. p. 121–44. 10.1007/978-1-4020-9640-2_8.

[21] Hussey NE, Kessel ST, Aarestrup K, Cooke SJ, Cowley PD, Fisk AT, Harcourt RG, Holland KN, Iverson SJ, Kocik JF, Mills Flemming JE, Whoriskey FG. Aquatic animal telemetry: a panoramic window into the underwater world. Science. 2015;348:1255642. 10.1126/science.1255642. 26068859 10.1126/science.1255642

[22] Kays R, Crofoot MC, Jetz W, Wikelski M. Terrestrial animal tracking as an eye on life and planet. Science. 2015;348:aaa2478. 10.1126/science.aaa2478. 26068858 10.1126/science.aaa2478

[23] Watanabe YY, Papastamatiou YP. Biologging and biotelemetry: tools for understanding the lives and environments of marine animals. Annu Rev Anim Biosci. 2023;11:247–67. 10.1146/annurev-animal-050322-073657. 36790885 10.1146/annurev-animal-050322-073657

[24] Soberón J, Nakamura M. Niches and distributional areas: concepts, methods, and assumptions. Proc Natl Acad Sci. 2009;106:19644–50. 10.1073/pnas.0901637106. 19805041 10.1073/pnas.0901637106 PMC2780935

[25] Vihtakari M, Hordoir R, Treble M, Bryan MD, Elvarsson B, Nogueira A, Hallfredsson EH, Christiansen JS, Albert OT. Pan-Arctic suitable habitat model for Greenland halibut. ICES J Mar Sci. 2021;78:1340–56. 10.1093/icesjms/fsab007. 10.1093/icesjms/fsab007

[26] Graham RT, Roberts CM, Smart JCR. Diving behaviour of whale sharks in relation to a predictable food pulse. J R Soc Interface. 2005. 10.1098/rsif.2005.0082. 16849222 10.1098/rsif.2005.0082 PMC1618489

[27] Shepard E, Ahmed M, Southall E, Witt M, Metcalfe J, Sims D. Diel and tidal rhythms in diving behaviour of pelagic sharks identified by signal processing of archival tagging data. Mar Ecol Prog Ser. 2006;328:205–13. 10.3354/meps328205. 10.3354/meps328205

[28] Speed C, Meekan M, Field I, McMahon C, Stevens J, McGregor F, Huveneers C, Berger Y, Bradshaw C. Spatial and temporal movement patterns of a multi-species coastal reef shark aggregation. Mar Ecol Prog Ser. 2011;429:261–75. 10.3354/meps09080. 10.3354/meps09080

[29] Tolotti M, Bauer R, Forget F, Bach P, Dagorn L, Travassos P. Fine-scale vertical movements of oceanic whitetip sharks (*Carcharhinus longimanus*). Fish Bull. 2017;115:380. 10.7755/Fb.115.3.8. 10.7755/Fb.115.3.8

[30] Burke PJ, Mourier J, Gaston TF, Williamson JE. Novel use of pop-up satellite archival telemetry in sawsharks: insights into the movement of the common sawshark *Pristiophorus cirratus* (Pristiophoridae). Anim Biotelem. 2020;8:33. 10.1186/s40317-020-00222-y. 10.1186/s40317-020-00222-y

[31] Goetz FW, Jasonowicz AJ, Roberts SB. What goes up must come down: diel vertical migration in the deep-water sablefish (*Anoplopoma fimbria*) revealed by pop-up satellite archival tags. Fish Oceanogr. 2018;27:127–42. 10.1111/fog.12239. 10.1111/fog.12239

[32] Stehfest K, Patterson T, Barnett A, Semmens J. Intraspecific differences in movement, dive behavior and vertical habitat preferences of a key marine apex predator. Mar Ecol Prog Ser. 2014;495:249–62. 10.3354/meps10563. 10.3354/meps10563

[33] Domenici P, Blake RW. The kinematics and performance of fish fast-start swimming. J Exp Biol. 1997;200:1165–78. 10.1242/jeb.200.8.1165. 9319004 10.1242/jeb.200.8.1165

[34] Rice A, Hale ME. The role of locomotion in feeding performance of fishes. In: Fish locomotion: an ethoecological approach. Boca Raton: CRC Press; 2010. p. 171–99.

[35] Broell F, Noda T, Wright S, Domenici P, Steffensen JF, Auclair J-P, Taggart CT. Accelerometer tags: detecting and identifying activities in fish and the effect of sampling frequency. J Exp Biol. 2013;216:1255–64. 10.1242/jeb.077396. 23197088 10.1242/jeb.077396

[36] Broell F, Taylor AD, Litvak MK, Bezanson A, Taggart CT. Post-tagging behaviour and habitat use in shortnose sturgeon measured with high-frequency accelerometer and PSATs. Anim Biotelem. 2016;4:11. 10.1186/s40317-016-0103-x. 10.1186/s40317-016-0103-x

[37] Føre M, Alfredsen JA, Gronningsater A. Development of two telemetry-based systems for monitoring the feeding behaviour of Atlantic salmon (*Salmo salar* L.) in aquaculture sea-cages. Comput Electron Agric. 2011;76:240–51. 10.1016/j.compag.2011.02.003. 10.1016/j.compag.2011.02.003

[38] Gandra M, Winkler AC, Afonso P, Abecasis D. Long-distance migrations and seasonal movements of meagre (*Argyrosomus regius*), a large coastal predator, along the Iberian Peninsula coast. Mov Ecol. 2024;12:35. 10.1186/s40462-024-00469-7. 38725044 10.1186/s40462-024-00469-7 PMC11080147

[39] Noda T, Kawabata Y, Arai N, Mitamura H, Watanabe S. Animal-mounted gyroscope/accelerometer/magnetometer: In situ measurement of the movement performance of fast-start behaviour in fish. J Exp Mar Biol Ecol. 2014;451:55–68. 10.1016/j.jembe.2013.10.031. 10.1016/j.jembe.2013.10.031

[40] Wright SR, Righton D, Naulaerts J, Schallert RJ, Griffiths CA, Chapple T, Madigan D, Laptikhovsky V, Bendall V, Hobbs R, Beare D, Clingham E, Block B, Collins MA. Yellowfin tuna behavioural ecology and catchability in the South Atlantic: the right place at the right time (and depth). Front Mar Sci. 2021;8:664593. 10.3389/fmars.2021.664593 10.3389/fmars.2021.664593

[41] Hammerschlag N, Gallagher AJ, Lazarre DM. A review of shark satellite tagging studies. J Exp Mar Biol Ecol. 2011;398:1–8. 10.1016/j.jembe.2010.12.012. 10.1016/j.jembe.2010.12.012

[42] Renshaw S, Hammerschlag N, Gallagher AJ, Lubitz N, Sims DW. Global tracking of shark movements, behaviour and ecology: a review of the renaissance years of satellite tagging studies, 2010–2020. J Exp Mar Biol Ecol. 2023;560: 151841. 10.1016/j.jembe.2022.151841. 10.1016/j.jembe.2022.151841

[43] Albert OT, Junge C, Myrlund MK. Young mums are rebuilding the spurdog stock (*Squalus acanthias* L.) in Norwegian waters. ICES J Mar Sci. 2019;76:2193–204. 10.1093/icesjms/fsz156. 10.1093/icesjms/fsz156

[44] Carlson AE, Hoffmayer ER, Tribuzio CA, Sulikowski JA. The use of satellite tags to redefine movement patterns of spiny dogfish (*Squalus acanthias*) along the U.S. east coast: implications for fisheries management. PLoS ONE. 2014;9:0103384. 10.1371/journal.pone.0103384. 10.1371/journal.pone.0103384 PMC4113362 25068584

[45] ICES. Report of the Working Group on Elasmobranch Fishes (WGEF) (report). ICES Sci Rep. 2023;5:92. 10.17895/ices.pub.24190332.v1 10.17895/ices.pub.24190332.v1

[46] ICES. Spurdog (*Squalus acanthias*) in subareas 1–10, 12, and 14 (the Northeast Atlantic and adjacent waters) (report). ICES Advice Recurr Advice. 2022. 10.17895/ices.advice.19753588.v1 10.17895/ices.advice.19753588.v1

[47] Sulikowski J, Galuardi B, Bubley W, Furey N, Driggers W, Ingram G, Tsang P. Use of satellite tags to reveal the movements of spiny dogfish *Squalus acanthias* in the western North Atlantic Ocean. Mar Ecol Prog Ser. 2010;418:249–54. 10.3354/meps08821. 10.3354/meps08821

[48] Andrade H, Nilsen T, Vollen T, Harbitz A, Junge C, Albert OT. A longline survey for spurdog distribution and life history along the Norwegian coast. Fish Manag Ecol. 2023. 10.1111/fme.12676. 10.1111/fme.12676

[49] Compagno LJV. FAO species catalogue. Sharks of the world. An annotated and illustrated catalogue of shark species known to date. Part 1—Hexanchiformes to Lamniformes. (No. Vol. 4, Pt. 1), FAO Fisheries Synopsis. Rome: Food and Agriculture Organization of the United Nations; 1984

[50] Sagarese SR, Frisk MG, Cerrato RM, Sosebee KA, Musick JA, Rago PJ. Application of generalized additive models to examine ontogenetic and seasonal distributions of spiny dogfish (*Squalus acanthias*) in the Northeast (US) shelf large marine ecosystem. Can J Fish Aquat Sci. 2014;71:847–77. 10.1139/cjfas-2013-0342. 10.1139/cjfas-2013-0342

[51] Sagarese SR, Frisk MG, Miller TJ, Sosebee KA, Musick JA, Rago PJ. Influence of environmental, spatial, and ontogenetic variables on habitat selection and management of spiny dogfish in the Northeast (US) shelf large marine ecosystem. Can J Fish Aquat Sci. 2014;71:567–80. 10.1139/cjfas-2013-0259. 10.1139/cjfas-2013-0259

[52] McEachran JD, Branstetter S. Squalidae. In: Fishes of the North-Eastern Atlantic and the Mediterranean 1. Paris: UNESCO; 1984. p. 128–47.

[53] Campana SE, Gibson AJF, Marks L, Joyce W, Rulifson R. Stock structure, life history, fishery and abundance indices for spiny dogfish (*Squalus acanthias*) in Atlantic Canada (Canadian Science Advisory Secretariat No. Research Document 2007/089); 2007.

[54] Ford E. A contribution to our knowledge of the life-histories of the dogfishes landed at Plymouth. J Mar Biol Assoc U K. 1921;12:468–505. 10.1017/S0025315400006317. 10.1017/S0025315400006317

[55] Gauld JA, MacDonald WS. The results of tagging experiments on spurdogs, *Squalus acanthias* L., around Scotland. ICES J Mar Sci Pelagic Fish Comm H. 1982;51.

[56] Holden MJ. The stocks of spurdogs (*Squalus acanthias* L.) in British waters, and their migrations, 2. London: Fisheries Investigations; 1965.

[57] McFarlane GA, King JR. Migration patterns of spiny dogfish (*Squalus acanthias*) in the North Pacific Ocean. Fish Bull. 2003;101:358–67.

[58] Pawson MG. Biogeographical identification of English Channel fish and shellfish stocks (Fisheries research technical report No. 99). Ministry of agriculture, fisheries and food; Directorate of Fisheries Research, Lowestoft; 1995.

[59] Rulifson RA. Tagging spiny dogfish overwintering in North Carolina, and summering in Bay of Fundy, Canada. In: Proceedings of the Transboundary Resources Assessment Committee (TRAC) Spiny Dogfish Review, Proceedings. Woods Hole Laboratory Woods Hole, Massachusetts, USA; 2010.

[60] Templeman W. Transatlantic migrations of spiny dogfish (*Squalus acanthias*). J Fish Res Board Can. 1976;33:2605–9. 10.1139/f76-308. 10.1139/f76-308

[61] Vince MR. Stock identity in spurdog (*Squalus acanthias* L.) around the British Isles. Fish Res. 1991;12:341–54. 10.1016/0165-7836(91)90017-A. 10.1016/0165-7836(91)90017-A

[62] Dell’Apa A, Maria Grazia P, Bonzek C. Modeling the habitat distribution of spiny dogfish (*Squalus acanthias*), by sex, in coastal waters of the northeastern United States. Fish Bull. 2016;115:89–100. 10.7755/FB.115.1.8. 10.7755/FB.115.1.8

[63] Dunn MR, Stevens DW, Forman JS, Connell A. Trophic interactions and distribution of some squaliforme sharks, including new diet descriptions for *Deania calcea* and *Squalus acanthias*. PLoS ONE. 2013;8: e59938. 10.1371/journal.pone.0059938. 23536896 10.1371/journal.pone.0059938 PMC3607562

[64] Jac R, Höffle H, Albretsen J, Jakobsdóttir K, Staby A, Søvik G, Junge C. Of three sharks and one chimaera: varied habitat preferences across a latitudinal range revealed by coastal and offshore surveys. J Fish Biol. 2021. 10.1111/jfb.14979. 10.1111/jfb.14979 34931705

[65] Hammerschlag N, Skubel R, Calich H, Nelson E, Shiffman D, Wester J, Macdonald C, Cain S, Jennings L, Enchelmaier A, Gallagher A. Nocturnal and crepuscular behavior in elasmobranchs: a review of movement, habitat use, foraging, and reproduction in the dark. Bull Mar Sci. 2017;93:355–74.

[66] Thorburn J, Neat F, Bailey DM, Noble LR, Jones CS. Winter residency and site association in the Critically Endangered North East Atlantic spurdog *Squalus acanthias*. Mar Ecol Prog Ser. 2015;526:113–24. 10.3354/meps11210. 10.3354/meps11210

[67] Juby R, Bernard A, Götz A. Day/night patterns of habitat use by dogfish sharks (Squalidae) at photic and subphotic warm-temperate reefs: evidence for diel movements and size- and sex-segregation. Afr J Mar Sci. 2021;43:325–36. 10.2989/1814232X.2021.1951839. 10.2989/1814232X.2021.1951839

[68] Shepherd T, Page F, Macdonald B. Length and sex-specific associations between spiny dogfish (*Squalus acanthias*) and hydrographic variables in the Bay of Fundy and Scotian Shelf. Fish Oceanogr. 2002;11:78–89. 10.1046/j.1365-2419.2002.00191.x. 10.1046/j.1365-2419.2002.00191.x

[69] Burgess GH. Spiny dogfish *Squalus acanthias* Linnaeus 1758. In: Bigelow and Schroeder’s fishes of the gulf of Maine. Washington, D.C.: Smithsonian Institution Press; 2002. p. 54–7.

[70] Jones TS, Ugland KI. Reproduction of female spiny dogfish, *Squalus acanthias*, in the Oslofjord. Fish Bull. 2001;99:685–90.

[71] Ketchen KS. Size at maturity, fecundity, and embryonic growth of the spiny dogfish (*Squalus acanthias*) in British Columbia waters. J Fish Res Board Can. 1972;29:1717–23. 10.1139/f72-272. 10.1139/f72-272

[72] Nammack MF, Musick JA, Colvocoresses JA. Life history of spiny dogfish off the Northeastern United States. Trans Am Fish Soc. 1985;114:367–76. 10.1577/1548-8659(1985)114%3c367:LHOSDO%3e2.0.CO;2. 10.1577/1548-8659(1985)114<367:LHOSDO>2.0.CO;2

[73] Stenberg C. Life History of the Piked Dogfish (*Squalus acanthias* L.) in Swedish Waters 35;2005.

[74] Templeman W. The life-history of the spiny dogfish (*Squalus acanthias*): and the vitamin A values of dogfish liver oil (Fish Res Bull). Nfld Gov Dep Nat Resour. 1944.

[75] Dell’Apa A, Cudney-Burch J, Kimmel DG, Rulifson RA. Sexual segregation of spiny dogfish in fishery-dependent surveys in cape cod, massachusetts: potential management benefits. Trans Am Fish Soc. 2014;143:833–44. 10.1080/00028487.2013.869257. 10.1080/00028487.2013.869257

[76] Haugen JB, Curtis TH, Fernandes PG, Sosebee KA, Rago PJ. Sexual segregation of spiny dogfish (*Squalus acanthias*) off the northeastern United States: Implications for a male-directed fishery. Fish Res. 2017;193:121–8. 10.1016/j.fishres.2017.04.007. 10.1016/j.fishres.2017.04.007

[77] Koen Alonso M, Alberto Crespo E, Aníbal García N, Noemí Pedraza S, Ariel Mariotti P, Judith Mora N. Fishery and ontogenetic driven changes in the diet of the spiny dogfish, *Squalus acanthias*, in patagonian waters. Argentina Environ Biol Fishes. 2002;63:193–202. 10.1023/A:1014229432375. 10.1023/A:1014229432375

[78] Dell’Apa A, Bangley CW, Rulifson RA. Who let the dogfish out? A review of management and socio-economic aspects of spiny dogfish fisheries. Rev Fish Biol Fish. 2015;25:273–95. 10.1007/s11160-014-9379-1. 10.1007/s11160-014-9379-1

[79] Pawson MJ, Ellis J, Dobby H. The evolution and management of spiny dogfish (spurdog) fisheries in the Northeast Atlantic. In: Biology and management of spiny dogfish sharks. Bethesda: American Fisheries Society; 2009. p. 373–90.

[80] Hesthagen T, Wienerroither R, Bjelland O, Byrkjedal I, Fiske P, Lynghammar A, Nedreaas K, Straube N. Fisker: vurdering av pigghå *Squalus acanthias* for Norge. Rødlista for arter 2021. Artsdatabanken; 2021.

[81] Bauer RK. RchivalTag: analyzing and interactive visualization of archival tagging data; 2023

[82] Wickham H, Chang W, Henry L, Pedersen TL, Takahashi K, Wilke C, Woo K, Yutani H, Dunnington D, Posit PBC. ggplot2: create elegant data visualisations using the grammar of graphics; 2023.

[83] Patil I. Visualizations with statistical details: The “ggstatsplot” approach. J Open Source Softw. 2021;6:3167. 10.21105/joss.03167. 10.21105/joss.03167

[84] Patil I, Powell C. ggstatsplot: “ggplot2” based plots with statistical details; 2023

[85] Ripley B, Venables B, Bates DM, ca 1998), KH (partial port, ca 1998), AG, partial port, Firth D. MASS: Support Functions and Datasets for Venables and Ripley’s MASS; 2023.

[86] Powell RA, Mitchell MS. What is a home range? J Mammal. 2012;93:948–58. 10.1644/11-MAMM-S-177.1. 10.1644/11-MAMM-S-177.1

[87] Roesch A, Schmidbauer H. WaveletComp: computational wavelet analysis; 2018.

[88] Cazelles B, Chavez M, Berteaux D, Ménard F, Vik JO, Jenouvrier S, Stenseth NC. Wavelet analysis of ecological time series. Oecologia. 2008;156:287–304. 10.1007/s00442-008-0993-2. 18322705 10.1007/s00442-008-0993-2

[89] Torrence C, Compo GP. A practical guide to wavelet analysis. Bull Am Meteorol Soc. 1998;79:61–78. 10.1175/1520-0477(1998)079%3c0061:APGTWA%3e2.0.CO;2. 10.1175/1520-0477(1998)079<0061:APGTWA>2.0.CO;2

[90] Liu Y. Rectification of the bias in the wavelet power spectrum. J Atmospheric Ocean Technol. 2007;24

[91] Rosch A, Schmidbauer H. WaveletComp 1.1: A guided tour through the R package; 2018.

[92] Kassambara A, Mundt F, factoextra: extract and visualize the results of multivariate data analyses; 2020

[93] Balino BM, Aksnes DL. Winter distribution and migration of the sound scattering layers, zooplankton and micronekton in Masfjorden, western Norway. Mar Ecol Prog Ser. 1993;102:35–50. 10.3354/meps102035

[94] Kaartvedt S. Nocturnal swimming of gammaridean amphipod and cumacean crustacea in Masfjorden, Norway. Sarsia. 1989;74:187–93. 10.1080/00364827.1989.10413427

[95] Prihartato PK, Aksnes DL, Kaartvedt S. Seasonal patterns in the nocturnal distribution and behavior of the mesopelagic fish *Maurolicus muelleri* at high latitudes. Mar Ecol Prog Ser. 2015;521:189–200. 10.3354/meps11139. 10.3354/meps11139

[96] Rasmussen OI, Giske J. Life-history parameters and vertical distribution of *Maurolicus muelleri* in Masfjorden in summer. Mar Biol. 1994;120:649–64. 10.1007/BF00350086. 10.1007/BF00350086

[97] Rokke K. Mesopelagic sound scattering layers and possible explanations for their diel variations in Bjørnafjorden, western Norway (Master Thesis). Bergen: University of Bergen; 2018.

[98] Staby A, Aksnes DL. Follow the light—diurnal and seasonal variations in vertical distribution of the mesopelagic fish *Maurolicus muelleri*. Mar Ecol Prog Ser. 2011;422:265–73. 10.3354/meps08938. 10.3354/meps08938

[99] Andres AM, Slesinger E, Young RE, Saba GK, Saba VS, Phelan BA, Rosendale J, Wieczorek D, White CF, Seibel BA. Thermal sensitivity of metabolic performance in *Squalus acanthias*: efficacy of aerobic scope as a predictor of viable thermal habitat. Mar Ecol Prog Ser. 2024;738:161–85. 10.3354/meps14586

[100] Cushman-Roisin B, Svendsen H. Internal gravity waves in sill fjords: vertical modes, ray theory and comparison with observations. In: Gade HG, Edwards A, Svendsen H, editors. Coastal Oceanography, NATO Conference Series. Boston: Springer; 1983. p. 373–96. 10.1007/978-1-4615-6648-9_21.

[101] Papastamatiou YP, Watanabe YY, Bradley D, Dee LE, Weng K, Lowe CG, Caselle JE. Drivers of daily routines in an ectothermic marine predator: hunt warm, rest warmer? PLoS ONE. 2015;10: e0127807. 10.1371/journal.pone.0127807. 26061229 10.1371/journal.pone.0127807 PMC4489509

[102] Vedor M, Mucientes G, Hernández-Chan S, Rosa R, Humphries N, Sims DW, Queiroz N. Oceanic diel vertical movement patterns of blue sharks vary with water temperature and productivity to change vulnerability to fishing. Front Mar Sci. 2021;8: 688076. 10.3389/fmars.2021.688076. 10.3389/fmars.2021.688076

[103] Vianna GMS, Meekan MG, Meeuwig JJ, Speed CW. Environmental influences on patterns of vertical movement and site fidelity of grey reef sharks (*Carcharhinus amblyrhynchos*) at aggregation sites. PLoS ONE. 2013;8:e60331. 10.1371/journal.pone.0060331. 23593193 10.1371/journal.pone.0060331 PMC3622676

[104] Stehlink LL. Essential fish habitat source document. Spiny dogfish, *Squalus acanthias*, life history and habitat characteristics, NOAA technical memorandum NMFS-NE; 203. Northeast Fisheries Science Center (U.S.); 2007

[105] Nasby-Lucas N, Dewar H, Lam CH, Goldman KJ, Domeier ML. White shark offshore habitat: a behavioral and environmental characterization of the eastern pacific shared offshore foraging area. PLoS ONE. 2009;4:1–14. 10.1371/journal.pone.0008163 PMC2780721 20011032

[106] Pade NG, Queiroz N, Humphries NE, Witt MJ, Jones CS, Noble LR, Sims DW. First results from satellite-linked archival tagging of porbeagle shark, *Lamna nasus*: area fidelity, wider-scale movements and plasticity in diel depth changes. J Exp Mar Biol Ecol. 2009;370:64–74. 10.1016/j.jembe.2008.12.002. 10.1016/j.jembe.2008.12.002

[107] Queiroz N, Humphries NE, Noble LR, Santos AM, Sims DW. Spatial dynamics and expanded vertical niche of blue sharks in oceanographic fronts reveal habitat targets for conservation. PLoS ONE. 2012;7: e32374. 10.1371/journal.pone.0032374. 22393403 10.1371/journal.pone.0032374 PMC3290575

[108] Comfort CM, Weng KC. Vertical habitat and behaviour of the bluntnose sixgill shark in Hawaii. Deep Sea Res Part II Top Stud Oceanogr Biol Deep-Water Chondrichthyans. 2015;115:116–26. 10.1016/j.dsr2.2014.04.005. 10.1016/j.dsr2.2014.04.005

[109] Dewar H, Prince ED, Musyl MK, Brill RW, Sepulveda C, Luo J, Foley D, Orbesen ES, Domeier ML, Nasby-Lucas N, Snodgrass D, Michael Laurs R, Hoolihan JP, Block BA, Mcnaughton LM. Movements and behaviors of swordfish in the Atlantic and Pacific Oceans examined using pop-up satellite archival tags. Fish Oceanogr. 2011;20:219–41. 10.1111/j.1365-2419.2011.00581.x. 10.1111/j.1365-2419.2011.00581.x

[110] Nelson DR, McKibben JN, Strong WR, Lowe CG, Sisneros JA, Schroeder DM, Lavenberg RJ. An acoustic tracking of a megamouth shark, *Megachasma pelagios*: a crepuscular vertical migrator. Environ Biol Fishes. 1997;49:389–99. 10.1023/A:1007369619576

[111] Scheuerell MD, Schindler DE. Diel vertical migration by juvenile sockeye salmon: empirical evidence for the antipredation window. Ecology. 2003;84:1713–20. 10.1890/0012-9658(2003)084[1713:DVMBJS]2.0.CO;2. 10.1890/0012-9658(2003)084[1713:DVMBJS]2.0.CO;2

[112] Cortés E. Standardized diet compositions and trophic levels of sharks. ICES J Mar Sci. 1999;56:707–17. 10.1006/jmsc.1999.0489. 10.1006/jmsc.1999.0489

[113] Martin U, Mallefet J. The diet of deep-water sharks. Deep Sea Res Part Oceanogr Res Pap. 2023;192:103898. 10.1016/j.dsr.2022.103898. 10.1016/j.dsr.2022.103898

[114] Giske J, Salvanes AGV, Wakili SM, Aadnesen A. Vertical distribution and trophic interactions of zooplankton and fish in Masfjorden, Norway. Sarsia. 1990;75:65–81. 10.1080/00364827.1990.10413442

[115] Christiansen S, Klevjer TA, Røstad A, Aksnes DL, Kaartvedt S. Flexible behaviour in a mesopelagic fish (*Maurolicus muelleri* ). ICES J Mar Sci. 2021;78:1623–35. 10.1093/icesjms/fsab075. 10.1093/icesjms/fsab075

[116] Christiansen S, Titelman J, Kaartvedt S. Nighttime swimming behavior of a mesopelagic fish. Front Mar Sci. 2019. 10.3389/fmars.2019.00787. 10.3389/fmars.2019.00787

[117] Onsrud MSR, Kaartvedt S, Røstad A, Klevjer TA. Vertical distribution and feeding patterns in fish foraging on the krill *Meganyctiphanes norvegica*. ICES J Mar Sci. 2004;61:1278–90. 10.1016/j.icesjms.2004.09.005. 10.1016/j.icesjms.2004.09.005

[118] Solberg I, Røstad A, Kaartvedt S. Ecology of overwintering sprat (*Sprattus sprattus*). Prog Oceanogr. 2015;138:116–35. 10.1016/j.pocean.2015.08.003. 10.1016/j.pocean.2015.08.003

[119] Stiti DJS. Spatiotemporal variation in the density distribution of sprat (*Sprattus sprattus*) in Hardangerfjorden and Sognefjorden (Master Thesis). Bergen: University of Bergen; 2022.

[120] Ellis JR, Pawson MG, Shackley SE. The comparative feeding ecology of six species of shark and four species of ray (Elasmobranchii) in the North-East Atlantic. J Mar Biol Assoc U K. 1996;76:89–106. 10.1017/S0025315400029039. 10.1017/S0025315400029039

[121] Sulikowski JA, Prohaska BK, Carlson AE, Cicia AM, Brown CT, Morgan AC. Observations of neonate spiny dogfish, *Squalus acanthias*, in Southern New England: a first account of a potential pupping ground in the Northwestern Atlantic. Fish Res. 2013;137:59–62. 10.1016/j.fishres.2012.08.018. 10.1016/j.fishres.2012.08.018

[122] Åslein EH. Killer whale (Orcinus orca) predation on harbour porpoise (*Phocoena phocoena*) in Hardangerfjord, Western Norway (Master Thesis). Oslo: University of Oslo; 2023.

[123] Watanabe YY, Ito M, Takahashi A. Testing optimal foraging theory in a penguin–krill system. Proc R Soc B Biol Sci. 2014;281:20132376. 10.1098/rspb.2013.2376. 10.1098/rspb.2013.2376 PMC3924065 24478293

[124] Wallman HL, Bennett WA. Effects of parturition and feeding on thermal preference of Atlantic stingray, *Dasyatis sabina* (Lesueur). Environ Biol Fishes. 2006;75:259–67. 10.1007/s10641-006-0025-1

[125] Tokunaga S, Watanabe YY, Kawano M, Kawabata Y. Factors affecting gestation periods in elasmobranch fishes. Biol Open. 2022;11:bio059270. 10.1242/bio.059270. 35686686 10.1242/bio.059270 PMC9194679

[126] Sims DW. Differences in habitat selection and reproductive strategies of male and female sharks. In: Ruckstuhl K, Neuhaus P, editors. Sexual Segregation in Vertebrates. Cambridge: Cambridge University Press; 2006. p. 127–47. 10.1017/CBO9780511525629.009.

[127] Wearmouth VJ, Sims DW. Chapter 2 sexual segregation in marine fish, reptiles, birds and mammals: behaviour patterns, mechanisms and conservation implications. In: Advances in marine biology. Academic Press, p. 107–170; 2008. 10.1016/S0065-2881(08)00002-3 10.1016/S0065-2881(08)00002-3 18929064

[128] Hight BV, Lowe CG. Elevated body temperatures of adult female leopard sharks, *Triakis semifasciata*, while aggregating in shallow nearshore embayments: evidence for behavioral thermoregulation? J Exp Mar Biol Ecol. 2007;352:114–28. 10.1016/j.jembe.2007.07.021. 10.1016/j.jembe.2007.07.021

[129] Jirik KE, Lowe CG. An elasmobranch maternity ward: female round stingrays *Urobatis halleri* use warm, restored estuarine habitat during gestation. J Fish Biol. 2012;80:1227–45. 10.1111/j.1095-8649.2011.03208.x. 22497381 10.1111/j.1095-8649.2011.03208.x

[130] Hisaw FL, Albert A. Observations on the reproduction of the spiny dogfish, *Squalus acanthias*. Biol Bull. 1947;92:187–99. 10.2307/1538305. 20249523 10.2307/1538305

[131] Fiskeridirektoratet. Saksdokumenter til reguleringsmøtet (No. SAK 5/2018); 2018.

[132] Gaitán-Espitia JD, Gómez D, Hobday AJ, Daley R, Lamilla J, Cárdenas L. Spatial overlap of shark nursery areas and the salmon farming industry influences the trophic ecology of *Squalus acanthias* on the southern coast of Chile. Ecol Evol. 2017;7:3773–83. 10.1002/ece3.2957. 28616174 10.1002/ece3.2957 PMC5468132

[133] Norges Fiskarlag. Sak-32-2019-2019/00257-6; 2019.

[134] Hatlebrekke HH, Vølstad, Kolding J. The coastal reference fleet 2007–2019 (No. 2021-52). Bergen: Institute of Marine Research; 2021.

[135] Lind S. Shedding light on the extent and patterns of spurdog (*Squalus acanthias*) bycatch in the Norwegian coastal fisheries (Master thesis); 2023.

[136] Fraser HM, Greenstreet SPR, Piet GJ. Taking account of catchability in groundfish survey trawls: implications for estimating demersal fish biomass. ICES J Mar Sci. 2007;64:1800–19. 10.1093/icesjms/fsm145. 10.1093/icesjms/fsm145

[137] Junge C, Ferter K, Klöcker CA, Bjelland O, Albretsen J, Økland F, Jac R, Andrade H, Albert OT. in review. Tag attachment innovation reveals year-round coastal association of female spurdog (*Squalus acanthias* Linnaeus, 1758) in Norwegian waters. 10.1111/jfb.16000 PMC12120337 39721573

