# Supplementary material for: Seasonal habitat use and diel vertical migration in female spurdog in Nordic waters
Source: Mov Ecol. 2024 Sep 6;12:62. doi: 10.1186/s40462-024-00498-2 (PMC11380420; doi:10.1186/s40462-024-00498-2)
Supplement: Supplementary file 1 — Additional file 1. [file 40462_2024_498_MOESM1_ESM.pdf]

Supporting information for

Seasonal habitat use and diel vertical migration in female spurdog in Nordic waters

C. Antonia Klöcker, Ole Thomas Albert, Keno Ferter, Otte Bjelland, Robert J. Lennox, Jon Albretsen, Lotte Pohl, Lotte Svengård Dahlmo, Nuno Queiroz, Claudia Junge

DOI: 10.1186/s40462-024-00498-2

Corresponding author: Claudia Junge
E-mail: claudia.junge@hi.no

This file includes:

- Supplementary Details (S1-3)
- Supplementary Table (S1)
- Supplementary Figures (S1-S19)
- Supplementary References

Detail S1: Light level metric

Material

Light level is a measure specific to the Wildlife Computers product. It is a relative measurement of light in the blue band (415- 460nm), with 25-225 presenting end points on a logarithmic scale. While it does not permit a direct conversion to light intensity, light levels of 150, 110, and 70 correlate to 10-5, 10-7, and 10-9 Wcm-2 respectively. Note, however, that light level values are not directly comparable between individual tags.

Detail S2: Periodicity in vertical movement

Methods & Results - Sensitivity test for harmonics in continuous wavelet analysis

To test the effect of a deviation from a sine-signal in the depth time series (e.g. due to rapid descents and ascents or different light-induced dive regimes) on the power distribution of the continuous wavelet analysis, a wavelet analysis was performed on a simulated strict diel vertical pattern for 180 days switching from 80 to 200 m depth in a 12:12, 6:18 and 3:21 h rhythm. The 12:12h pattern represents conditions during equinox while 3:21 corresponds to a light regime during solstice in the study region.

Results visualised as scalograms in Figure S19 revealed that powers other than the 1-day period can be significant even if only a 1-day pattern is simulated. This was particularly the case for patterns that deviated most from a sine curve, such as 18:6 and 21:3h patterns. In case of the 6:18h pattern, the first harmonic at 12h was significant across the time series. In case of the 3:21h pattern this was the case for the 12h and higher periods. The 12:12h pattern was not associated with significant periods at 12h, and only occasionally showed significant 6h periods.

Discussion - Semi-diurnal pattern

A recent study on starry smooth-hound sharks (*Mustelus asterias*) in the North Sea found semi-diurnal periods to be associated with tidally linked resting behaviour at the bottom (Pohl, 2023). Based on tank experiments suggesting buccal ventilation capabilities in spurdog (Kelly et al., 2020; Skubel et al., 2020), this could be a plausible for spurdog as well. However, for the data presented herein, this semi-diurnal pattern seems unlikely to be indicative of resting behaviour as sharks used variable depths and showed elevated number of fast starts during significant diel patterns. While the maximal tidal range with 1.0-1.5 m (https://sehavniva.no) is rather small in the study region, the northward propagation of the tidal wave along the coast induces internal waves upon entering the fjords, either at the pycnocline between the surface and intermediate layer (~5-10m depth) or the sill level, which in case of the Hardangerfjord is at around 150m depth (Cushman-Roisin and Svendsen, 1983). Fluctuations in the density field associated with internal waves result in a significant vertical displacement of particles such as prey items with a phase corresponding to the astronomical tidal forcing where the semi-diurnal lunar constituent (M2) with a period of 12.42h is the most prominent. Thus, tidal processes are likely affecting the depth use of the sharks as reflected in the semi-diurnal cycles detected in the wavelet analysis. While the co-occurrence of the semi-diurnal with the diurnal cycles might be linked to sharks selecting sill depth during DVM behaviour, the absence of semi-diurnal cycles when sharks spend substantial time above or around the pycnocline in summer, however, leaves some open questions. Rather than a tidal signal, semi-diurnal cycles might be harmonics resulting from non-strictly sinusoidal movement behaviour. This is the case for example when

sharks undertake strict DVM with U-shaped dives involving extended periods at a constant depth during day and night with rapid ascents and descents during dusk. Deviations from a sine curve increase during solstice (i.e. long or short photoperiods) and can produce artificial but significant semi-diurnal periods or higher harmonics (see Methods & Results in Detail S2).

Detail S3: Other possible drivers of vertical movement behaviour

Discussion - Hypoxia, offspring predation risk and social avoidance

In addition to temperature, the vertical habitat use and behaviour of sharks has been shown to be affected and limited by low oxygen levels (Nasby-Lucas et al., 2009; Abascal et al., 2011; Sims, 2019; Vedor et al., 2021; Santos et al., 2021). With shallow sills limiting the influx of dense oxygen-rich waters into many of the fjords in the study region (outer sill of Bergen fjord system at 130m, sills to individual fjords often shallower), oxygen levels in the fjord basins are rather low (~2-4 ml/l) (Søvik et al., 2023). The development, persistence, and vertical extent of hypoxic conditions is highly variable between and within fjords, dictated by length, width, depth, orientation as well as the sill depth of the fjord. However, in line with multi-decadal warming, the frequency of such high-density intrusions, commonly occurring in late spring to early summer, has decreased over the past 30 years, leading to decreased fjord basin ventilation (Aksnes et al., 2019; Darelius, 2020; Pitcher et al., 2021). In lab experiments with spurdog, hypoxic conditions have been shown to limit maximum performance (Andres, 2022) and affect physiological processes (Swenson et al., 2005; Zimmer and Wood, 2014), however, implications for habitat selection remain unknown. If oxygen conditions in the deeper water are limiting, which might be amplified due to increasing oxygen demands of developing embryos, sharks might avoid deeper habitats as observed in summer and autumn periods in tagged individuals.

Indication for a deep-water renewal in many fjords on the Norwegian west coast in spring 2021 (Rosland, 2022; Søvik et al., 2023), suggests possible effects to be limited for the subsequent tracking period. Nevertheless, differences in renewal events between years or fjords might contribute to differences between tagging cohorts e.g. 2021 and 2022 or contribute to inter-individual variation due to use of different fjord basins. A recent thesis on the vertical distribution on the velvet belly lanternshark (*Etmopterus spinax*), also displaying DVM behaviour, found no effect on depth use before and after the renewal event in Masfjorden 2021 (Rosland, 2022). Further, the vertical distribution of the common prey species sprat (*Sprattus sprattus*) was shown to be unaffected by deoxygenation in this system (Kaartvedt et al., 2009). Lab-based choice experiments together with tag-based oxygen measurements and mapping of fine scale horizontal habitat use are needed to assess if the depth use of spurdogs in this system is oxygen-driven or limited.

While we assume the effect of predators in the system to be negligible for mature females given the few limited presence of potential predators in the system for this comparatively large fish (see main paper), predation evasion might play an elevated role during time of parturition (around Nov-Jan) as offspring are more vulnerable and predator naïve. According to the predation-risk hypothesis, female habitat selection might favour offspring security over optimal resource availability (Wearmouth and Sims, 2008). This might explain the lower fast starts at shallower depth in summer and autumn. The lack of higher horizontal resolution, however, does not permit inferences on whether females selected shallow and likely predator poor habitats at the flanks of the fjords or bays or whether they chose surface layers with deeper bathymetry, which would present a rather predation-prone environment. While sporadic dives to mesopelagic depths during summer and autumn might indicate the latter, the steep bathymetry in many

of the fjords sets shallow and deep habitats in proximity. Further, locations and depths at which sharks were tagged and recaptured in autumn suggest sharks to have resided in shallower waters along steep drops in bathymetry close to shore at the time point of capture, indicating that sharks may have resided in shallower, more protected habitats during this season.

As habitat use in spurdog is often sexually segregated (Ford, 1921; Shepherd et al., 2002; Stehlink, 2007; Dell'Apa et al., 2014), the prevalence of certain modes of depth use such as DVM or the occupancy of shallow, warm waters might be a result of social avoidance behaviour. In the small-spotted catshark, for example, females hide in shallow waters to avoid males that dominate in deeper waters (Sims et al., 2001; Sims, 2006). As this study focussed on mature, pregnant females, inferences about the contribution of avoidance behaviour remain beyond the scope of this paper.

Supplementary Table

Table S1: Tagging information for all PSAT tagged female spurdogs. This includes tag ID, dates of tagging and surfacing due to programmed pop-off, early tag detachment or shark recapture (DATEon, DATEoff), total length (TL), coordinates of tagging and surfacing locations (LOCon, LOCoff), the reason for track termination and the number of days at liberty (DAYS) and those days used for analysis (DAYS used). PD denotes premature detachment.

Shark ID	Tag ID	TL [cm]	DATEon	LOCon	DATEoff	LOCoff	REASON	DAYS	DAYS used
**1**	17P0023	95	27/11/2019	60.029, 5.338	25/05/2020	60.185, 5.333	interval	180	179
**2**	17P0749	95	27/11/2019	60.029, 5.338	25/05/2020	59.791, 5.623	interval	180	179
**3**	17P0796	87	26/11/2019	60.026, 5.336	24/05/2020	58.215, 5.959	interval	180	180
**4**	17P0864	93	26/11/2019	60.028, 5.338	24/05/2020	59.866, 5.690	interval	180	180
**5**	17P0866	97	26/11/2019	60.028, 5.338	24/05/2020	59.395, 5.196	interval	180	116
**6**	20P2042	96	11/12/2020	60.022, 5.337	22/07/2021	59.670, 5.446	PD	223	222
**7**	20P2044	87	08/12/2020	60.022, 5.337	26/06/2021	59.785, 5.602	PD	200	199
**8**	20P2045	96	11/12/2020	60.022, 5.337	30/05/2021	60.370, 5.648	PD	170	169
**9**	20P2046	92	12/12/2020	60.022, 5.337	20/04/2021	59.261, 6.152	recapture	129	128
**10**	20P2047	91	12/12/2020	60.022, 5.337	15/07/2021	59.810, 5.541	PD	215	214
**11**	20P2660	112	04/11/2021	60.518, 5.164	20/09/2022	60.554, 5.018	recapture*	320	319
**12**	21P0036	106	04/11/2021	60.518, 5.164	31/10/2022	60.608, 5.467	interval	361	360
**13**	21P0037	117	04/11/2021	60.518, 5.164	29/01/2022	60.555, 5.392	sensor error	86	82
**14**	21P0038	112	04/11/2021	60.518, 5.164	31/10/2022	60.757, 5.716	interval	361	360
**15**	21P0039	109	04/11/2021	60.518, 5.164	31/10/2022	60.518, 5.164	interval	361	360
**16**	21P2088	106	25/10/2022	60.518, 5.164	28/07/2023	60.37, 5.063	const. pressure**	274	273
**17**	21P2202	107	25/10/2022	60.518, 5.164	25/10/2023	60.568, 4.967	recapture*	365	364
**18**	21P2203	115	25/10/2022	60.518, 5.164	26/10/2023	60.405, 5.244	interval	366	364
**19**	21P2209	112	25/10/2022	60.518, 5.164	26/10/2023	60.339, 5.172	interval	366	364
**20**	21P2204	116	25/10/2022	60.518, 5.164	NA	NA	non report	NA	NA
**21**	21P2041	101	25/10/2022	60.518, 5.164	NA	NA	non report	NA	NA

**dissected post-mortem **from 26.07.2023 where constant pressure triggered release and popoff two days late*

Supplementary Figures

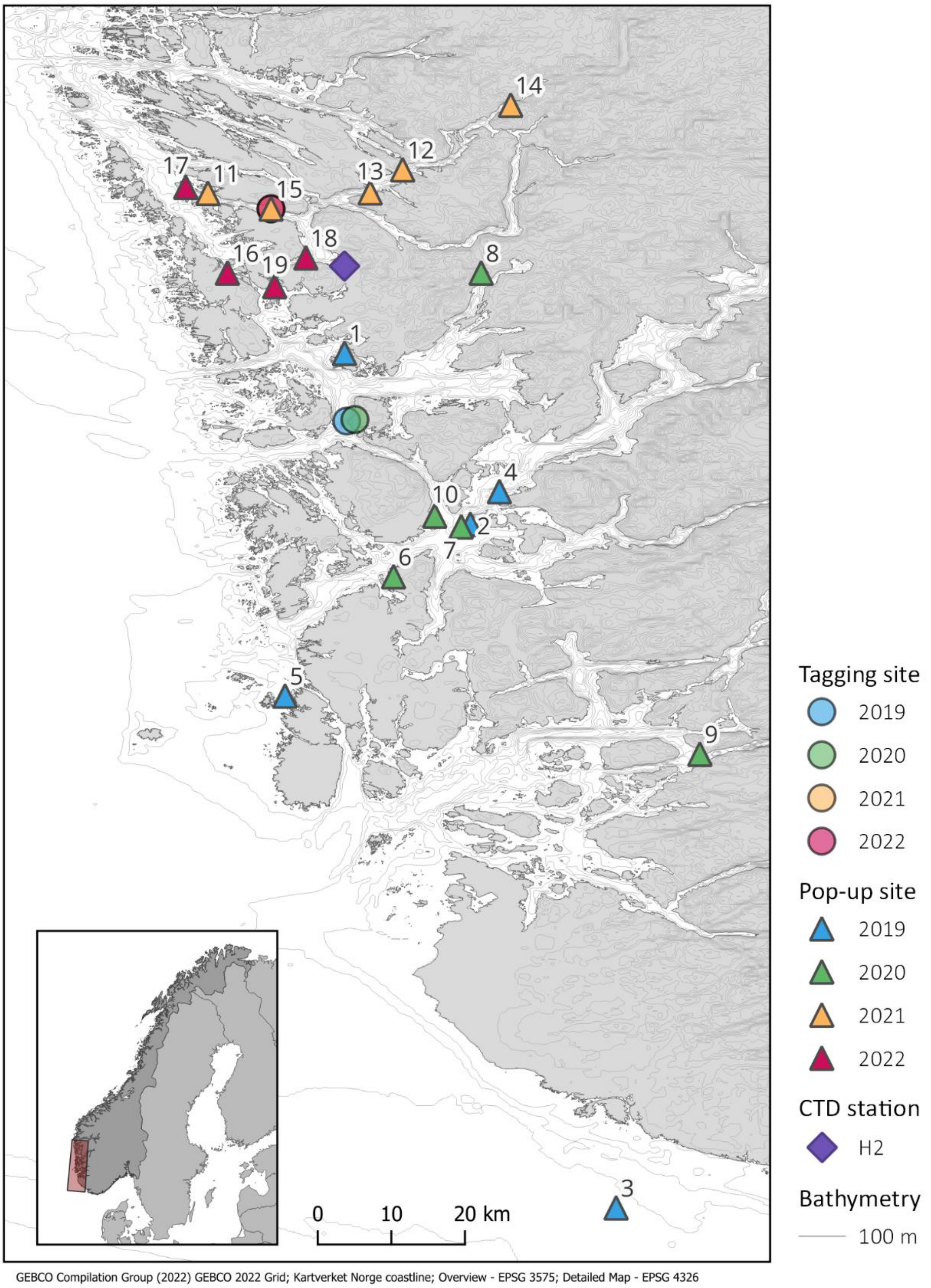

Figure S1: Tagging (circles) and pop-up sites (triangles) of PSATs from 19 female spurdogs deployed in autumn 2019 to 2022 along the southwestern Norwegian coast. Note that tagging locations in 2021 and 2022 were identical. Numbers associated with pop-up sites (triangles) indicate respective shark IDs. Note that colours represent tagging year, respective pop-ups occurred in the consecutive year. Bathymetry and elevation contours are shown every 100m.

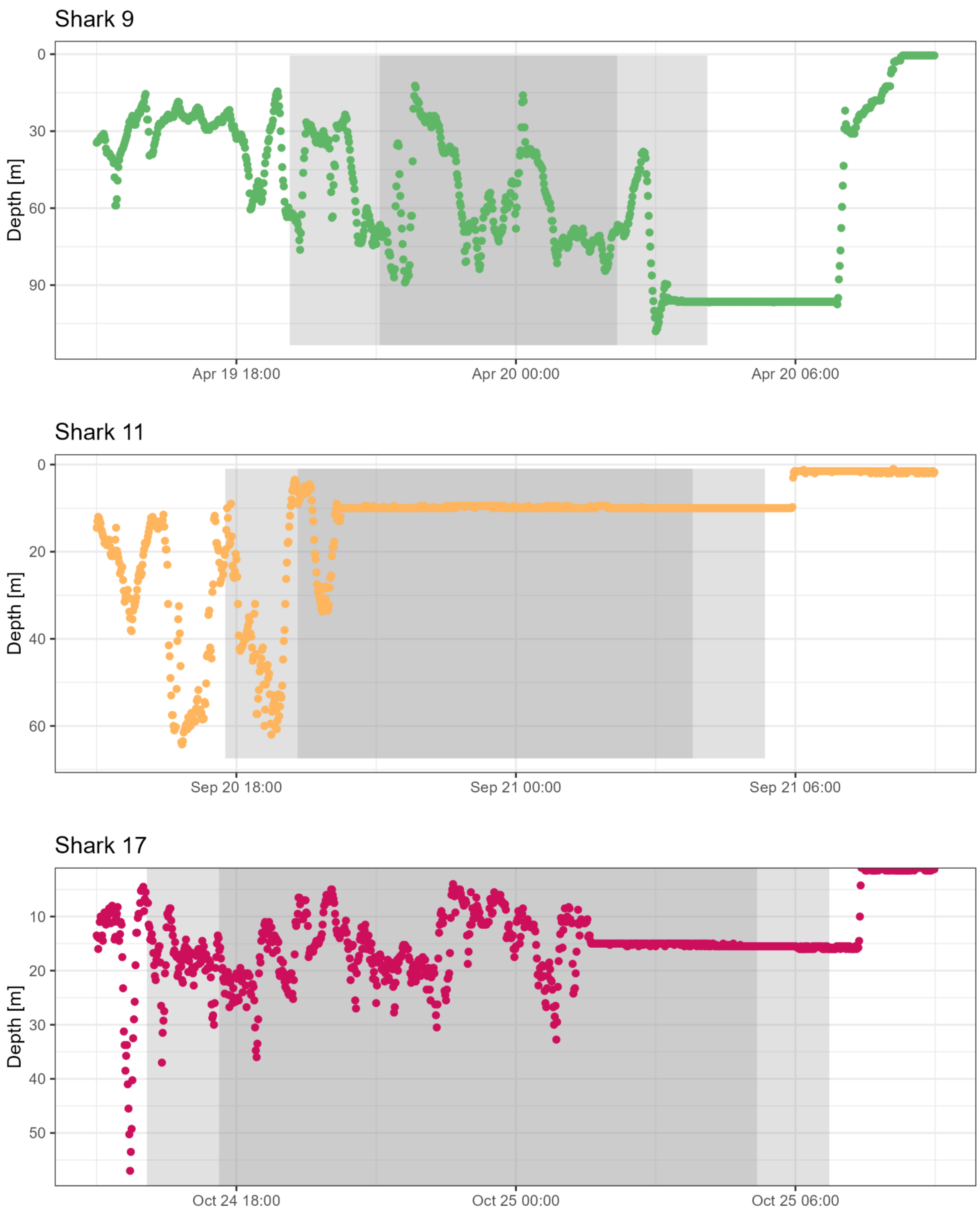

Figure S2: Depth-timeseries for recapture events for sharks 9, 11, and 17. Data are based on 1-minute interval median depths, colours indicate tagging cohort association. Grey polygons mark night defined by sunset and sunrise and nautical dusk and dawn (sun 12° below horizon, winter only) around the tagging location.

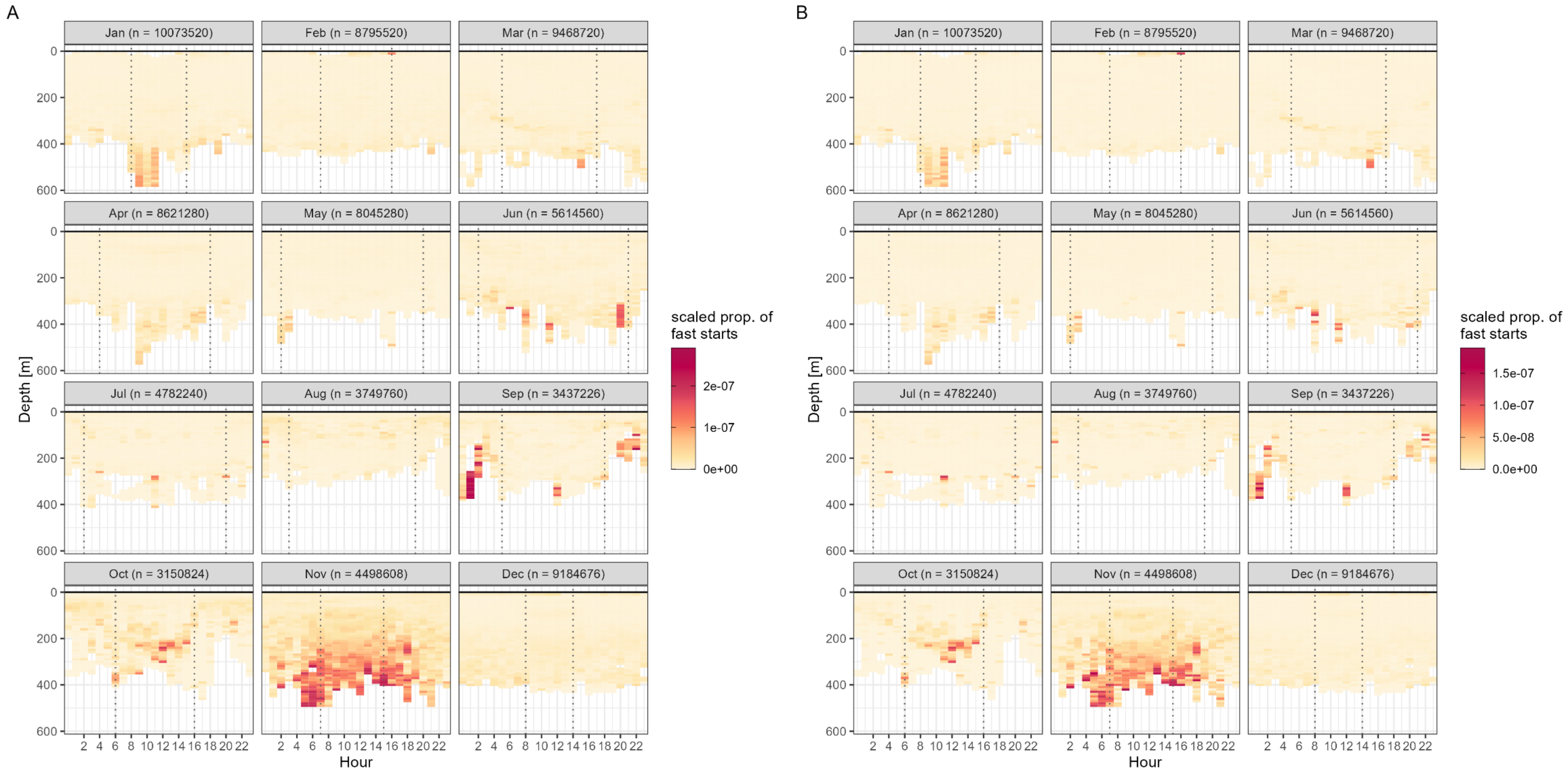

Figure S2: Activity seascapes for each month based on 5-sec resolution PSAT data with fast starts calculated using a (A) 97% and (B) 99% MA threshold. Data are aggregated by month in 10m depth bins and hour of day. Dotted vertical lines mark the median hour of sunrise and sunset for a given month.

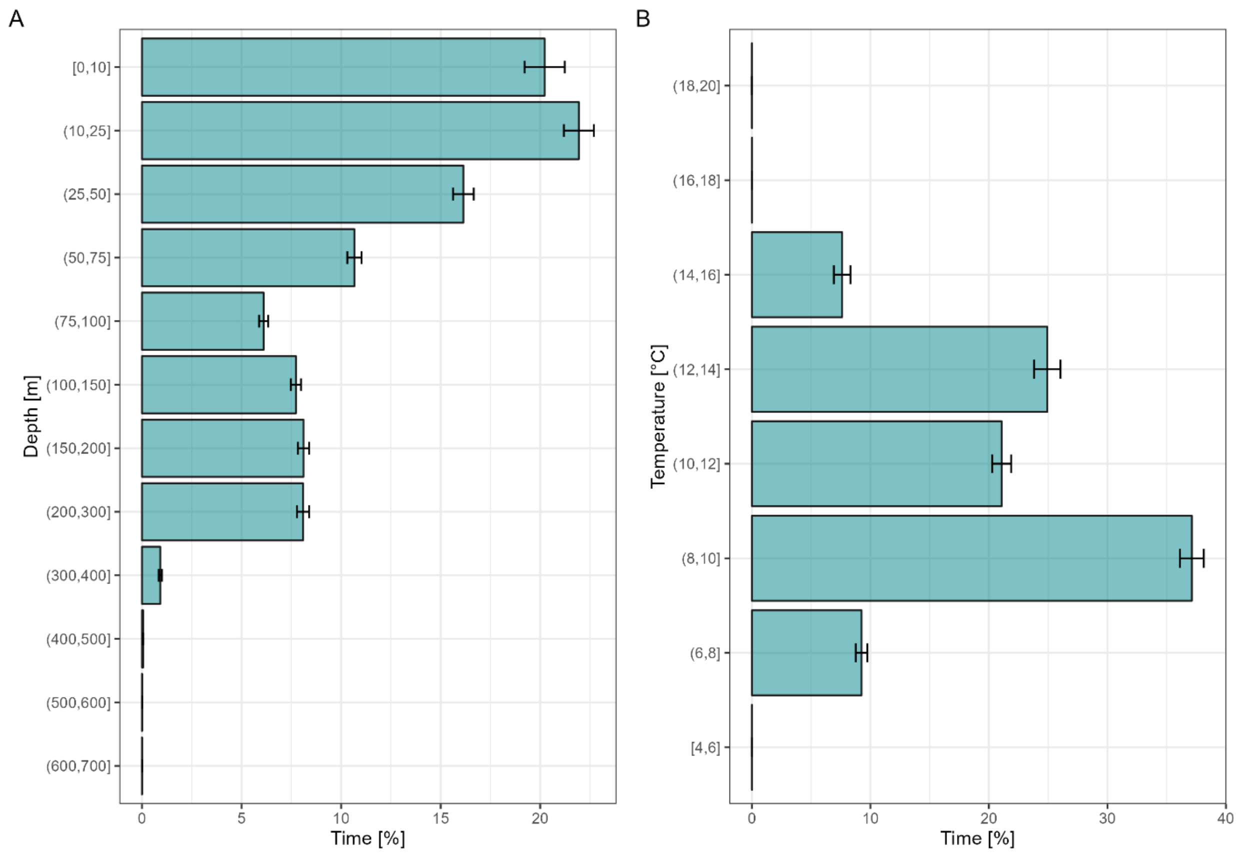

Figure S4: Time-at-depth (A) and time-at-temperature (B) histograms for the entire deployment. Bars represent mean percentage of time (summarised by date) spent across individuals in each of the twelve depth and eight temperature bins. The means were weighted by the reciprocal of data points per Julian day to account for the heterogenic temporal coverage due to different deployment lengths. Error bars indicate standard errors.

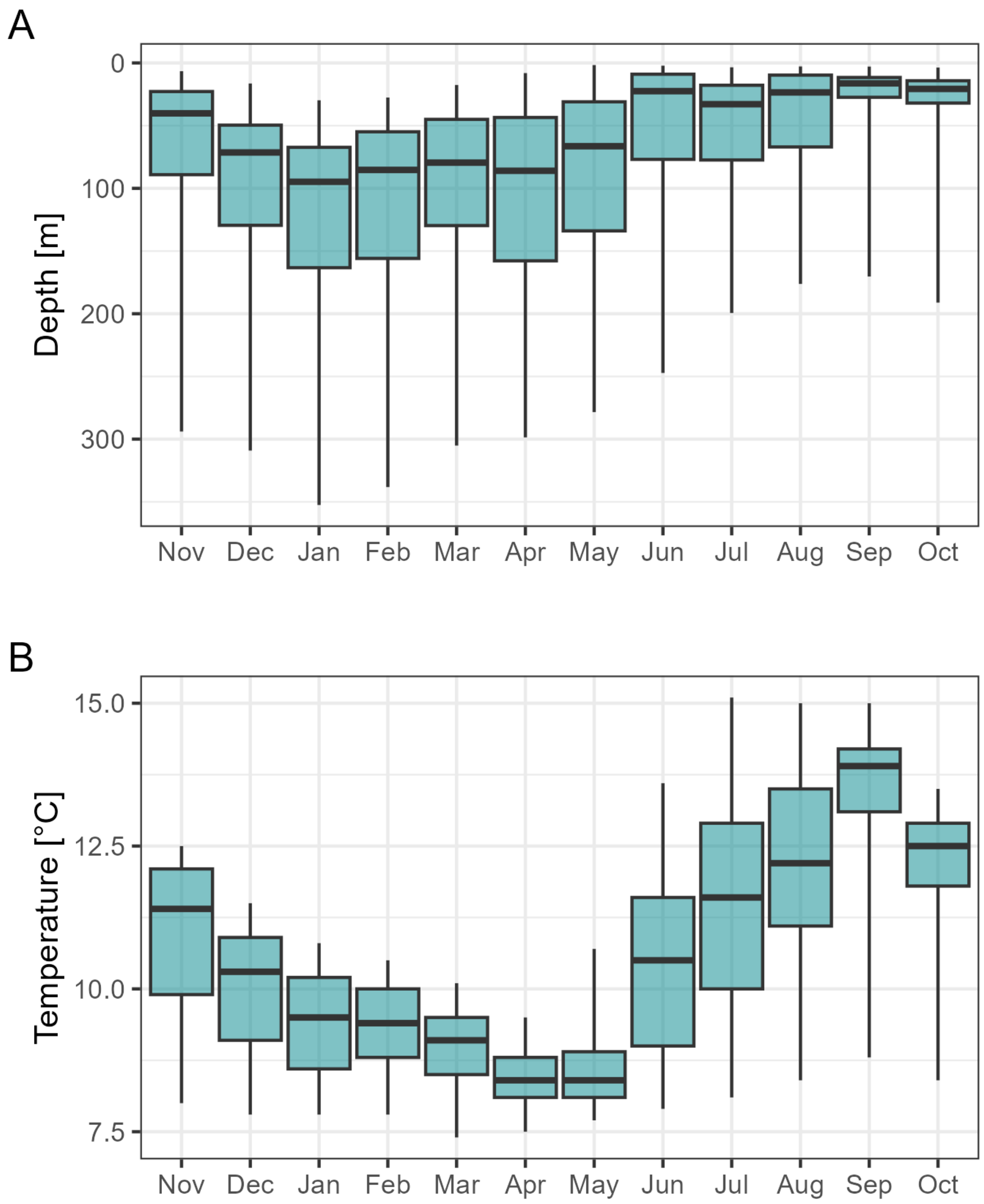

Figure S5: Average daily depth (A) and temperature (B) use across the different months of deployment based on 1-minute interval median depth and temperature values. Boxplots show the average median, boxes indicate the interquartile range (IQR – 25 and 75% quartiles) and whiskers extent to the average daily minimum and maximum depth and temperature per month.

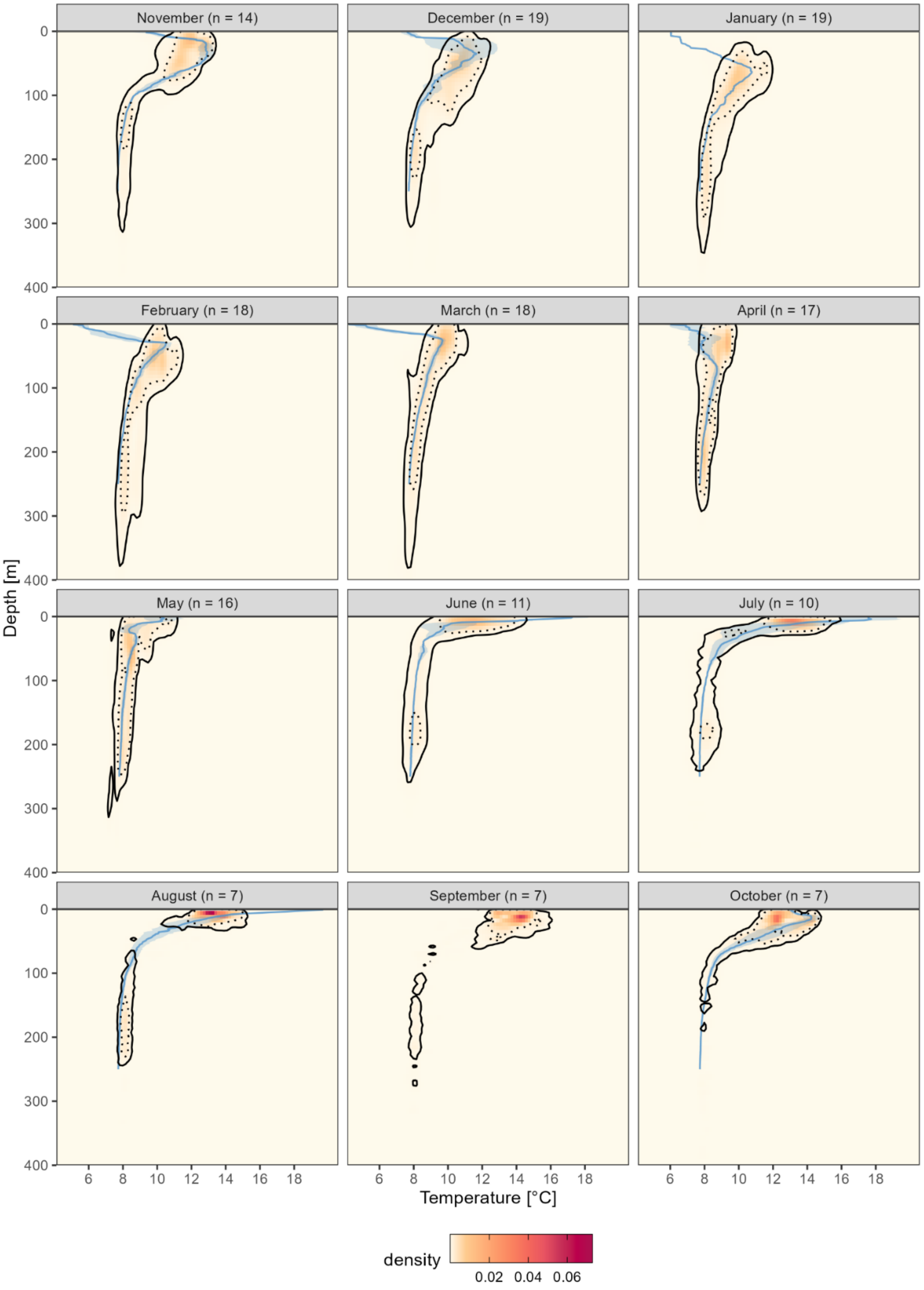

Figure S3: Realised depth-temperature niche by female spurdogs in the context of available habitat for each month. Number of sharks contributing to each monthly plot are indicated. Red colours denote the density of hourly datapoints within a given grid cell. Black dotted and solid lines indicate the niche space that encompasses 50% and 95% of these data, respectively. The profiles in blue are CTD profiles from the hydrographic station H2 in Hardangerfjord with the mean and corresponding standard errors shown for every meter down to 250m in the given month. For visualisation purposes, the y-axis was limited to 400m depth.

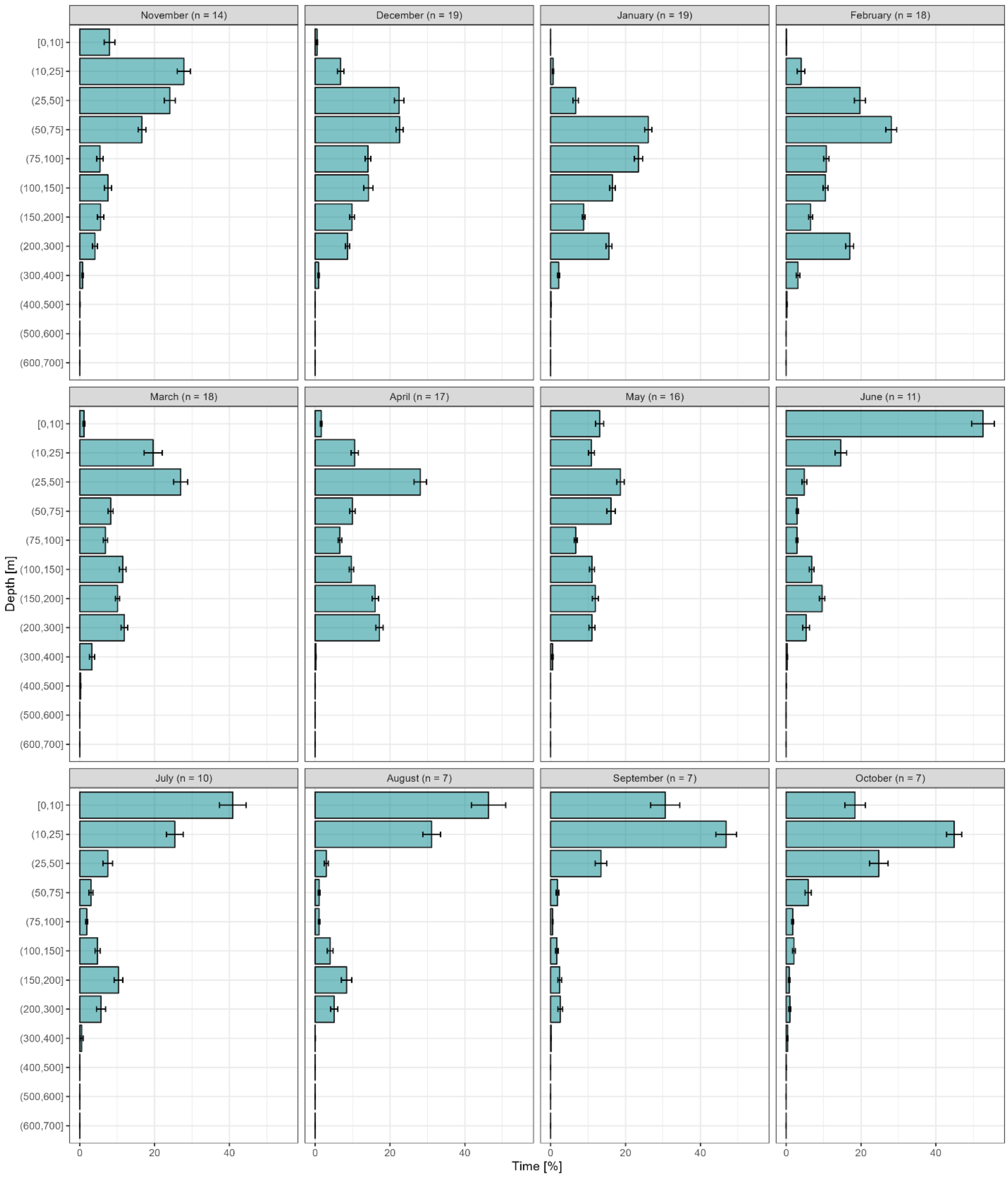

Figure S7: Time-at-depth histograms for each month of tag attachment. Bars represent mean percentage of time (summarised by date) spent across individuals in each of the twelve defined depth ranges, with error bars indicating standard errors. Number of sharks contributing to each monthly plot are indicated.

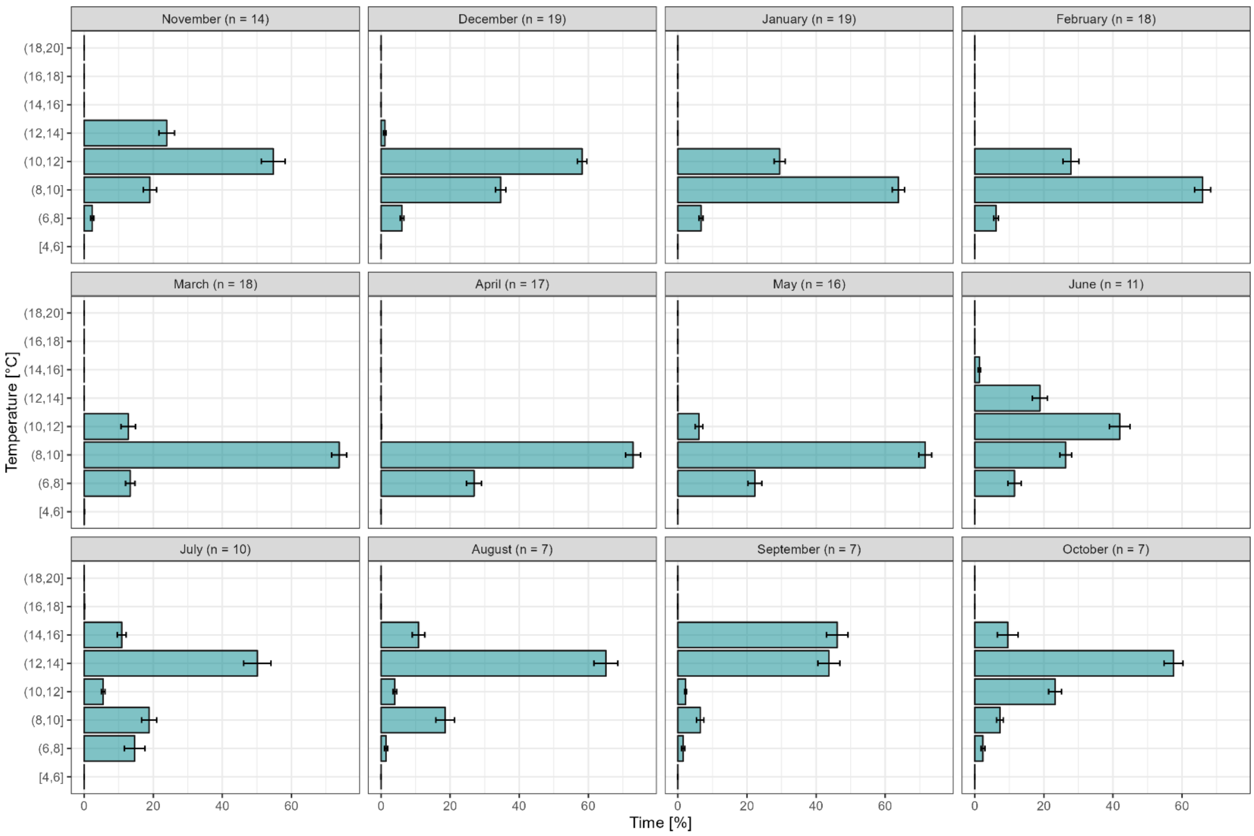

Figure S8: Time-at-temperature histograms for each month of tag attachment. Bars represent mean percentage of time (summarised by date) spent across individuals in each of the eight defined temperature ranges, with error bars indicating standard errors. Number of sharks contributing to each monthly plot are indicated.

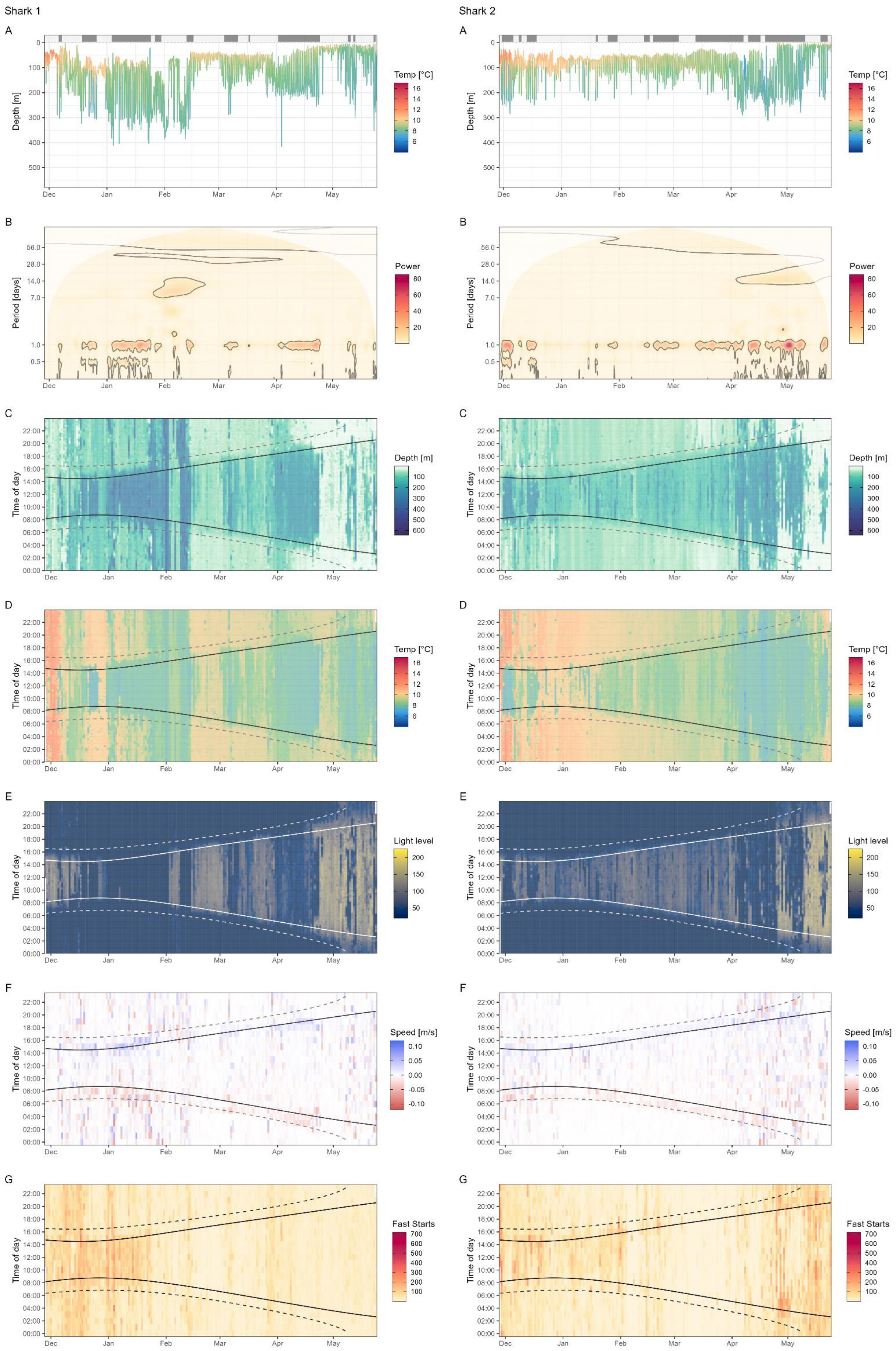
Shark 1
Shark 2
A
Depth [m]
Temp [°C]
16
14
12
10
8
6
Dec
Jan
Feb
Mar
Apr
May
B
Period [days]
56.0
28.0
14.0
7.0
1.0
0.5
Power
80
60
40
20
C
Time of day
Depth [m]
100
200
300
400
500
600
D
Temp [°C]
E
Light level
200
150
100
50
F
Speed [m/s]
0.10
0.05
0.00
-0.05
-0.10
G
Fast Starts
700
600
500
400
300
200
100

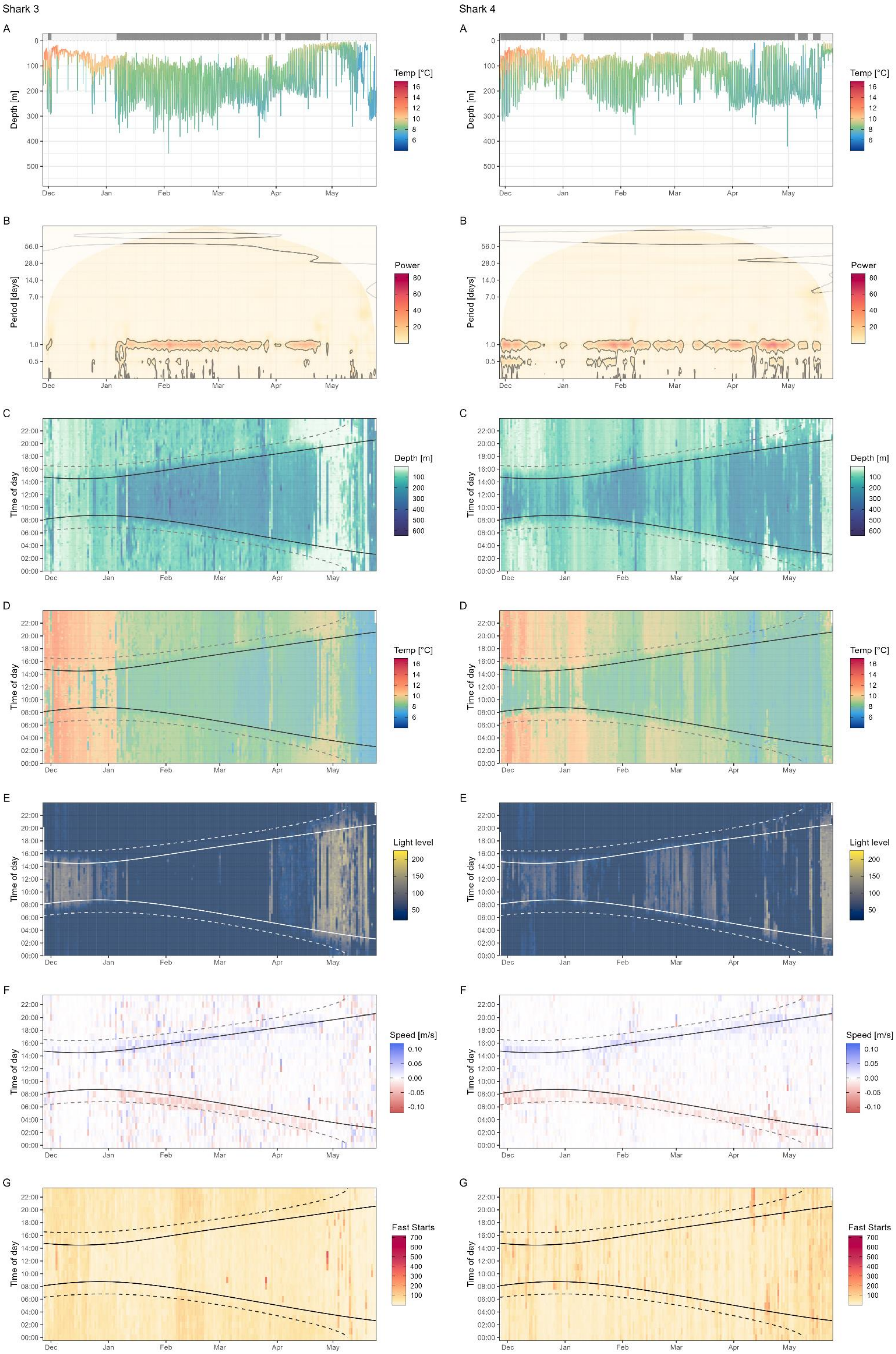
Shark 3
Shark 4
A
B
C
D
E
F
G
Depth [m]
Temp [°C]
Period [days]
Power
Time of day
Depth [m]
Temp [°C]
Light level
Speed [m/s]
Fast Starts
Dec
Jan
Feb
Mar
Apr
May

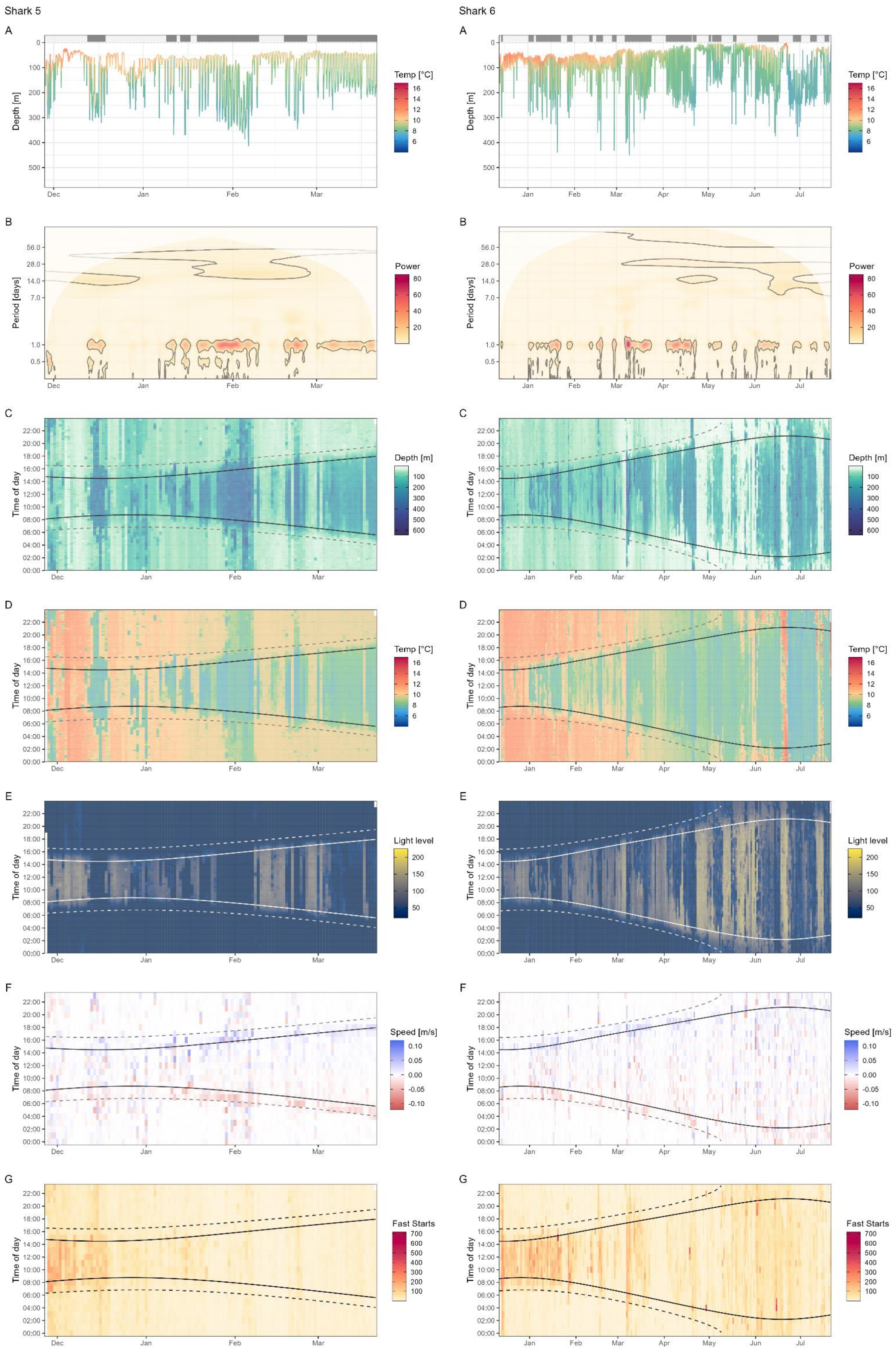
Shark 5
Shark 6
A
Depth [m]
Temp [°C]
16
14
12
10
8
6
0
100
200
300
400
500
Dec
Jan
Feb
Mar
Apr
May
Jun
Jul
B
Period [days]
56.0
28.0
14.0
7.0
1.0
0.5
Power
80
60
40
20
C
Time of day
Depth [m]
100
200
300
400
500
600
22:00
20:00
18:00
16:00
14:00
12:00
10:00
08:00
06:00
04:00
02:00
00:00
D
Temp [°C]
E
Light level
200
150
100
50
F
Speed [m/s]
0.10
0.05
0.00
-0.05
-0.10
G
Fast Starts
700
600
500
400
300
200
100

Shark 7

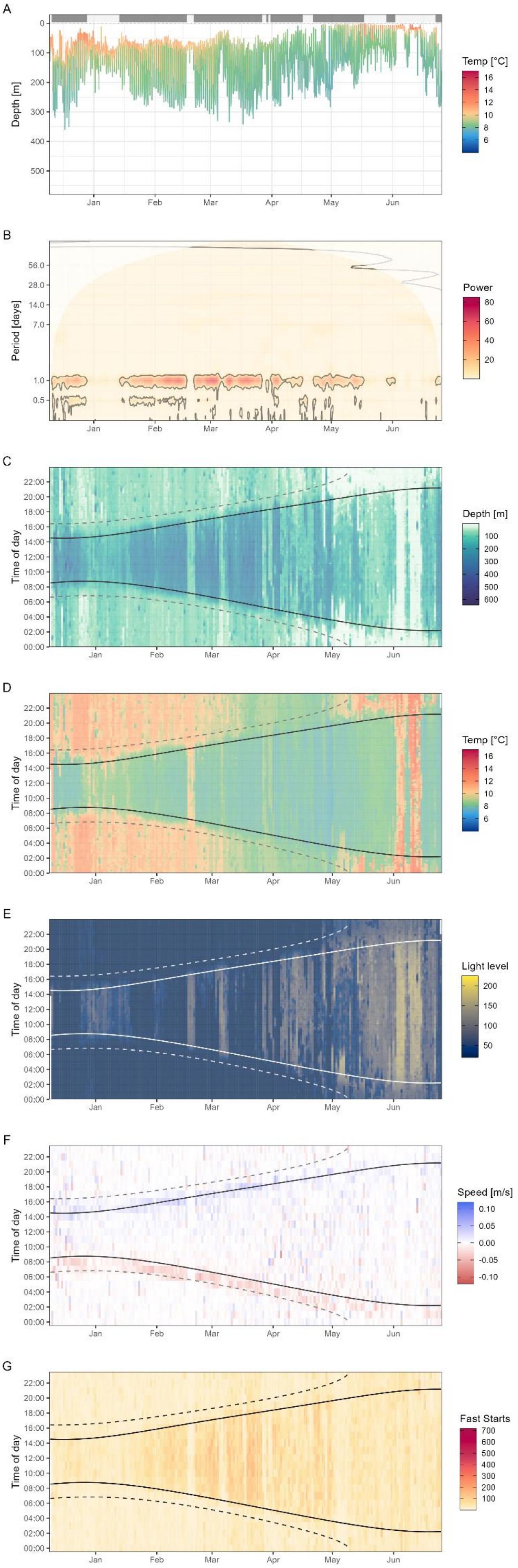

Shark 8

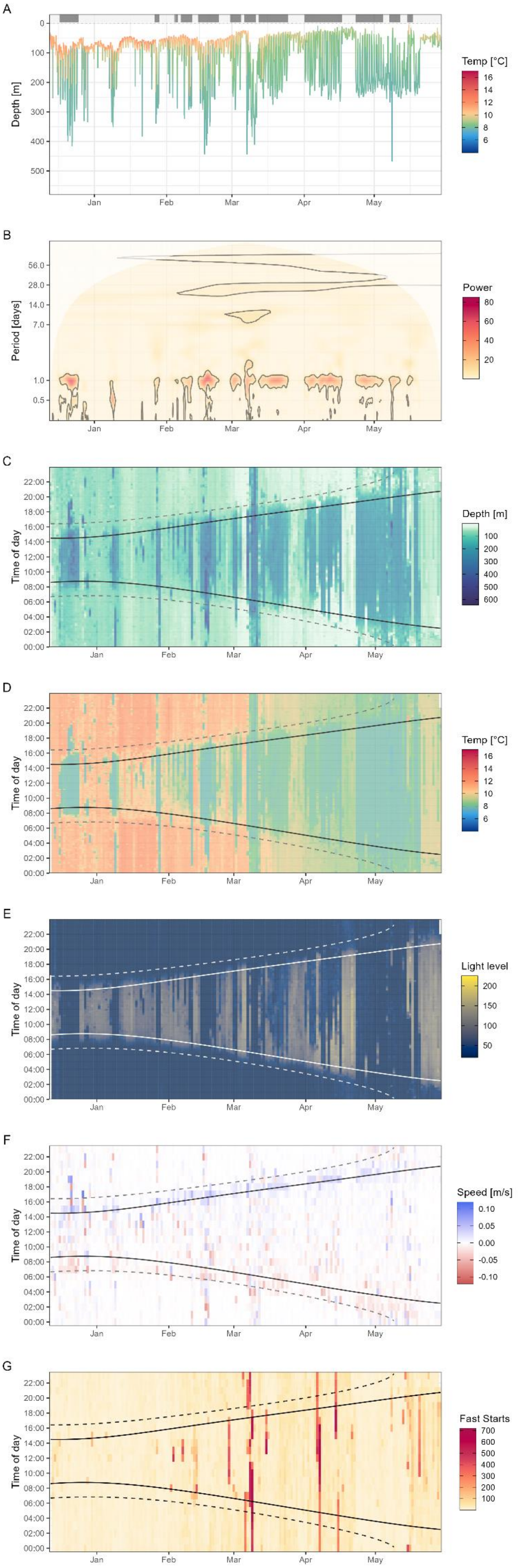

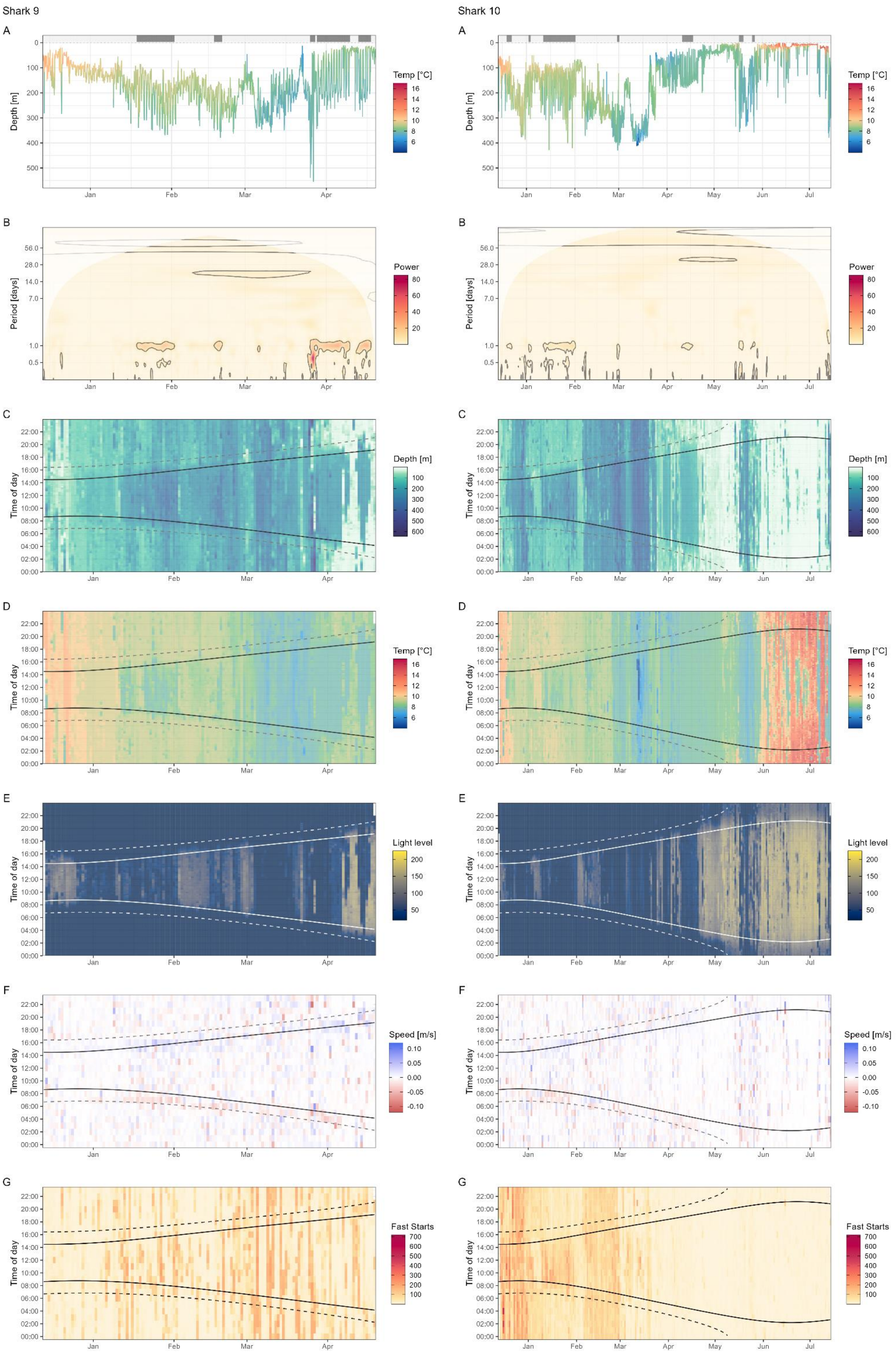

Shark 9
Shark 10
A
Depth [m]
Temp [°C]
B
Period [days]
Power
C
Time of day
Depth [m]
D
Temp [°C]
E
Light level
F
Speed [m/s]
G
Fast Starts
Jan
Feb
Mar
Apr
May
Jun
Jul

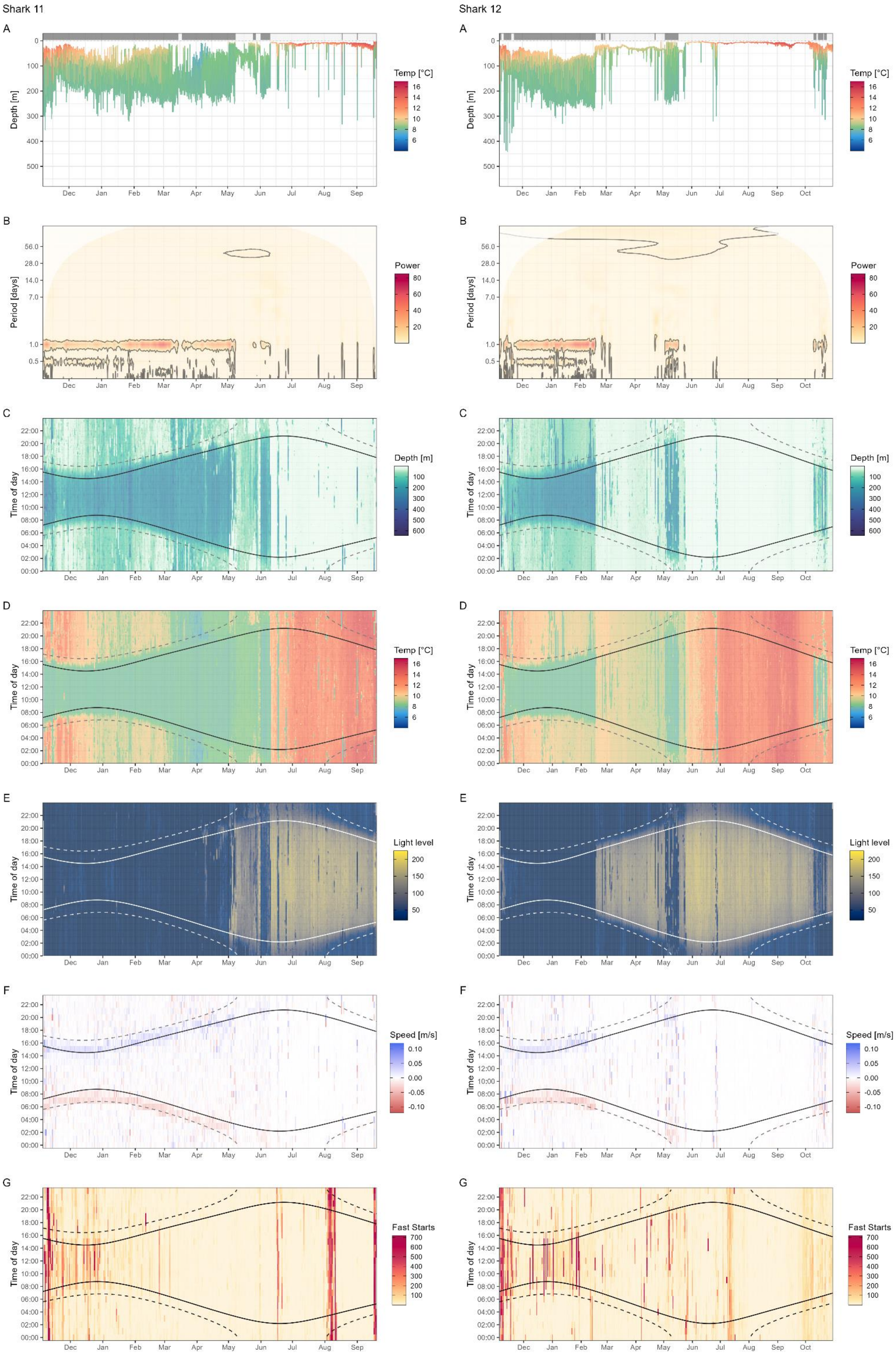

Shark 11
Shark 12
A
B
C
D
E
F
G
Depth [m]
Temp [°C]
Period [days]
Power
Time of day
Depth [m]
Light level
Speed [m/s]
Fast Starts
Dec
Jan
Feb
Mar
Apr
May
Jun
Jul
Aug
Sep
Oct

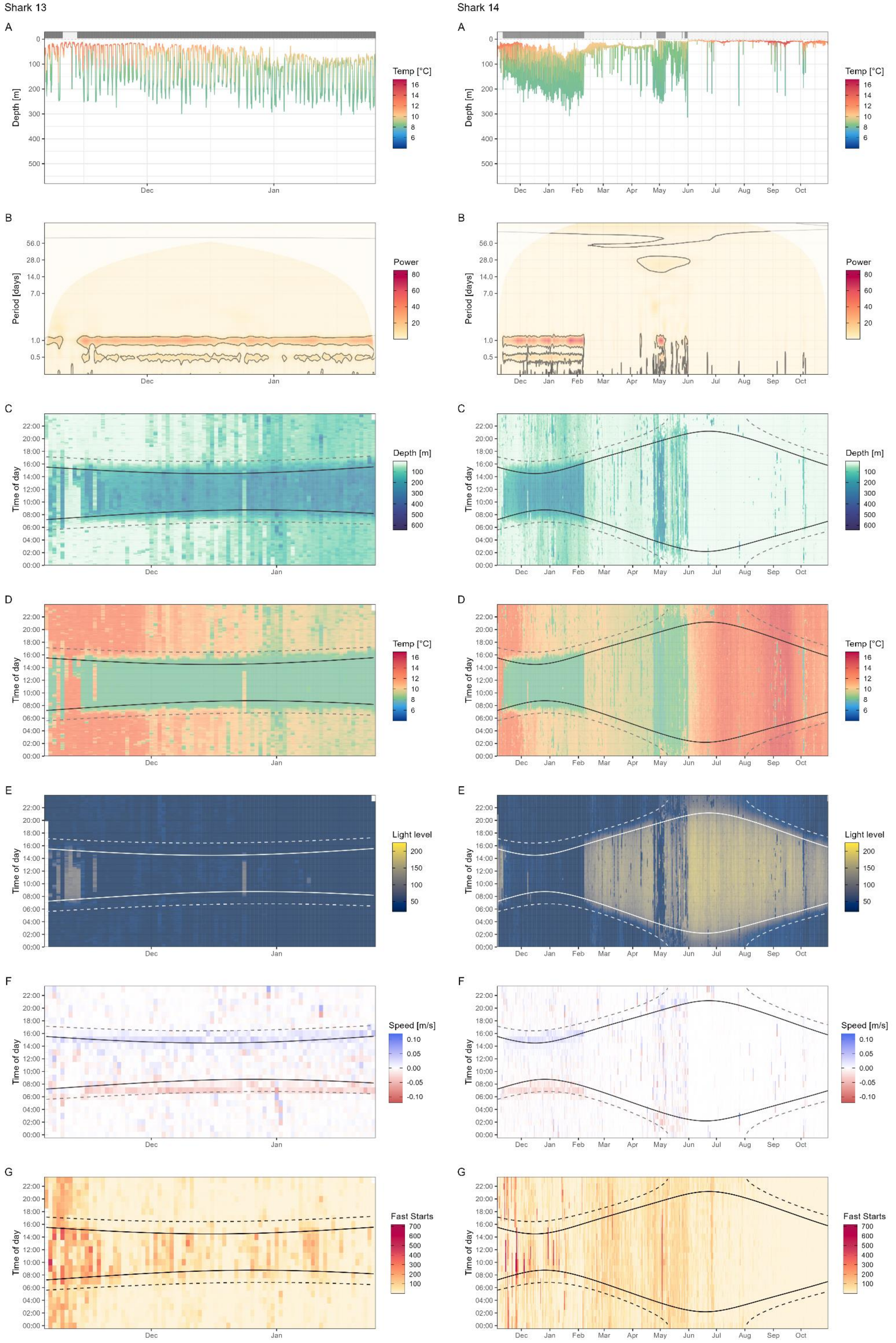

Shark 13
Shark 14
A
Depth [m]
Temp [°C]
16
14
12
10
8
6
Dec
Jan
Feb
Mar
Apr
May
Jun
Jul
Aug
Sep
Oct
B
Period [days]
Power
80
60
40
20
C
Time of day
Depth [m]
100
200
300
400
500
600
D
E
Light level
200
150
100
50
F
Speed [m/s]
0.10
0.05
0.00
-0.05
-0.10
G
Fast Starts
700
600
500
400
300
200
100

Shark 15

Shark 16

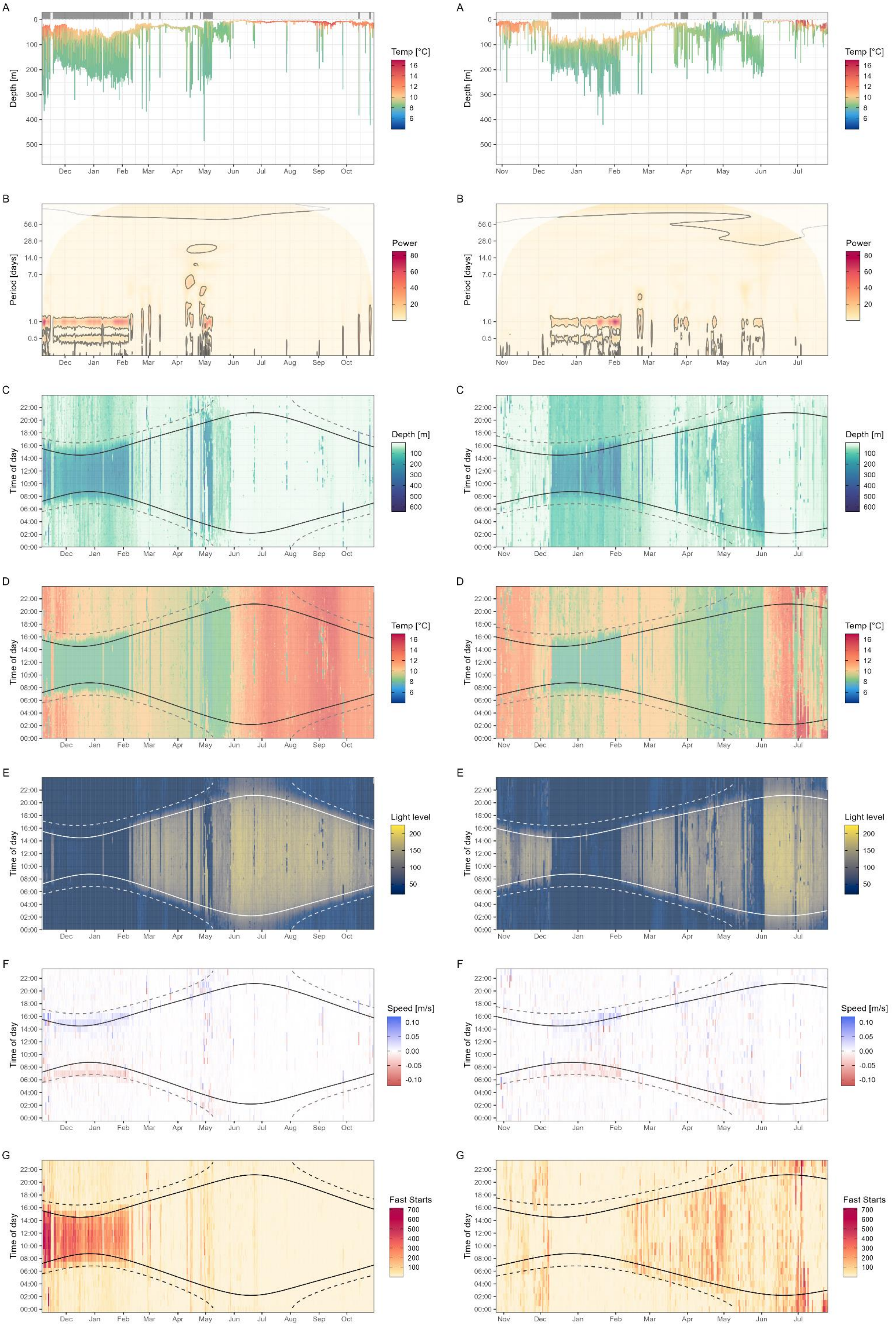

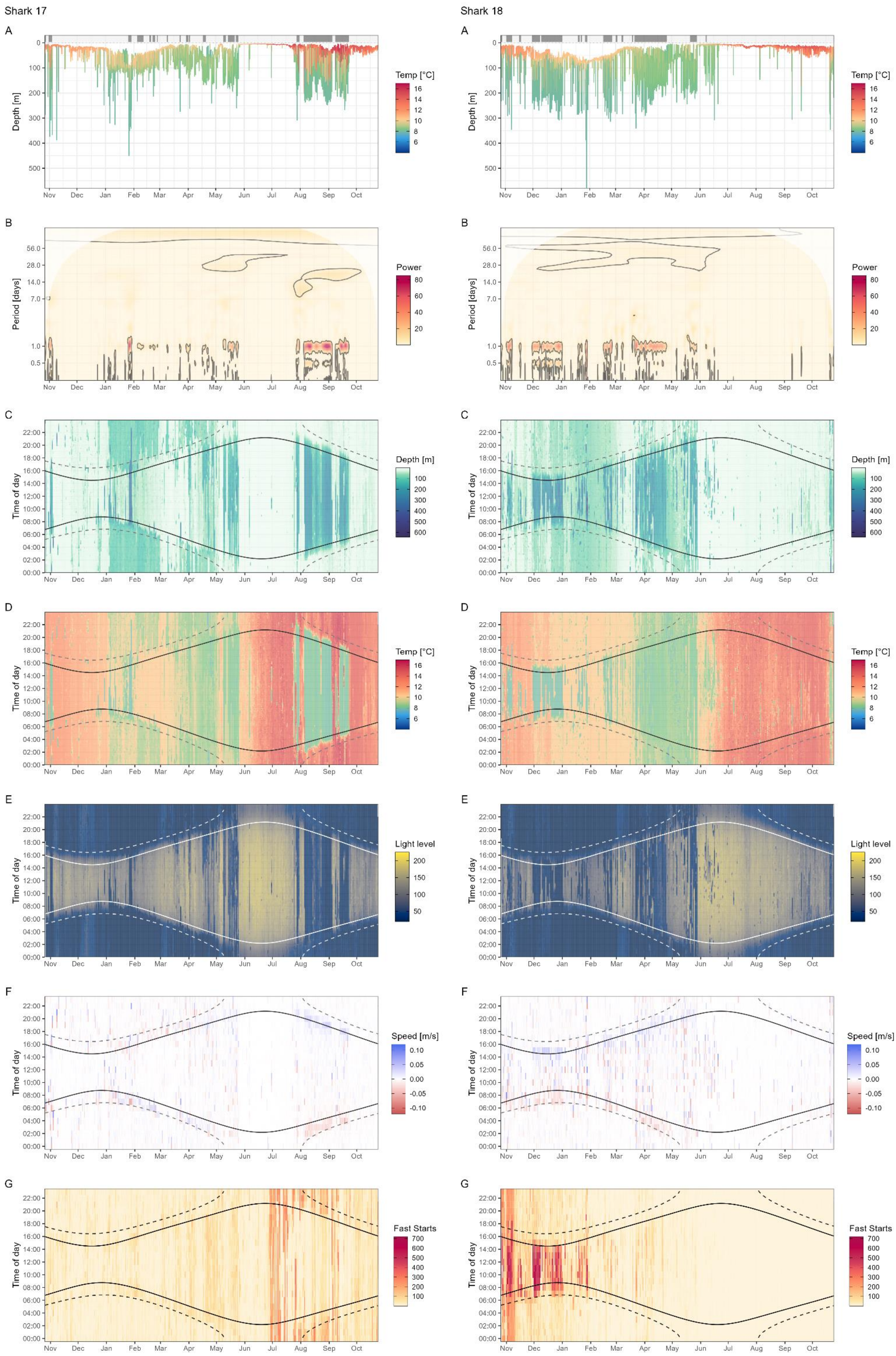

Shark 17
Shark 18
A
Depth [m]
Temp [°C]
B
Period [days]
Power
C
Time of day
Depth [m]
D
Temp [°C]
E
Light level
F
Speed [m/s]
G
Fast Starts
Nov
Dec
Jan
Feb
Mar
Apr
May
Jun
Jul
Aug
Sep
Oct

Shark 19

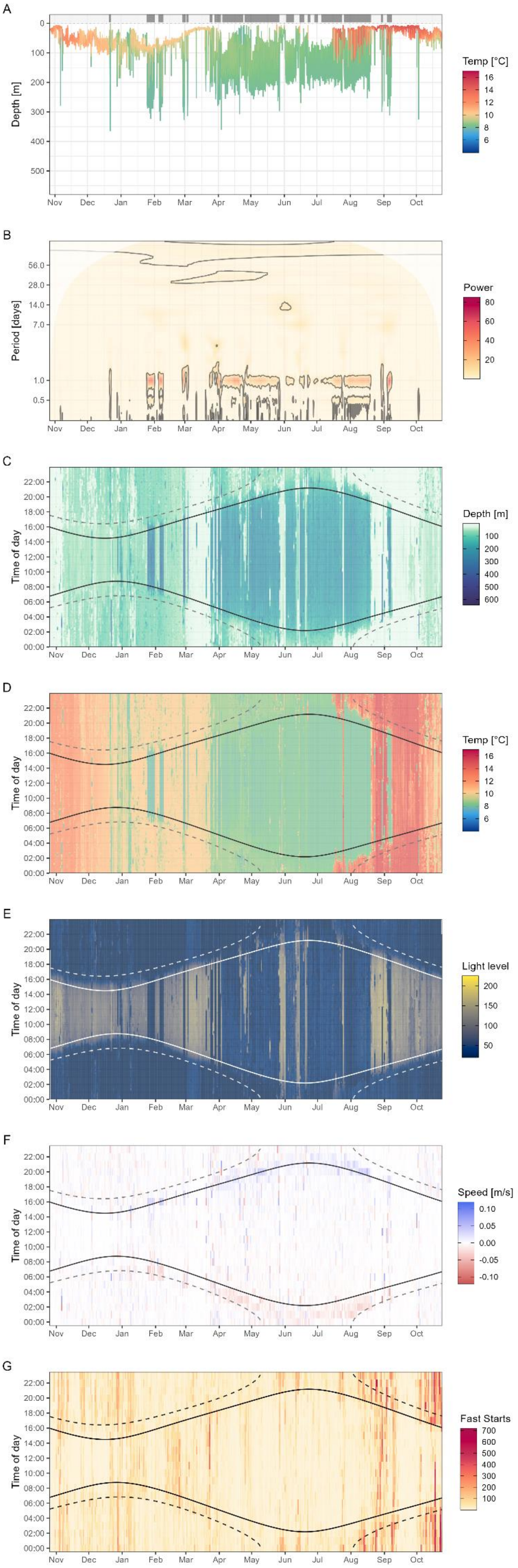

A
Depth [m]
Temp [°C]
16
14
12
10
8
6
0
100
200
300
400
500
Nov
Dec
Jan
Feb
Mar
Apr
May
Jun
Jul
Aug
Sep
Oct
B
Period [days]
56.0
28.0
14.0
7.0
1.0
0.5
Power
80
60
40
20
C
Time of day
Depth [m]
100
200
300
400
500
600
22:00
20:00
18:00
16:00
14:00
12:00
10:00
08:00
06:00
04:00
02:00
00:00
D
Temp [°C]
E
Light level
200
150
100
50
F
Speed [m/s]
0.10
0.05
0.00
-0.05
-0.10
G
Fast Starts
700
600
500
400
300
200
100

Figure S4: Vertical habitat use across deployment period for each shark. (A) Hourly median depth timeseries and with colour coded temperatures. The upper bar indicates the presence (dark grey) or absence (light grey) of diel vertical migration (DVM) behaviour, based on significance ($p \leq 0.05$) of wavelet powers at 24h. (B) Wavelet scalogram based on median hourly depth. Significant powers ($p \leq 0.05$) are highlighted with grey contours. (C-G) Actograms for 1-minute interval median depth (C), temperature (D), and light level (E) use, hourly vertical speed (E), and hourly cumulated fast starts (F) across the time of day. In (D) red colours mark descents, while blue colours represent ascents. Solid lines indicate times of sunrise and sunset, dashed lines nautical dusk and dawn associated with the tagging location. Note the different date ranges between sharks on the x-axis. A description of how light levels correspond to light intensity is provided in Detail S1.

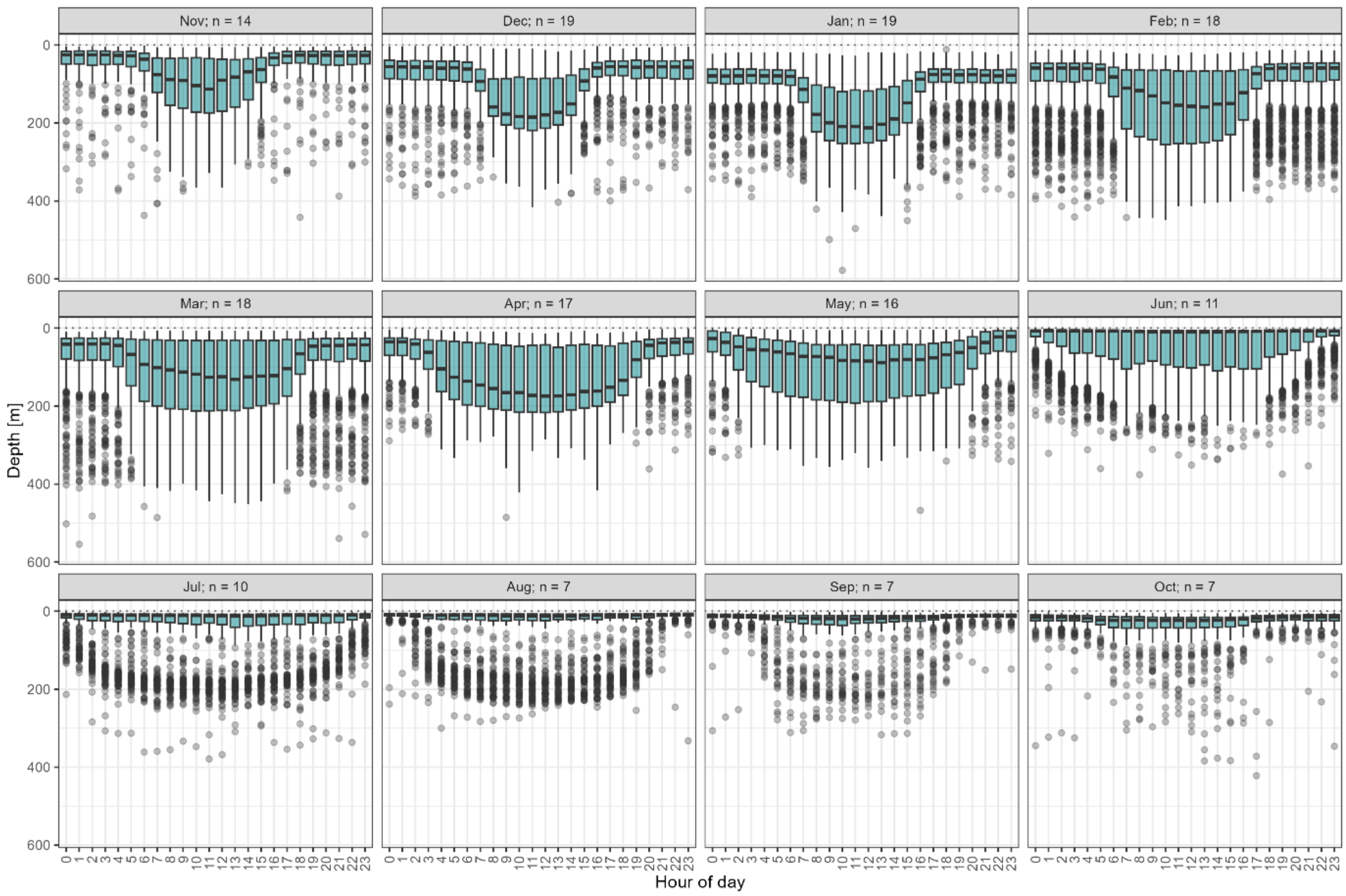

Figure S10: Hourly median depth used across all individuals for a given month. Medians and 25, 75% confidence intervals are shown. Number of sharks contributing to each monthly plot are indicated.

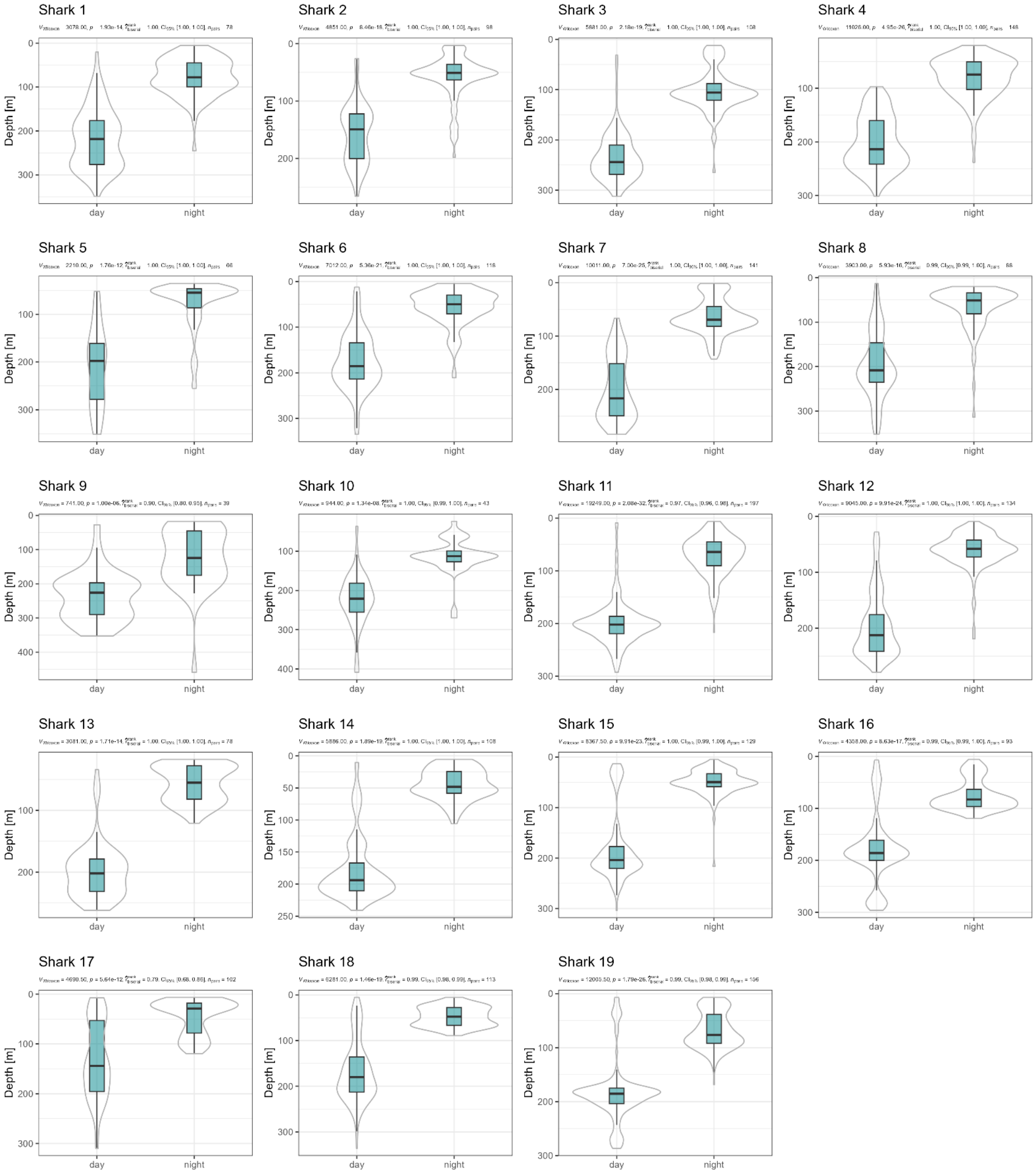

*Figure S5: Inter-individual differences in median depth distributions for day and night during as significant classified diel vertical migration (DVM). Results from a Wilcoxon signed rank test shown for each shark. Violin plots in grey represent the full distribution of the data. Boxplots show median and lower and upper quartiles with whiskers extending to 1.5*IQR.*

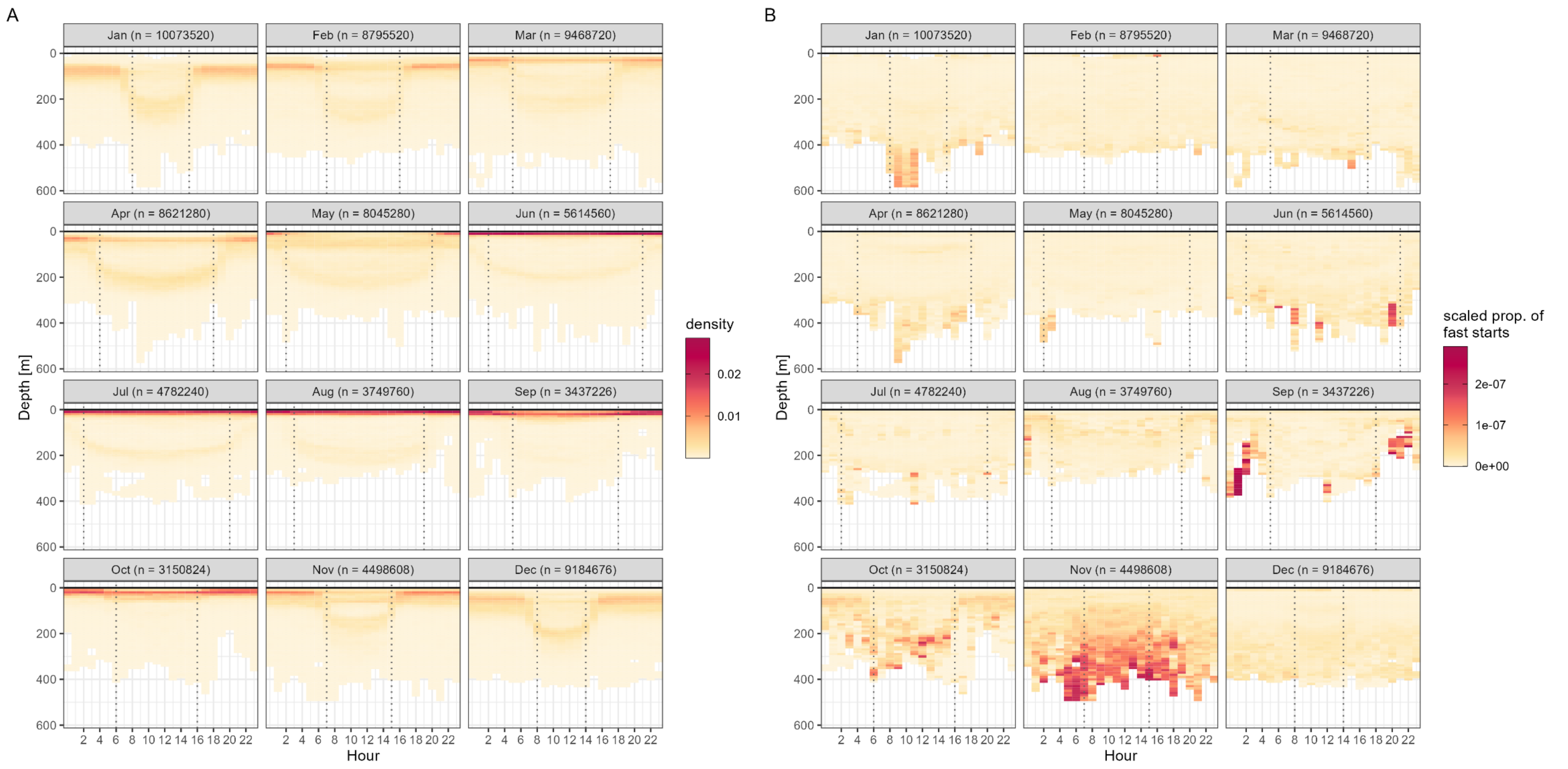

Figure S12: Vertical occupancy (A) and activity (B) landscapes for each month based on 5-sec resolution PSAT data with the first 24h after tagging removed (as in all other analysis). Data are aggregated by month in 10m depth bins and hour of day. The number of data points per month by which data was scaled is noted in brackets. (A) Occupancy calculated as the number of data points within each depth-time cell divided by the number of data points within the respective month. (B) Activity calculated as the proportion of fast starts within each depth-time cell divided by the number of data points per month. Dotted vertical lines mark the median hour of sunrise and sunset for a given month.

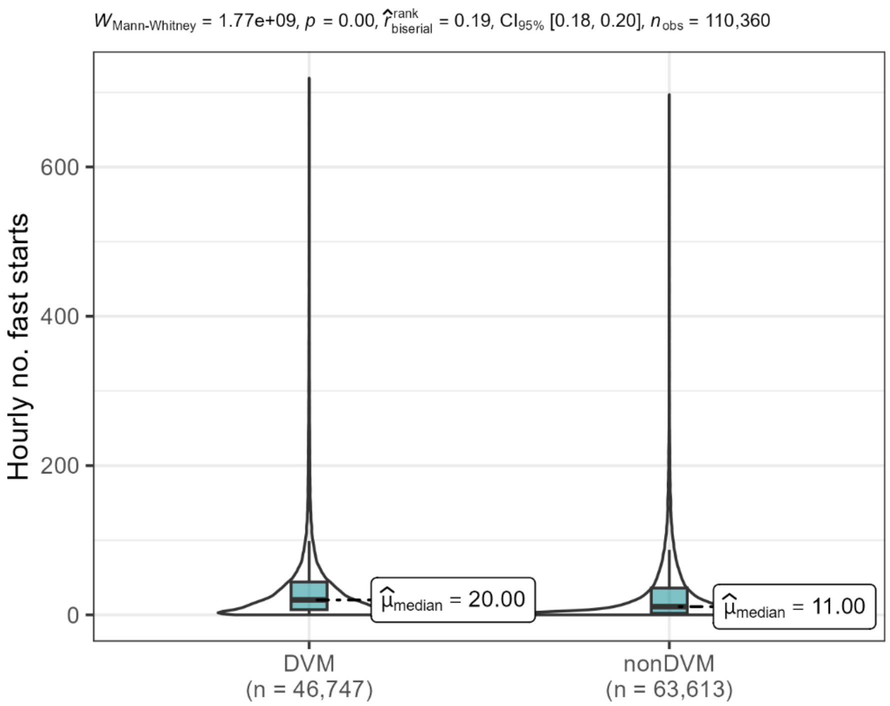

Figure S6: Cumulated hourly fast starts based on all individuals and time periods for diel vertical migration (DVM) and non-DVM behaviour. Violin plots in grey represent the full distribution of the data. Boxplot show median and lower and upper quartiles with whiskers extending to 1.5*IQR.

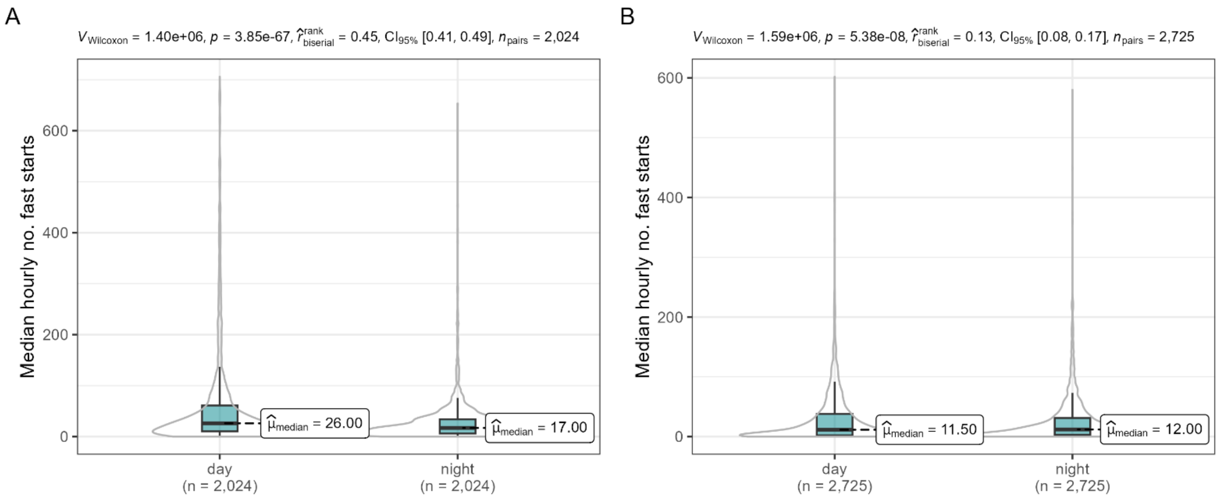

*Figure S7: Median hourly number of fast starts paired for each day and night per individual (A) for as significant diel vertical migration (DVM) classified behaviour and (B) as non-DVM classified behaviour. Violin plots in grey represent the full distribution of the data. Boxplot show median and lower and upper quartiles with whiskers extending to 1.5*IQR.*

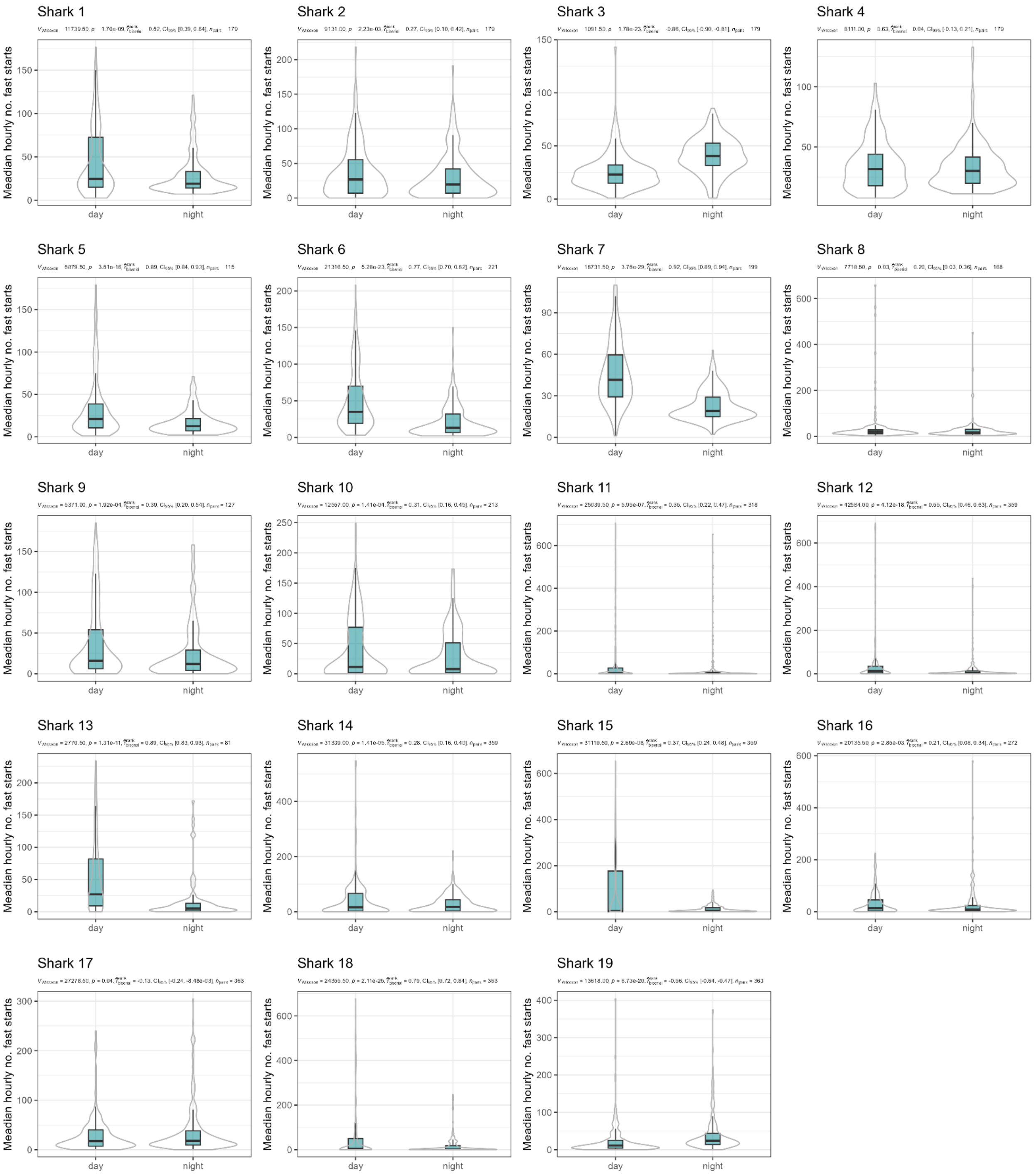

*Figure S8: Inter-individual differences in median hourly cumulated fast start distributions for day and night. Results from a Wilcoxon signed rank test are shown for each individual shark. Violin plots in grey represent the full distribution of the data. Boxplot show median and lower and upper quartiles with whiskers extending to 1.5*IQR.*

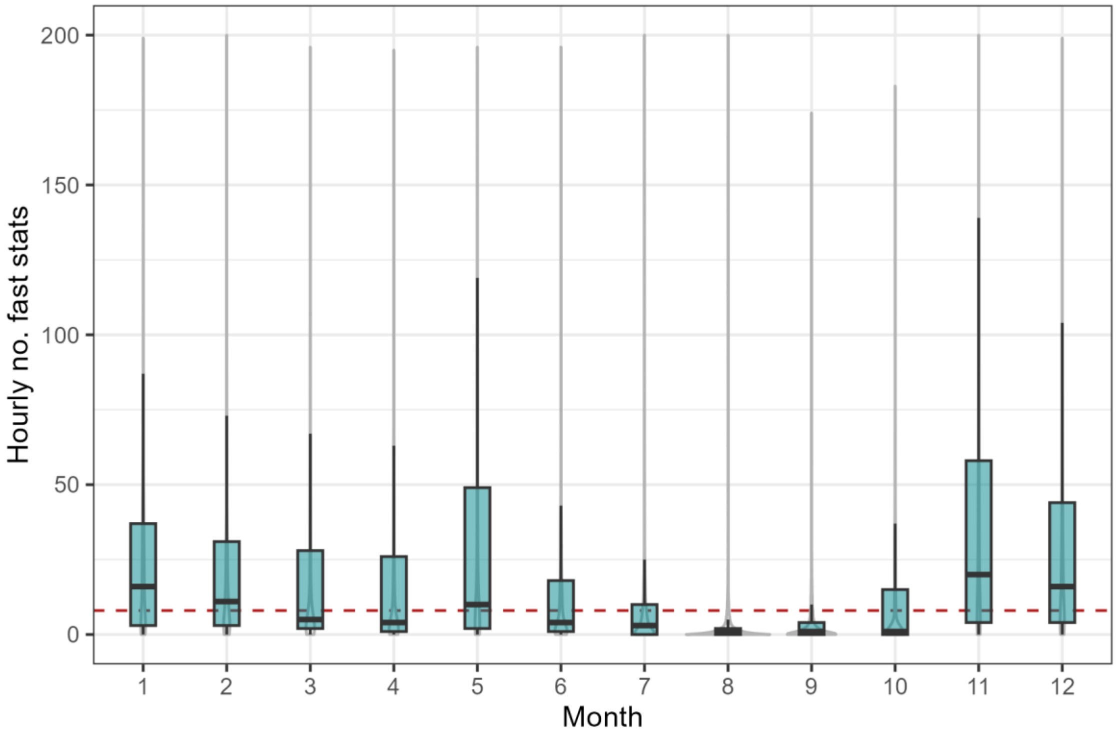

*Figure S9: Differences in hourly cumulated fast starts per months for shark 11,12,14,15,17,18, and 19 which covered a minimum of 320 days. The red dashed line marks the population median (8 fast starts) of the seven individuals across months. The y-axis was limited to 200m for visualisation purposes. Violin plots in grey represent the full distribution of the data. Boxplot show median and lower and upper quartiles with whiskers extending to 1.5*IQR.*

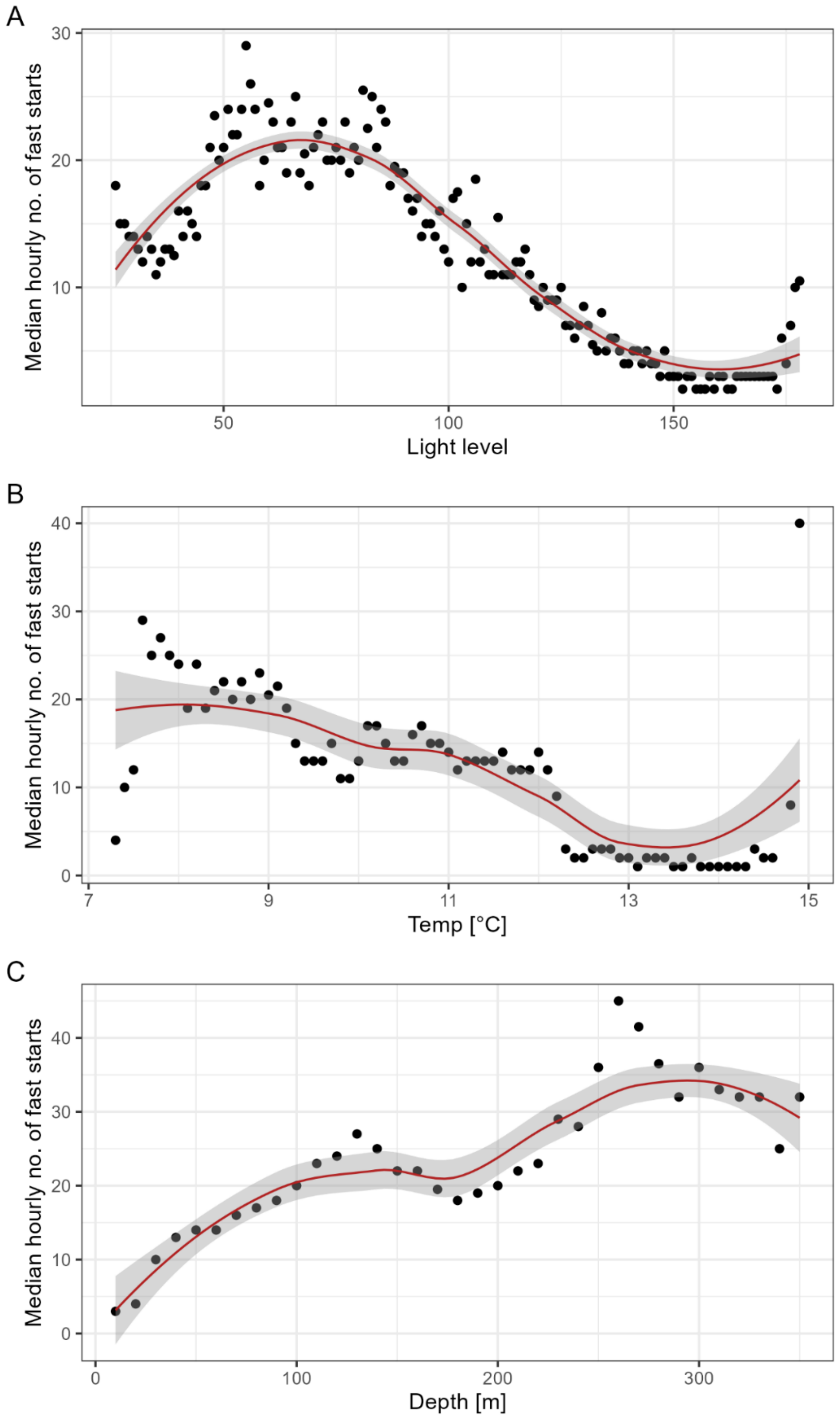

Figure S10: Environmental relationships of light level (A), temperature (B) and depth (C) on the median hourly number of fast starts. Medians of fast starts were taken across bins of 1 light level unit, 0.1°C, and 10m respectively. Bins with n<100 are disregarded. Red line marks a fitted loess function with span = 0.75. 95% confidence intervals shown in grey. A description of how light levels correspond to light intensity is provided in Detail S1.

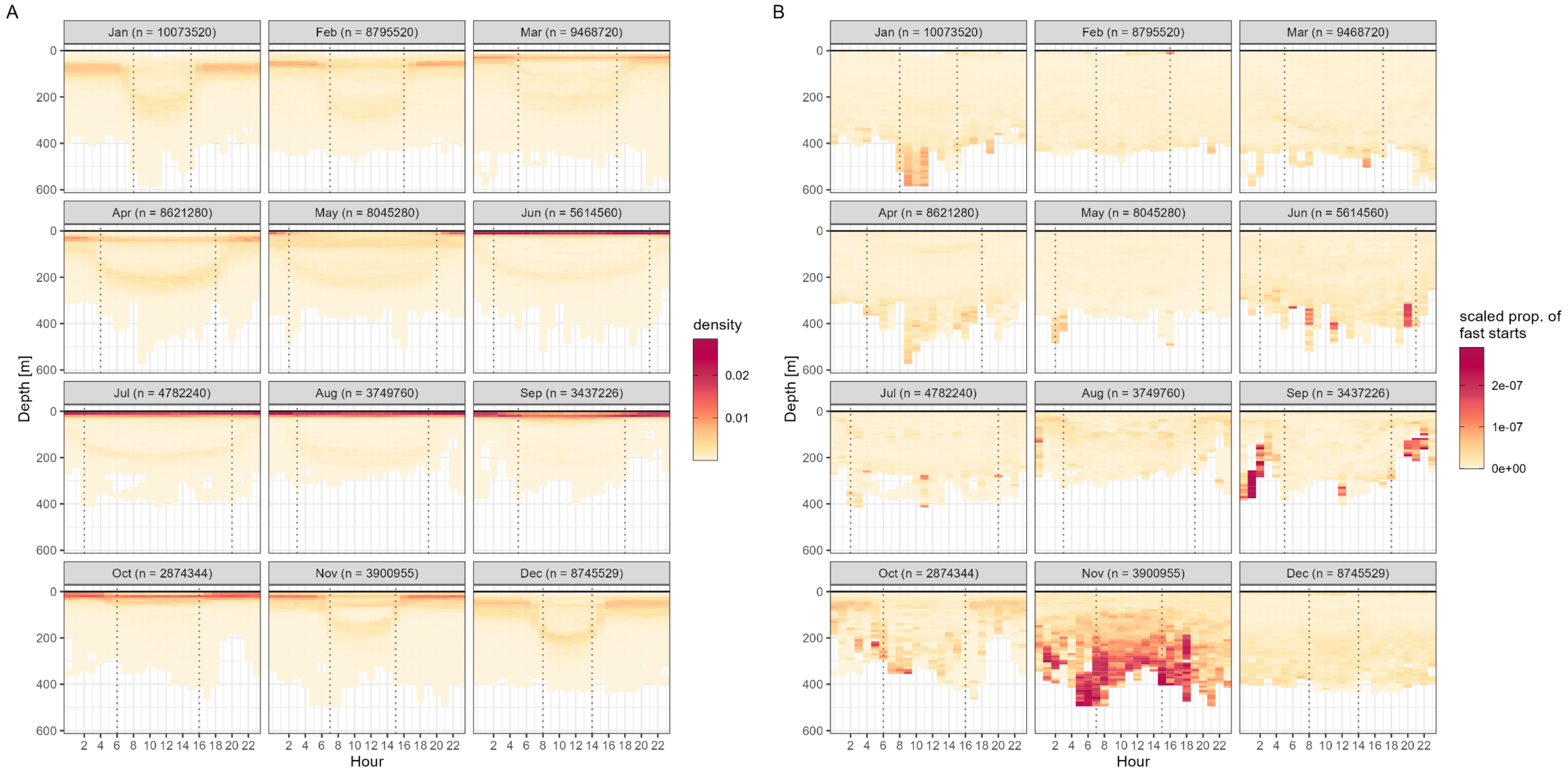

Figure S11: Vertical occupancy (A) and activity (B) landscapes for each month based on 5-sec resolution PSAT data with the first 120h (5 days) after tagging removed. Data are aggregated by month in 10m depth bins and hour of day. The number of data points per month by which data was scaled is noted in brackets. (A) Vertical occupancy calculated as the number of data points within each depth-time cell divided by the number of data points within the respective month. (B) Activity calculated as the proportion of fast starts within each depth-time cell divided by the number of data points per month. Dotted vertical lines mark the median hour of sunrise and sunset for a given month.

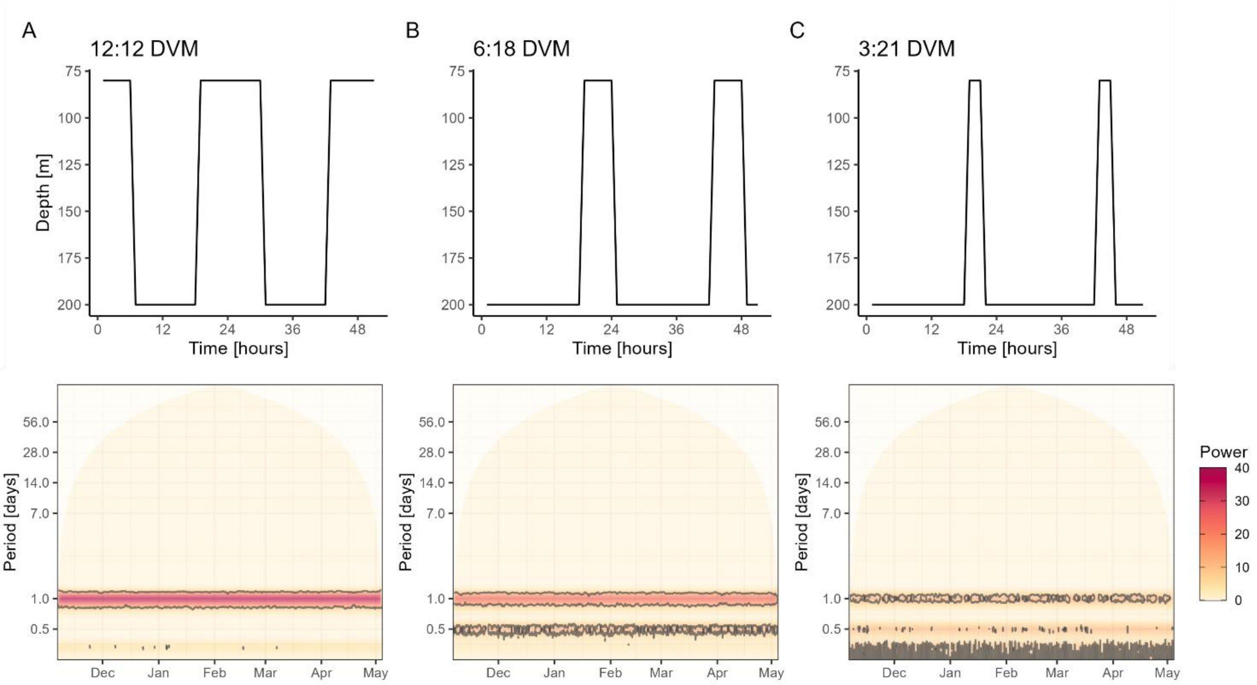

Figure S19: Scalograms obtained from a continuous wavelet analysis with equivalent parameters as used in the main analysis on three synthetic hourly depth time series for fictive 180 days mimicking simplified strict diel vertical movement patterns, alternating between 80 and 200 m depth. Scalogram resulting (A) from a 12:12h pattern, comparable to situations during equinox, (B) from a 6:18h pattern, and (C) from a 3:21h pattern, comparable to day-night patterns during solstice in the study area.

Supplementary References

Abascal, F.J., Quintans, M., Ramos-Cartelle, A., Mejuto, J., 2011. Movements and environmental preferences of the shortfin mako, Isurus oxyrinchus, in the southeastern Pacific Ocean. Mar Biol. https://doi.org/10.1007/s00227-011-1639-1

Aksnes, D.L., Aure, J., Johansen, P.-O., Johnsen, G.H., Vea Salvanes, A.G., 2019. Multi-decadal warming of Atlantic water and associated decline of dissolved oxygen in a deep fjord. Estuar. Coast. Shelf Sci. 228, 106392. https://doi.org/10.1016/j.ecss.2019.106392

Andres, A., 2022. The effects of temperature and oxygen availability on aerobic performance in three coastal shark species; Squalus acanthias, Carcharhinus limbatus, and Carcharhinus leucas (PhD). University of South Florida, Florida.

Cushman-Roisin, B., Svendsen, H., 1983. Internal Gravity Waves in Sill Fjords: Vertical Modes, Ray Theory and Comparison with Observations, in: Gade, H.G., Edwards, A., Svendsen, H. (Eds.), Coastal Oceanography, NATO Conference Series. Springer US, Boston, MA, pp. 373–396. https://doi.org/10.1007/978-1-4615-6648-9_21

Darelius, E., 2020. On the effect of climate trends in coastal density on deep water renewal frequency in sill fjords—A statistical approach. Estuar. Coast. Shelf Sci. 243, 106904. https://doi.org/10.1016/j.ecss.2020.106904

Dell'Apa, A., Cudney-Burch, J., Kimmel, D.G., Rulifson, R.A., 2014. Sexual Segregation of Spiny Dogfish in Fishery-Dependent Surveys in Cape Cod, Massachusetts: Potential Management Benefits. Trans. Am. Fish. Soc. 143, 833–844. https://doi.org/10.1080/00028487.2013.869257

Ford, E., 1921. A Contribution to Our Knowledge of the Life-Histories of the Dogfishes Landed at Plymouth. J. Mar. Biol. Assoc. U. K. 12, 468–505. https://doi.org/10.1017/S0025315400006317

Kaartvedt, S., Røstad, A., Klevjer, T.A., 2009. Sprat Sprattus sprattus can exploit low oxygen waters for overwintering. Mar Ecol Prog Ser 390, 237–249.

Kelly, M.L., Murray, E.R.P., Kerr, C.C., Radford, C.A., Collin, S.P., Lesku, J.A., Hemmi, J.M., 2020. Diverse Activity Rhythms in Sharks (Elasmobranchii). J. Biol. Rhythms 35, 476–488. https://doi.org/10.1177/0748730420932066

Nasby-Lucas, N., Dewar, H., Lam, C.H., Goldman, K.J., Domeier, M.L., 2009. White Shark Offshore Habitat: A Behavioral and Environmental Characterization of the Eastern Pacific Shared Offshore Foraging Area. PLoS ONE 4, 1–14. https://doi.org/e8163

Pitcher, G.C., Aguirre-Velarde, A., Breitburg, D., Cardich, J., Carstensen, J., Conley, D.J., Dewitte, B., Engel, A., Espinoza-Morriberón, D., Flores, G., Garçon, V., Graco, M., Grégoire, M., Gutiérrez, D., Hernandez-Ayon, J.M., Huang, H.-H.M., Isensee, K., Jacinto, M.E., Levin, L., Lorenzo, A., Machu, E., Merma, L., Montes, I., Swa, N., Paulmier, A., Roman, M., Rose, K., Hood, R., Rabalais, N.N., Salvanes, A.G.V., Salvatteci, R., Sánchez, S., Sifeddine, A., Tall, A.W., Plas, A.K. van der, Yasuhara, M., Zhang, J., Zhu, Z., 2021. System controls of coastal and open ocean oxygen depletion. Prog. Oceanogr. 197, 102613. https://doi.org/10.1016/j.pocean.2021.102613

Pohl, L., 2023. Movement behaviour and seasonality of the starry smooth-hound shark (Mustelus asterias) in the North Sea (Master Thesis). Ghent University, Ghent.

Rosland, K., 2022. A comparison of the spatial ecology, trophic ecology, and hypoxia tolerance of Velvet belly lanternshark (Etmopterus spinax) and Blackmouth catshark (Galeus melastomus) in Norwegian fjords. University of Bergen, Bergen.

Santos, C.P., Sampaio, E., Pereira, B.P., Pegado, M.R., Borges, F.O., Wheeler, C.R., Bouyoucos, I.A., Rummer, J.L., Frazão Santos, C., Rosa, R., 2021. Elasmobranch Responses to Experimental Warming, Acidification, and Oxygen Loss—A Meta-Analysis. Front. Mar. Sci. 8.

Shepherd, T., Page, F., Macdonald, B., 2002. Length and sex-specific associations between spiny dogfish (Squalus acanthias) and hydrographic variables in the Bay of Fundy and Scotian Shelf. Fish. Oceanogr. 11, 78–89. https://doi.org/10.1046/j.1365-2419.2002.00191.x

Sims, D., Nash, J., Morritt, D., 2001. Movements and activity of male and female dogfish in a tidal sea lough: alternative behavioural strategies and apparent sexual segregation. Mar. Biol. 139, 1165–1175. https://doi.org/10.1007/s002270100666

Sims, D.W., 2019. The significance of ocean deoxygenation for Elasmobranchs, in: Ocean Deoxygenation: Everyone's Problem, IUCN. IUCN, Gland, Switzerland.

Sims, D.W., 2006. Differences in habitat selection and reproductive strategies of male and female sharks, in: Ruckstuhl, K., Neuhaus, P. (Eds.), Sexual Segregation in Vertebrates. Cambridge University Press, Cambridge, pp. 127–147. https://doi.org/10.1017/CBO9780511525629.009

Skubel, R.A., Wilson, K., Papastamatiou, Y.P., Verkamp, H.J., Sulikowski, J.A., Benetti, D., Hammerschlag, N., 2020. A scalable, satellite-transmitted data product for monitoring high-activity events in mobile aquatic animals. Anim. Biotelemetry 8, 34. https://doi.org/10.1186/s40317-020-00220-0

Søvik, G., Falkenhaug, T., Zimmermann, F., Gallo, N.D., Nedreaas, K., Danre, J.-B., Hovland, T., Thangstad, T.H., Olsen, S.A., Johansen, E., Asplin, L., 2023. Toktrapport fra Økosystemtokt i Vestlandsfjordene (Cruise Report No. 11). Institute of Marine Research, Bergen.

Stehlink, L.L., 2007. Essential fish habitat source document. Spiny dogfish, Squalus acanthias, life history and habitat characteristics, NOAA technical memorandum NMFS-NE; 203. Northeast Fisheries Science Center (U.S.).

Swenson, K.E., Eveland, R.L., Gladwin, M.T., Swenson, E.R., 2005. Nitric Oxide (NO) in normal and hypoxic vascular regulation of the spiny dogfish, Squalus acanthias. J. Exp. Zoolog. A Comp. Exp. Biol. 303A, 154–160. https://doi.org/10.1002/jez.a.145

Vedor, M., Queiroz, N., Mucientes, G., Couto, A., Costa, I. da, Santos, A. dos, Vandeperre, F., Fontes, J., Afonso, P., Rosa, R., Humphries, N.E., Sims, D.W., 2021. Climate-driven deoxygenation elevates fishing vulnerability for the ocean's widest ranging shark. eLife 10, e62508. https://doi.org/10.7554/eLife.62508

Wearmouth, V.J., Sims, D.W., 2008. Chapter 2 Sexual Segregation in Marine Fish, Reptiles, Birds and Mammals: Behaviour Patterns, Mechanisms and Conservation Implications, in: Advances in Marine Biology. Academic Press, pp. 107–170. https://doi.org/10.1016/S0065-2881(08)00002-3

Zimmer, A.M., Wood, C.M., 2014. Exposure to Acute Severe Hypoxia Leads to Increased Urea Loss and Disruptions in Acid-Base and Ionoregulatory Balance in Dogfish Sharks (Squalus acanthias). Physiol. Biochem. Zool. 87, 623–639. https://doi.org/10.1086/677884
